# Data Exploration for Target Predictions Using Proprietary and Publicly Available Data Sets

**DOI:** 10.1021/acs.chemrestox.4c00347

**Published:** 2025-04-20

**Authors:** Aljoša Smajić, Thomas Steger-Hartmann, Gerhard. F. Ecker, Anke Hackl

**Affiliations:** † Department of Pharmaceutical Sciences, University of Vienna, Vienna 1090, Austria; ‡ Bayer AG, Pharmaceuticals Division, Berlin 13353, Germany

## Abstract

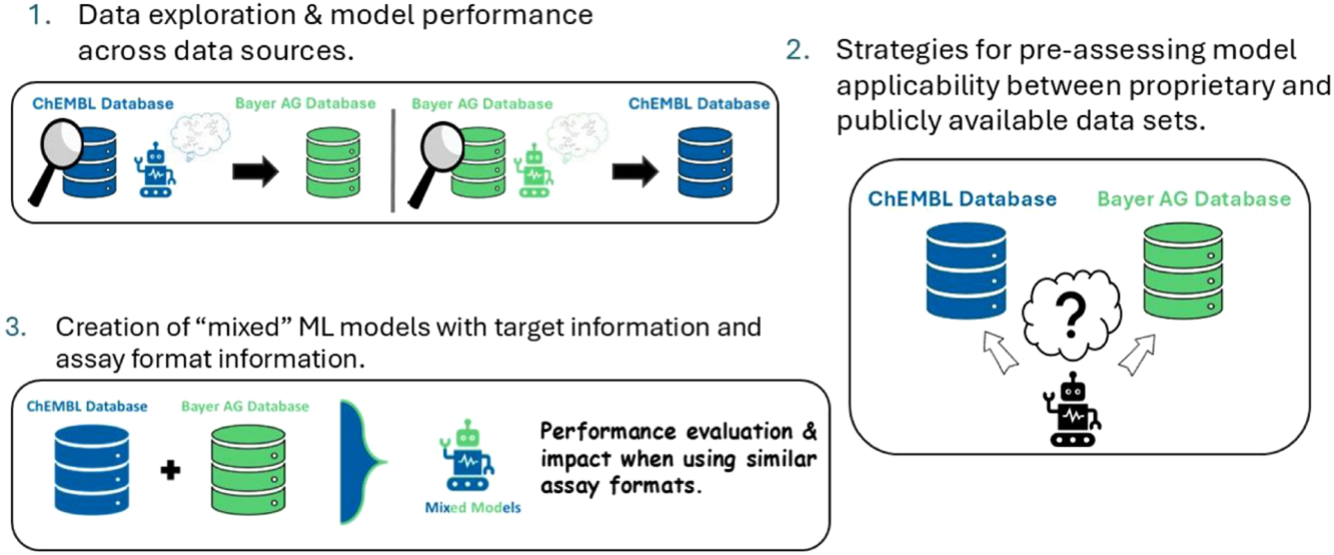

When applying machine learning (ML) approaches for the prediction of bioactivity, it is common to collect data from different assays or sources and combine them into single data sets. However, depending on the data domains and sources from which these data are retrieved, bioactivity data for the same macromolecular target may show a high variance of values (looking at a single compound) and cover very different parts of the chemical space as well as the bioactivity range (looking at the whole data set). The effectiveness and applicability domain of the resulting prediction models may be strongly influenced by the sources from which their training data were retrieved. Therefore, we investigated the chemical space and active/inactive distribution of proprietary pharmaceutical data from Bayer AG and the publicly available ChEMBL database, and their impact when applied as training data for classification models. For this end, we applied two different sets of descriptors in combination with different ML algorithms. The results show substantial differences in chemical space between the two different data sources, leading to suboptimal prediction performance when models are applied to domains other than their training data. MCC values between −0.34 and 0.37 among all targets were retrieved, indicating suboptimal model performance when models trained on Bayer AG data were tested on ChEMBL data and vice versa. The mean Tanimoto similarity of the nearest neighbors between these two data sources indicated similarities for 31 targets equal to or less than 0.3. Interestingly, all applied methods to assess overlap of chemical space of the two data sources to predict the applicability of models beyond their training data sets did not correlate with observed performances. Finally, we applied different strategies for creating mixed training data sets based on both public and proprietary sources, using assay format (cell-based and cell-free) information and Tanimoto similarities.

## Introduction

Machine learning (ML) along with other artificial intelligence (AI) methods have experienced rapid advancement in the field of drug discovery and development.^1,2^ Its significance can be evident in shaping the design of novel compounds of medical relevance and synthetic routes for medicinal chemistry.^3^ As an example, quantitative structure–activity relationship (QSAR) approaches serve as a powerful instrument for *in silico* toxicity and bioactivity predictions.^4^ The predictive performance of these statistical methods relies heavily on the quality of the data from which the relationship between chemical properties and activity has to be learned.^5,6^ Moreover, the availability of data also has a major influence on the predictivity and applicability of the models.^7^

Generally, data sets for computational drug discovery approaches are generated from different assays, experimental settings, and organizations.^8,9^ In industry settings, large, consistently measured data sets are common. These data sets are properly curated and can be retrieved from large data repositories within the companies. Moreover, these data sets bear detailed annotations which are crucial when interpreting and contextualizing bioactivities. With consistent measurements and detailed annotations, data integrity and reliability can be assessed and ensured. As an example, one can determine the assay type (e.g., radiological assay, in vitro/in vivo), allowing data sets to be combined accordingly and applied for specific tasks. However, proprietary data sets are nondisclosed to the public and therefore limited to the use within their own company.^10^

In contrast, public databases are freely accessible and allow for widespread access to information related to bioactivity measurements.^11,12^ Moreover, using public databases reduces the financial burden on researchers and public institutions due to high investment associated with data generation. In sum, they can be used freely, openly and without charge, making them an often-preferred choice among academic researchers and research scientists.^13^ Despite these benefits, public databases are few and sparse compared to industry settings. They do not offer consistent comparable measurements and annotations as within industry settings. An additional drawback is a significant decrease in size when data sets are taken from a single experimental setup.^14^ Therefore, a typical strategy for creating data sets for ML approaches in the public domain is to combine results from different assays and sources, solely based e.g., on their annotated macromolecular target.^15^

Recent work by Landrum and Riniker^15^ demonstrated that combining IC_50_ or K_i_ values from different sources introduces a significant amount of noise in the data sets and resulting ML models. Another drawback demonstrated by Smajić et al.^16^ identified a significant bias in which models built from publicly available data sources tend to falsely predict many compounds to be active and models built on proprietary sources to falsely predict many compounds to be inactive. In that study, the ChEMBL database was examined to analyze the distribution of active and inactive compounds and demonstrated that the database contains an imbalance toward more active than inactive compounds for most data sets. In addition, the study showed that models generated from the publicly available database are prone to overpredict active compounds due to this imbalance and that this effect can be mitigated to some extend by using consensus predictions from these models and additional models based on data from proprietary sources. As in the study of by Smajić et al. the ML models from the Roche off-target panel were provided and no access to the data was given, a more thorough data investigation and comparison was not possible.

Therefore, we were interested in the predictivity of models when trained on a publicly available data set but tested on a Bayer AG data set and vice versa. Additionally, we investigated how representative the chemical spaces of the two available data sources, proprietary and publicly available, are of each other. Moreover, by comparing the chemical and also property spaces of the data sources together with details on the experiments from which the measurements were obtained, we examined the most effective approach for handling the data from different data set sources. This can help to guide decision-making in terms of enhancing sparse data sets and applicability of ML models for off-target and bioactivity predictions.^17^

In this work we conducted an extensive data analysis using internal data from Bayer AG and from ChEMBL to identify differences between the two data domains (public and proprietary) for 40 targets and their impact on ML models. In addition, we applied two different sets of descriptors (electrotopological state (Estate)^18^ and continuous data driven descriptors (CDDDs)^19,20^ and investigated their influence on classical ML algorithms such as Random Forest (RF), XGBoost (XGB), and Support Vector Machine (SVM) for these tasks. Moreover, we analyzed the differences in the chemical space covered by Bayer AG and ChEMBL data for the 40 investigated targets based on Uniform Manifold Approximation and Projection (UMAP)^21^ representations and using mean Tanimoto similarity as well as the distribution of classical physicochemical properties such as Molecular Weight, H-Bond Acceptor and Donor Count, Heavy Atom Count, Rotatable Bond Count, Topological Polar Surface Area and predicted logD at pH 7.5 between these two data sources. Finally, we applied different strategies for merging data sets using assay format (cell-based and cell-free) information and Tanimoto similarities for ML model building.

## Methods

### Data Set Preparation from Bayer AG and ChEMBL

All target entries listed within the internal database from Bayer AG were explored, and only the UniProtKB accession numbers (ACs)^22^ with the organism annotation *Homo sapiens* were collected.

In total 40 human targets for which sufficient data are available could be retrieved from both data sources (Bayer AG and ChEMBL). The criteria the data had to fulfill were ≥ 250 data points in total and at least 15% of the minority class represented in the target data set for both domains. Classes were defined based on a threshold of IC50 or *K*_i_ value of 10 μM. A data set size of 250 has been chosen as it can be considered a reasonable number for training ML algorithms such as SVM, RF and XGB.^23^ Data on the targets that fulfilled the criteria were selected to undergo a standardization protocol. Moreover, for each compound the InChI (IUPAC International Chemical Identifier), InChI Key and SMILES (Simplified Molecular Input Entry Specification) were calculated. MolVS (version 0.1.1) was applied to remove stereochemistry information, salts, neutralize, and discard nonorganic compounds.

To ensure data consistency, if in ChEMBL multiple measurements of a compound’s interaction with the target of interest were identified, that lead to different classification (i.e., at least 1 measurement claiming activity and 1 measurement claiming inactivity), this compound was removed from this target’s data set. On the other hand, if a compound with multiple measurements showed activity within the Bayer AG data sets, the active class was assigned in any case due to more consistent data management within the database. This cautious approach was chosen, because in context of off-target predictions, false positive predictions are considered acceptable while false negative predictions state a higher risk of missing a dangerous effect.

**Figure 1:**
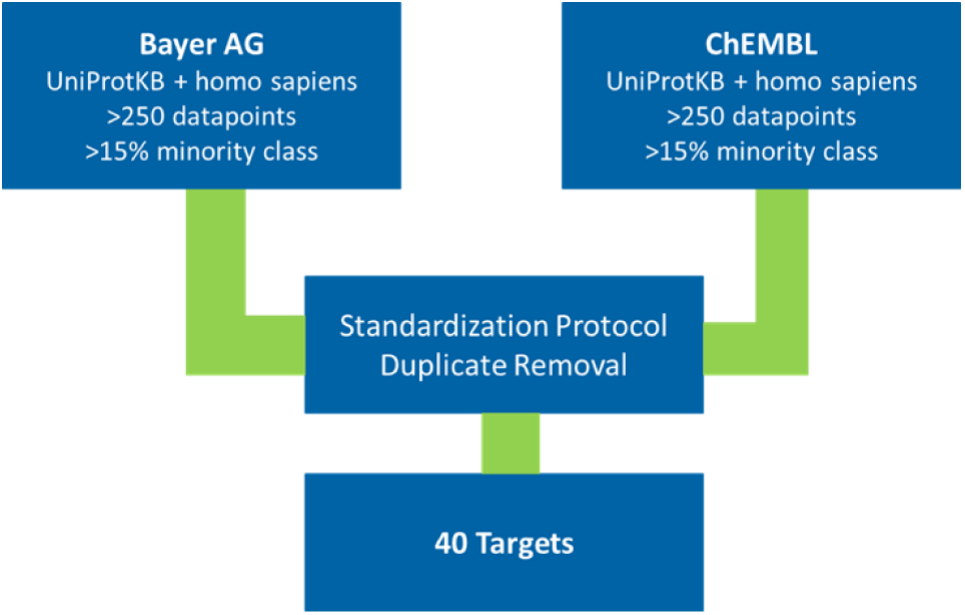
Depiction of the workflow for the retrieval of the data sets for the 40 Targets. It illustrates that both data sources were used and UniProtKB accession numbers (ACs)^22^ with the organism annotation *Homo sapiens* and the criteria to fulfill were ≥ 250 data points and at least 15% of the minority class represented in the target data set for both domains were applied.

As a result, sufficient data for a total of 40 targets were retrieved (Figure 1).

### Data Set Preparation for Mixed Models with Assay Format Information

Considering the experimental setup in which bioactivity data was generated, in addition to the investigated target, is expected to improve model performance. To that end, we created data sets from both data sources that contained results either measured in a cell-based or a cell-free assay format.

For Bayer AG data sets a dedicated annotation on the assay format (cell-based or cell-free) was available, while ChEMBL data sets do not provide such an explicit annotation. Therefore, individual data sets from ChEMBL with cell-based experimental information were created by leveraging the combination of annotations on in vitro and cell name annotation entries. In addition, assay type information such as Binding (B), ADME (A) and Toxicity (T) were further utilized. For the creation of cell-free experimental data sets from ChEMBL, a similar approach has been conducted by removing entries with annotated cell lines from ChEMBL data and keeping assay type information: A, B, T. These annotations were used to retrieve combined data sets from both sources that can be used to train mixed models.

Three different approaches to compile the mixed training data were applied:

a. Considering only the individual target information
b. Considering target information and assay format
c. Considering target information, assay format and Tanimoto similarity ≥ 0.230^24^

This investigation was performed on the targets KCNH2 and PDGFRA, because for these two targets both data sources contained sufficient data points with the necessary annotations. The mixed models for KCNH2 following approaches b) and c) are based on the assay format “cell based”, while the ones for PDGFRA are based on the assay format “cell free”. At Bayer AG for both targets, there are multiple test definitions (distinct experimental setups) available, resulting in two separate data sets for KCNH2 and three separate data sets for PDGFRA. These were used as basis for training individual ML models, adding ChEMBL data following the mentioned approaches a to c.

A more detailed differentiation of experimental setups (e.g., considering specific cell lines instead of the simple annotation of assay format) is expected to be beneficial but would result in a significant decrease in training data set size. Hence, it was not further investigated here.

Random and cluster based nested CV approaches were chosen for the evaluation of the models. Given the absence of significant differences in the model performance between SVM and XGB models, the latter was selected for ML model building due to its shorter computation time.

Violin plots were created to depict the results of random and cluster-based CV for easier comparison. Thicker sections indicate more consistency in model performance. Moreover, the thickness of the violin plot helps to understand the stability and variability of the model’s performance across different folds. Specifically, a longer violin plot indicates a wider range of MCC values, while a shorter violin plot suggests a more limited range.

### Descriptor Selection

Two different approaches for calculating molecular descriptors were applied: Molconn-Z Estate (Electrotopological State)^18^ and Continuous and Data-Driven Descriptors (CDDDs).^20^ They were selected owing to their different ways of capturing information on the compounds and for an analysis on the impact of these two different ways of chemical structure representation in ML model performance. Estate molecular descriptors capture electrotopological state contributions from neighboring atoms and unique values for different structural features.^18^ Whereas CDDDs incorporate concepts from neural machine translations. CDDDs compress significant information on molecular structures into a low-dimensional representation vector by translating two syntactically distinct but semantically identical representations of the same structures.^20^ Therefore, CDDDs can be considered as structural descriptors, while Estate descriptors can be considered as electrotopological descriptors. In this study, a set of 512 numerical features corresponding to the CDDDs and 79 numerical features representing the Estate descriptors, were utilized, as the scope of the research focused on data set evaluation rather than the creation of best performing models or descriptor analysis and search. Given that CDDDs effectively represent the structural aspects of the compounds, we prioritized them over 2D fingerprint descriptors, as other descriptors would not provide additional insights.^19^

### Statistical Metrics

To estimate the performance of the binary classification the following parameters were used:

- Sensitivity: TP/(TP + FN)
- Specificity: TN/(TN + FP)
- Balanced accuracy: (Sensitivity + Specificity)/2
- Accuracy: (TP + TN)/(TP + FP + TN + FN)
- Precision or Positive Predictivity (PPV): TP/(TP + FP)
- Negative predictivity (NPV): TN/(TN + FN)
- F-Measure: Two * ((precision * recall)/(precision + recall))
- Matthews correlation coefficient (MCC): (TP * TN – FP * FN)/sqrt((TP + FN)(TP + FP)(TN + FP)(TN + FN))

Here, a true active prediction is phrased as true positive (TP), a true inactive prediction as true negative (TN), a false active prediction as false positive (FP) and a false inactive prediction as false negative (FN). To choose the best-performing hyperparameters, the MCC was selected as a statistical metric.^25^

### Model Generation

Three distinct classifiers, namely RF, XGB and SVM were employed for model generation and analysis to assess the influence of different training sets and descriptors. The scikit-learn Python library (version 1.2.2) implementations were used to train binary classification models for the 40 above-mentioned targets.

### Hyperparameter Grid Search

A nonextensive hyperparameter search was performed for each classifier to identify parameters conductive to effective model generalization.

The following parameters were used:

- *Random Forest:* n_estimators: 50, 100, 200, max_depth: None, 10, 20
- *XGBoost*: n_estimators: 50, 100, 200, max_depth: 3, 5, 7
- *Support Vector Machine:* C: 0.1, 1, 10, kernel: linear, rbf

For hyperparameter selection, we utilized nested cross-validation (CV) during model training to identify the optimal set of hyperparameters. Instead of selecting the combination that produced the highest MCC, we chose the hyperparameter set that was most frequently selected across the nested CV iterations. This method is less prone to overfitting compared to more extensive grid searches focusing on the highest MCC.

### Training Procedure and Nested Cross Validation

**Figure 2:**
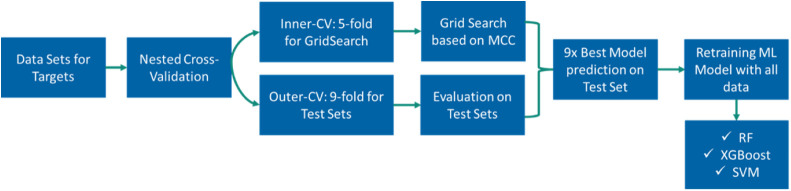
Depiction of the workflow used to generate the models in this study. It illustrates the process of applying the inner and outer CV for hyperparameter search and evaluation of the models. Lastly, the models are retrained with the most frequently occurring hyperparameters and entire data sets for 3 different ML algorithms: rf, xgboost, and svm models.

A nested cross validation (CV) was performed, to systematically evaluate and optimize the performance of the predictive models. This approach involves an outer loop for model evaluation, where the data set is divided into training and test sets, and an inner loop for hyperparameter tuning, where the training set is further partitioned into multiple subsets for model construction and validation. By iteratively performing this process, nested CV allows a thorough evaluation of model generalization across different training and test data sets and robust hyperparameter optimization, enhancing the reliability and validity of the models. In this study we applied a 5-fold inner CV and 9-fold outer CV, whereas for retraining the final ML models, the most frequently occurring hyperparameters from the outer CVs were taken. By choosing a 9-fold outer CV, an odd number of resulting models were created compared to a 10-fold CV, which helped to select the most frequently occurring hyperparameters. This strategy has been conducted for both, ChEMBL and Bayer AG data sets, with the mentioned molecular descriptor sets. The workflow used for the nested CV for ChEMBL and Bayer AG internal data sets is depicted in Figure 2.

In an additional study, combined data sets from different sources were used for model training. Performance was evaluated by applying nested CV using random and cluster-based splitting. Cluster-based splitting was performed using the Butina^26^ algorithm with Tanimoto similarity ≥ 0.230 as a threshold.

The ML models were retrained using all the data, utilizing the most frequently occurring hyperparameters identified from the outer CV results of the nested CV results.

### Comparative Evaluation of ML Models Across Data Sources

Models trained on data from one data source (ChEMBL or Bayer AG) were evaluated on the data from the other source. To avoid data leakage due to overlapping data, compounds present in the training set were filtered out of the respective test set for each target based on the InChIs. This would allow no contamination of the test set which would be further used for the assessment of the models. The final models, ChEMBL and Bayer AG, were applied to their respective test sets from the other data domain: ChEMBL models on the Bayer AG internal data sets, and Bayer AG models on the ChEMBL data sets.

### Visualization of Chemical Space Between Publicly Available and Proprietary Data Sets

A visualization of the chemical space for both data sources for each target was conducted using the dimensionality reduction technique UMAP (Figure 5). This visualization technique allows the projection of molecular structures from a high-dimensional space (i.e., chemical descriptor space, biological descriptor space, etc.) to a 2D plane, preserving the global structure of the data. UMAP can help to identify clusters of similar molecules, detect outliers, and gain insights into the underlying chemical or biological space.^21^ Compounds from one data source were filtered out from the data set from the other source using InChIs as the identifier leading to two data sets: ChEMBL_delta and Bayer AG_delta. The resulting UMAP visualizations can be found in the Supporting Information (Figure S13). UMAP was applied to reduce the dimensionality of the data, using the following parameters: n_neighbors = 15, min_dist = 0.1, and n_components = 2. These settings were chosen to balance local and global structure in the resulting embedding.

### Mean Tanimoto Similarity of the Nearest Neighbors Between ChEMBL and Bayer AG Data Sets

The Tanimoto Similarity is a metric used to compare molecular fingerprints measuring the similarity between two chemical compounds by calculating the ratio of shared features (bits set to 1 in both fingerprints) to the total number of features (bits set to 1 in either fingerprint). It ranges from 0 to 1, with a higher Tanimoto Coefficient indicating greater structural similarity. A Tanimoto Coefficient of 0.8, for example, suggests a significant degree of overlap in features, reflecting substantial molecular resemblance.^27^ The mean Tanimoto Similarity of the nearest neighbors between ChEMBL and Bayer AG data sets were calculated using Morgan Fingerprints 2 from RDKit. For each compound in the Bayer AG data set, we identified the nearest neighbor from the corresponding ChEMBL data set using Tanimoto similarity calculations. When thinking about the enrichment of Bayer data sets from similar compounds from ChEMBL data set, we considered the chemical similarity and chose a threshold of 0.230 as described in the analysis by Landrum et al.^24^ This approach was chosen because it establishes well-founded thresholds for assessing similarity using this Morgen Fingerprints 2 descriptors and similarity metrics. Internal studies at Bayer AG have indicated discrepancies when assessing the structural similarity of compounds represented as CDDDs based on the Tanimoto Coefficient. An analysis of nearest neighbor indicated low similarity between the Bayer AG data sets and ChEMBL which is further discussed in the results section.

## Results

### Data Set Overview

Simple statistical analysis was used to determine the size of minority classes and data sets. Data set sizes for all 40 targets (listed as HGNC symbols) are provided in the Supporting Information (Table S1, Figures S1 and 2). In the majority of the ChEMBL data sets, the active class contains more compounds than the inactive class (Figure S1). In comparison, in the Bayer AG data sets, one can observe a shift toward a higher ratio of inactive compounds (Figure S2). The maximum number of compounds overlapping between ChEMBL and Bayer AG data sets was 10% for the target Serine/Threonine-protein kinase ATR. For 13 targets, there were no compounds from the one data source occurring as duplicates in the other data source. The retrieved data sets from both data sources were further employed in an analysis on mixed ML models, utilizing training data compiled from both sources into one data set as described in the methods section. Starting off with the data set from one distinct experimental setup from Bayer AG, corresponding ChEMBL data were added to the training set. First, by combining the data sets according only to their respective target (approach a). Second, by combining the data sets according to the respective target and the assay format information (approach b), and last by adding only ChEMBL compounds with a Tanimoto similarity ≥ 0.230 according to the respective target and assay format to the corresponding Bayer AG data set (approach c). The more restrictive approaches generally lead to a reduction in training data set size, thus also affecting split size and split heterogeneity in the nested CV during model training. These data sets were used to train ML models in random and cluster based nested CVs to investigate the impact of combined training data sets on model quality. Two targets, KCNH2 and PDGFRA, were chosen for this analysis. Data availability for both data set domains is provided in Supporting Information Table S1.

### Model Performance of Nested CV within One Data Source

To assess the predictive performance of models after the nested cross-validation for the 40 targets, MCC was selected for evaluation in the graphical depiction. Figures S3–S6 represent the aggregated results of the outer CV with the corresponding set of descriptors. Figures S3 and S4 illustrate the performances in combination with CDDDs. Overall, good performances were observed for both data sources and descriptor sets. Models for the majority of targets exceeded a mean MCC over 0.5, which can be translated to a good predictiveness of the models within their training data source.

Moreover, the standard deviation across all outer CV results per target and data domain, which is depicted as error bars for both data sources and descriptor sets (Figures S3–S6), was within an acceptable range, indicating consistency and robustness of the methods toward varying splits in test and training data sets within the respective data source. In addition, all statistical metrics for each target can be downloaded from Zenodo (https://zenodo.org/doi/10.5281/zenodo.14355717).

Results derived based on CDDDs within the ChEMBL source, for the targets CDC25A, CYP2C19, CYP2C8, CYP2C9, MALT1 showed lower performance compared to others with mean MCC values slightly higher than 0.4, indicating still acceptable model performance (see Figure S3). Observing Bayer AG results, only the model for MGLL showed suboptimal performance with MCC score 0.2 (see Figure S4). This could be explained by the low number of data points for training, bearing a total of 255 data points for the complete data set (see Table S1). Models for other targets, such as CACNA1C, CYP2C19, CYP2D6, ELANE, ESR2, PDGFRA, PTPN1 showed values modestly above 0.4 MCC.

Due to the large data set size within PDGFRB it was impractical to create SVM models, as complexity increases exponentially with large data sets. Therefore, no SVM models for PDGFRB from Bayer AG source were created.

Figures S5 and S6 display the results of outer CV results using Estate descriptors for model building. Overall, a slight decrease in the MCC values can be observed when compared to the models created by using CDDDs. Results of the outer CV in both data sources indicate for the majority of targets overall good performances reaching MCCs over 0.5. Models created using the ChEMBL domain showed lower performance for CDC25A, CYP2C8, MALT1, PDGFRA, with MCC values below 0.4. The Bayer AG models for the targets CACNA1C, CYP2C19, CYP2D6 and PTPN1 showed MCC values below 0.4. In the instance of MGLL, the MCC exhibited a value of 0.3.

Based on the performance of the models evaluated by outer CV across the 40 targets, it can be concluded that the models generalize well within their data source. To evaluate their applicability to the respective other data source, all models were retrained with the full data sets of each target from each data set source. For this, the hyperparameters that were chosen most often in the ensembles of the repeated outer CVs were selected to train the final models.

### Model Performance Across Data Sources

#### Model Performance on ChEMBL Data Sets

**Figure 3:**
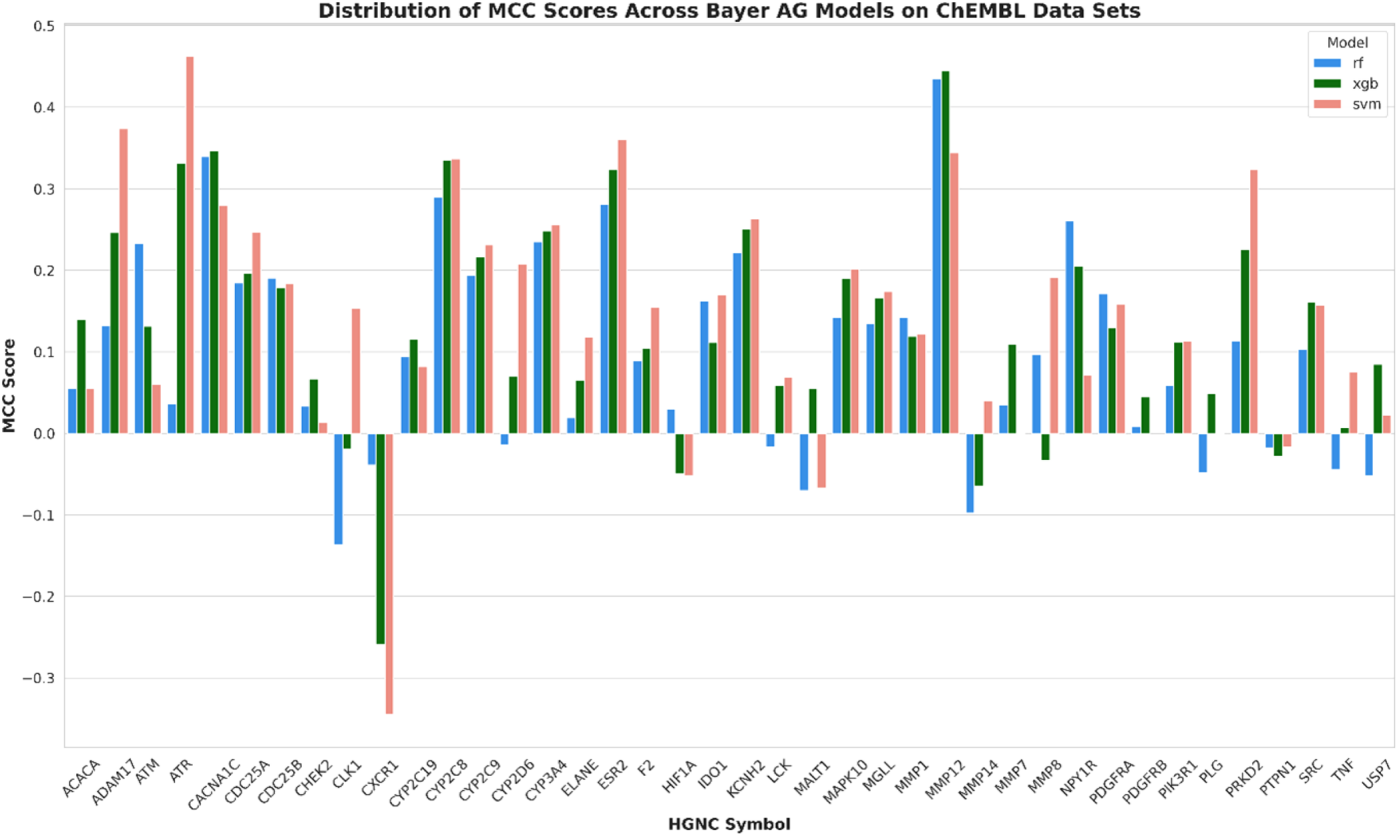
A bar chart illustrating the performance of models trained on Bayer AG data predicting ChEMBL test sets, using CDDDs to represent the chemical structures. The bar chart displays the MCC scores achieved by different ML algorithms across the 40 Targets. The *x*-axis represents 40 targets, while the *y*-axis shows the MCC score. Each bar is color coded to represent a different ML algorithm: red for SVM, green for XGB, and blue for RF.

**Figure 4:**
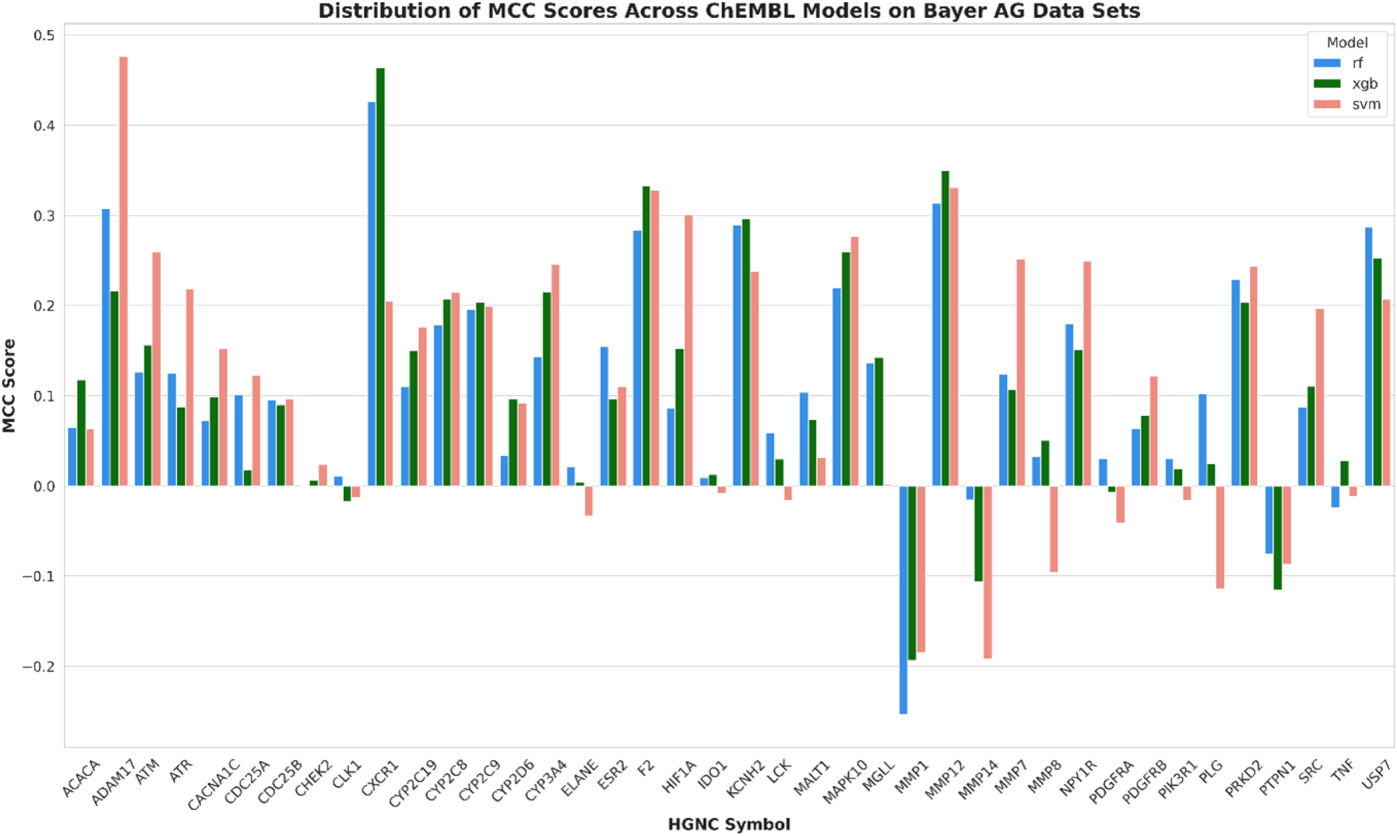
A bar chart illustrating the performance of models trained on ChEMBL data predicting Bayer AG test sets, using CDDDs to represent the chemical structures. The bar chart displays the MCC scores achieved by different ML algorithms across the 40 Targets. The *x*-axis represents 40 targets, while the *y*-axis shows the MCC score. Each bar is color coded to represent a different ML model: red for SVM, green for XGB, and blue for RF.

The *t* test calculations of the mean MCC values revealed significant differences when CDDDs and Estate descriptors were used, with CDDDs outperforming the Estate descriptors. Given the performance exhibited by models built with CDDDs compared to the Estate descriptors, the models were retrained and tested using CDDDs to leverage their efficacy in enhancing predictive accuracy. Figures 3 and 4 display the performance of the final models trained with Bayer AG data predicting the corresponding ChEMBL data set for the same target (Figure 3) and vice versa (Figure 4). Overall, it becomes evident that model performance across the data domain shows a suboptimal outcome. Models trained with the Bayer AG data set exhibited a notable deficiency in accurately predicting the corresponding ChEMBL data sets. Only ATR and MMP12 showed MCC values above 0.4. Most targets exhibited MCC values below 0.3, suggesting moderate to poor agreement between the predicted and actual classification. For targets such as CHEK2, CXCR1, HIF1A, MALT1, MMP12, MMP8, PLG and PTPN1MCC values were worse than random, falling around or below zero.

#### Model Performance on Bayer AG Internal Data Sets

A similar trend can be seen when looking at the performance of models trained on ChEMBL data predicting Bayer AG data sets (Figure 4). In this scenario, MCC exceeded 0.4 for only two of the targets, ADAM17 and CXCR1. MCC values over 0.3 were only retrieved for F2 and MMP12. Model performance for the targets, CHEK2, CLK1, ELANE, IDO1, LCK, MMP1, MMP14, MMP8, PDGFRA, PDGFRB, PIK3R1, PLG, PTPN1 and TNF is poor with MCC values around or below zero. Overall, across both data sources, no algorithm demonstrated superiority over the others. Moreover, it becomes evident that using the ChEMBL database for model training and applying these models on proprietary data sets such as Bayer AG and vice versa, using Bayer AG data sets for model building and applying them to ChEMBL, produce inadequate levels of prediction accuracy and suggest they are not suitable for applying the models across data sources.

### Visualization of Chemical Space for the 40 Targets and Mean Tanimoto Similarity Calculations of the Nearest Neighbors

The observed prediction performances of models trained on data from one source predicting data from the respective other sources were poor in most cases. As a first assessment on the proximity of chemical spaces of the two data sources, UMAP visualizations were applied. One point of interest here was the analysis to which extent UMAP visualization could indicate good or bad model applicability.

The Estate descriptors were chosen over CDDDs, as internal studies at Bayer AG show a mismatch when assessing the structural similarity of compounds represented as CDDDs based on the Tanimoto Coefficient. The resulting 40 UMAP visualizations indicated similarity between the compounds in terms of drug alikeness to some extent. This can be explained by the nature of the Estate molecular descriptors, as they are designed to capture important molecular features related to drug-likeness, such as hydrogen bond donors and acceptors, hydrophobicity, size and complexity. Therefore, ML models built on Estates descriptors following the same procedure as described above for CDDD descriptors were trained for all 40 targets. These were used to reflect on how indicative overlap of chemical space is to assess the applicability of models across data sources.

The analyses indicated that representatives of the Bayer AG database can be found in nearby areas or overlapping with ChEMBL database representatives. For illustrative purposes, three targets have been selected, ADAM17, CLK1 and CXCR1 to reflect on how UMAP visualizations may explain model performances.

**Figure 5:**
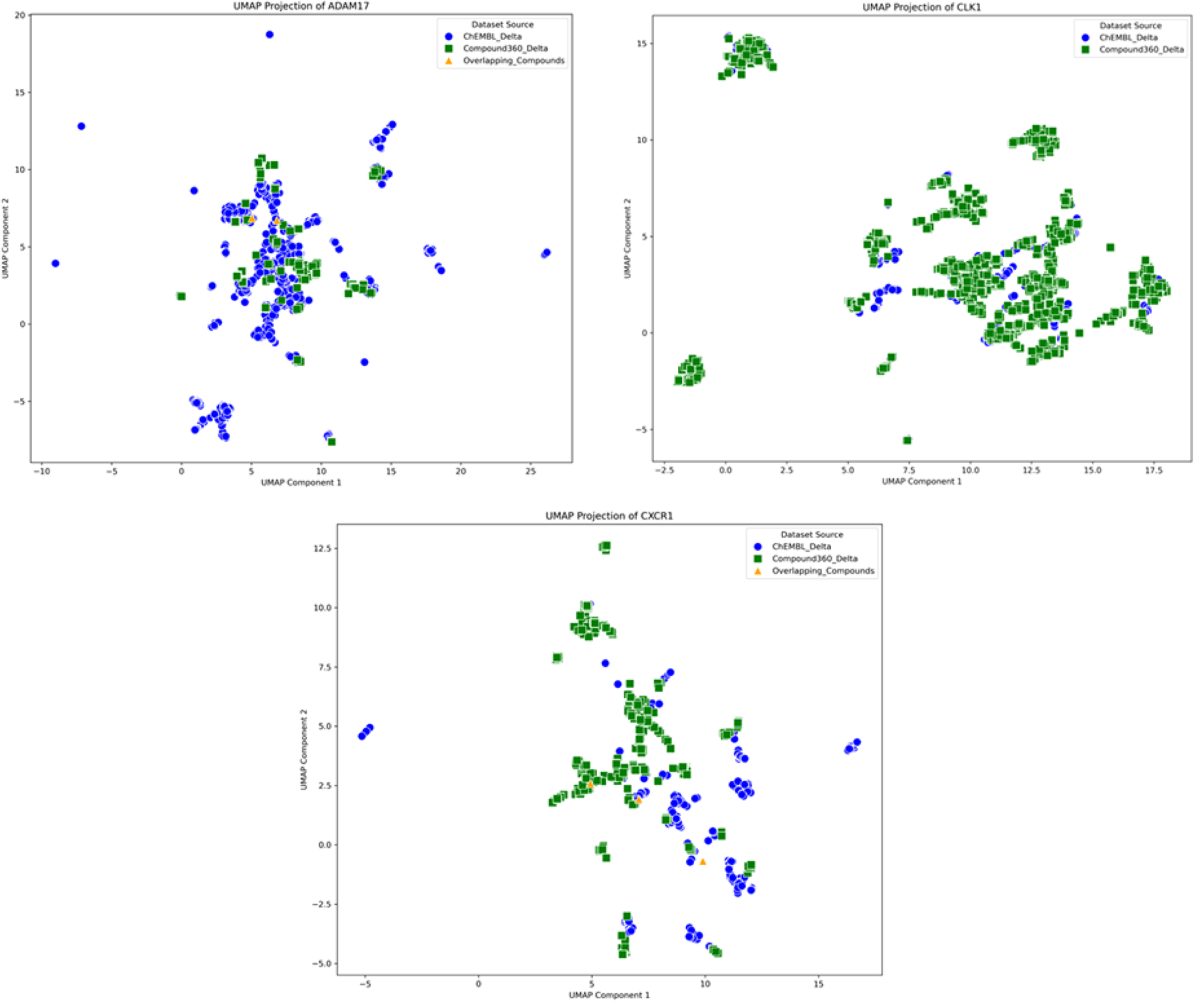
UMAP visualizations of Bayer AG and ChEMBL data sets for ADAM 17, CLK1 and CXCR1. Different shapes and colors in the figures indicate the unique and overlapping compounds from both data sources Overlapping compounds are indicated as yellow triangles. Blue dots represent the ChEMBL chemical space whereas green squares represent Bayer AG chemical space.

Looking at the UMAP visualization for ADAM17 one can observe that the chemical space covered by the Bayer AG compounds is well embedded in the chemical space covered by the ChEMBL compounds. Following this observation, a good model performance when the ChEMBL trained model is applied to Bayer AG compounds can be expected. In line with that, this experiment yields a MCC value of 0.56 for the RF model (Figure S12). When the Bayer AG data source trained model is applied to compounds from the corresponding ChEMBL data set, a drop of performance, 0.30 MCC for the RF model (Figure S11), can be observed. This can be explained by the high amount of ChEMBL compounds in a chemical space beyond that of the Bayer AG compounds used for model training (see Figure 5).

Interestingly, this does not apply for CLK1. When models for CLK1 are applied across data sources, both models fail to generalize and show MCC values between MCC–0.25 when Bayer models are applied on ChEMBL and MCC values of −0.21 when applied vice versa (Figures S11 and S12). However, looking at the UMAP visualizations for CLK1, most of the chemical space covered by ChEMBL compounds are embedded in the chemical space covered by the Bayer AG compounds, but covers distinct areas. Hence, one would expect a decent performance of the model trained on Bayer data when predicting ChEMBL compounds based on the UMAP visualization (Figure 5). Observed model performance shows, that UMAP visualization does not cover all information needed to understand model applicability in this case.

The UMAP visualization for CXCR1 shows larger clusters of nonrelated chemical spaces for the two different data sources. From this, low transferability of the model trained on one data source to predict the respective other one can be expected. The ChEMBL model trained for CXCR1 shows a performance of 0.28 MCC for the XGB model predicting Bayer AG compounds. Even worse model performance can be seen when the Bayer AG model is applied to ChEMBL compounds (MCC = −0.49, Figure S11).

Unfortunately, across all 40 targets the UMAP visualizations are not informative in order to assess the transferability of models trained on one data source to predict compounds from the respective other data source.

**Figure 6:**
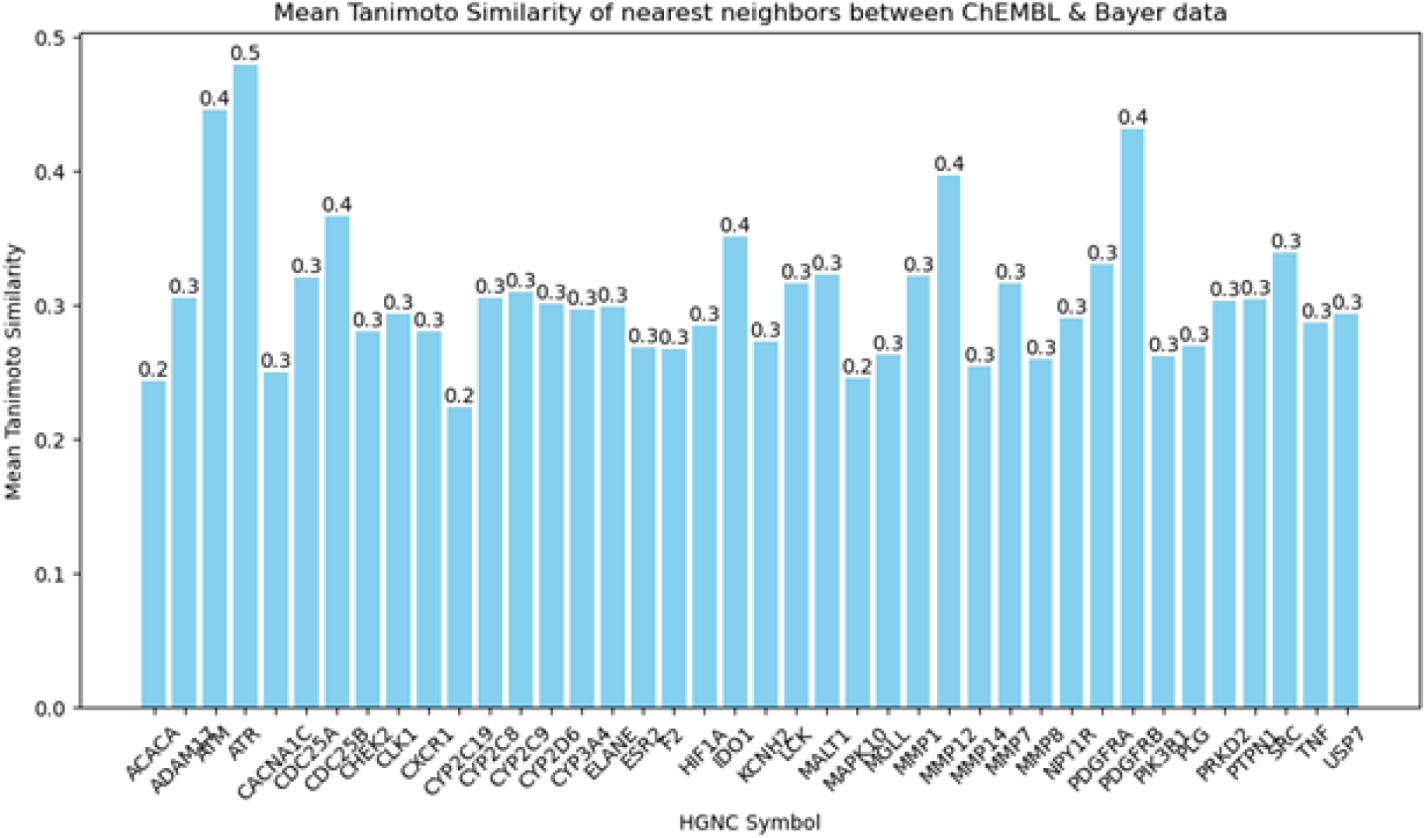
A bar chart illustrating the outcome of mean Tanimoto similarity (based on Morgan fingerprints 2) of the nearest neighbors between ChEMBL and Bayer AG data. The *x*-axis represents all 40 targets, while the *y*-axis depicts the mean Tanimoto similarity of the nearest neighbors.

In addition, we calculated mean Tanimoto similarities of the nearest neighbors based on Morgan fingerprints 2 to assess similarities between corresponding data sets from the two data sources for all 40 targets. Figure 6 shows the results of this analysis. The results of the mean Tanimoto similarity of the nearest neighbors revealed that the data sets in general exhibit low chemical similarity. Figure 6 demonstrates that for the majority of targets a similarity of 0.3 was obtained for the nearest neighbors of the two data sources, suggesting differences in the chemical space. Only for the target Serine/Threonine-protein kinase ATR, a mean Tanimoto similarity of 0.5 was identified to the nearest neighbors (Figure 6). Despite this, the ML models for this target achieved poor MCC values across the data set sources (0.46 and 0.22, respectively; Figures 3 and 4). The lowest mean Tanimoto similarity to the nearest neighbor was retrieved for Acetyl-CoA carboxylase 1 (ACACA), Cytochrome P450 2C19 (CYP2C19) and Mitogen-activated protein kinase 10 (MAPK10), with a value of 0.2 (Figure 6). For example, when ML models from Bayer AG for CYP2C19 and MAPK10 are tested on ChEMBL data, MCC values of 0.12 for the XGB model and 0.20 for the SVM model were obtained, while the vice versa setting yielded MCC values of 0.18 and 0.28 for the SVM models (Figures 3 and 4). ML models for Interleukin 8 receptor, alpha (CXCR1), which showed mean Tanimoto similarities of 0.3, exhibited good MCC scores of 0.46 when ChEMBL models were applied to Bayer AG. However, when models were applied vice versa, MCC values of −0.34 were observed, indicating significant decrease in performance (Figures 3 and 4).

Comparing these observations on the data set similarities to the ML performance, it becomes evident that this approach does not align with the performance metrics. These findings suggests that Tanimoto similarities of the nearest neighbor may not reflect the utility in assessing the transferability of models to predict compounds from the other data source.

### Distribution of Key Physicochemical Properties Across All 40 Off-Targets

The analysis of the distribution of key physicochemical properties across all 40 off-targets between the two respective data sets (Bayer AG and ChEMBL), including Molecular Weight, predicted logD at pH 7.5, Topological Polar Surface Area, Heavy Atom Count, Rotatable Bond Count, H-Bond Donor Count, and H-Bond Acceptor Count, revealed differences between the two data sources across nearly all data sets. Among these, the predicted logD at pH 7.5 exhibited the smallest variation, while the Heavy Atom Count showed the largest differences. For example, PIK3R1, MMP8, CLK1, and ACACA with details available on Zenodo (https://zenodo.org/doi/10.5281/zenodo.14355717). The majority of compounds within the Bayer AG data sets demonstrated a higher Molecular Weight compared to their corresponding ChEMBL data sets. However, the analysis of Rotatable Bond Count, H-Bond Donor Count, H-Bond Acceptor Count, and Topological Polar Surface Area showed no consistent trends. Some data sets displayed higher or lower values for these properties, while others showed negligible differences. Overall, the results highlight notable variability in physicochemical properties across the data sources, alongside differences in structural information identified through the Mean Tanimoto Similarity analysis. All results can be found on Zenodo (https://zenodo.org/doi/10.5281/zenodo.14355717).

### Mixed Models

Applying ML models trained on data from only one data source to predict bioactivities of the other data source did not show satisfying performance and the analysis of the covered chemical space did not turn out to be informative on the applicability. Hence, the training of ML models on data sets combining the two sources was investigated.

**Figure 7:**
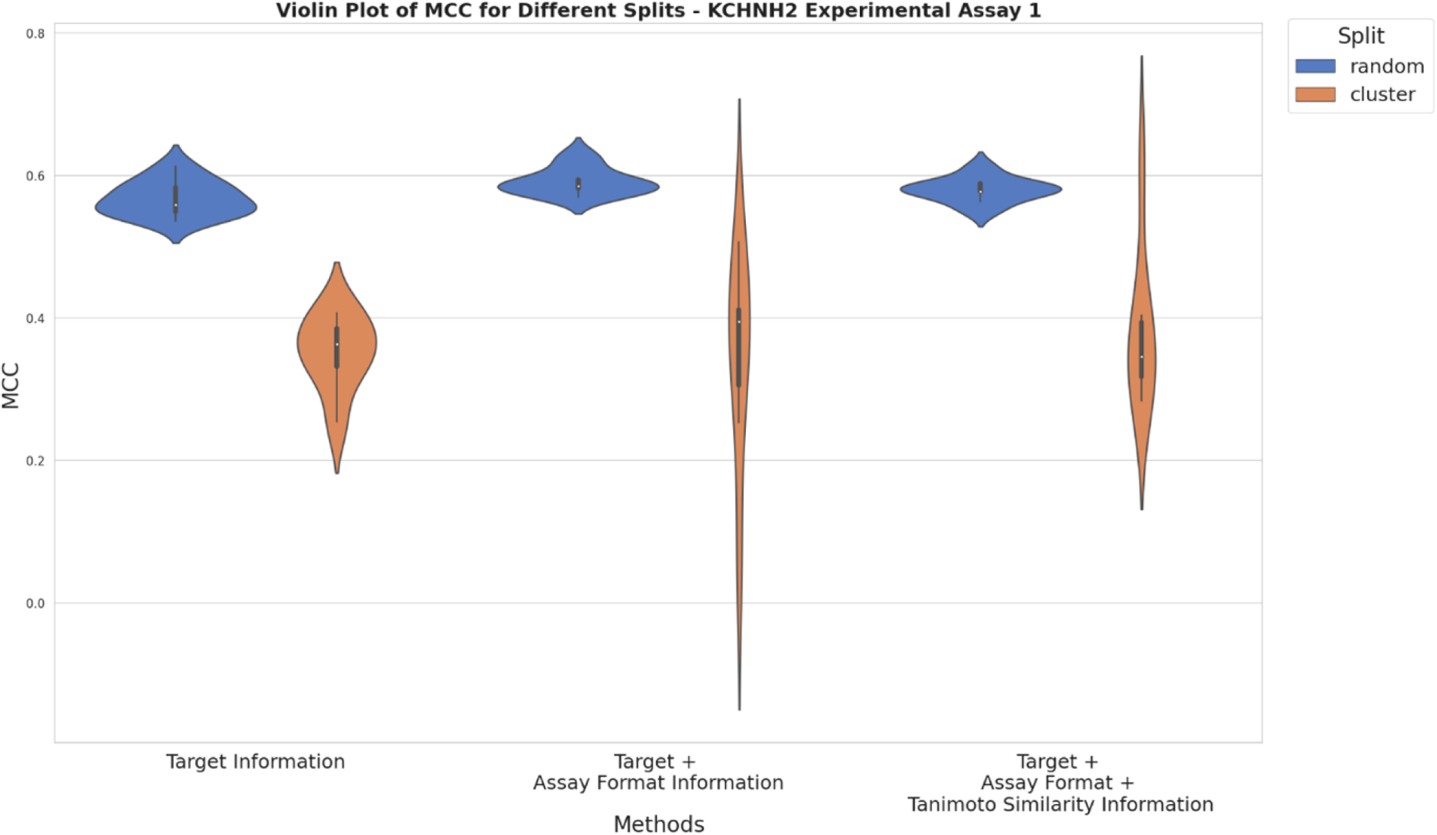
Violin plots illustrating results from random and cluster-based nested CV for KCNH2 experimental setup 1 from Bayer AG. The *x*-axis represents different approaches used for compiling training data based on adding ChEMBL data using solely target information (approach a), additionally assay format information (approach b) and additionally Tanimoto similarity ≥ 0.230 (approach c). *Y*-axis depicts the mean MCC values obtained from random and cluster-based nested CV.

**Figure 8:**
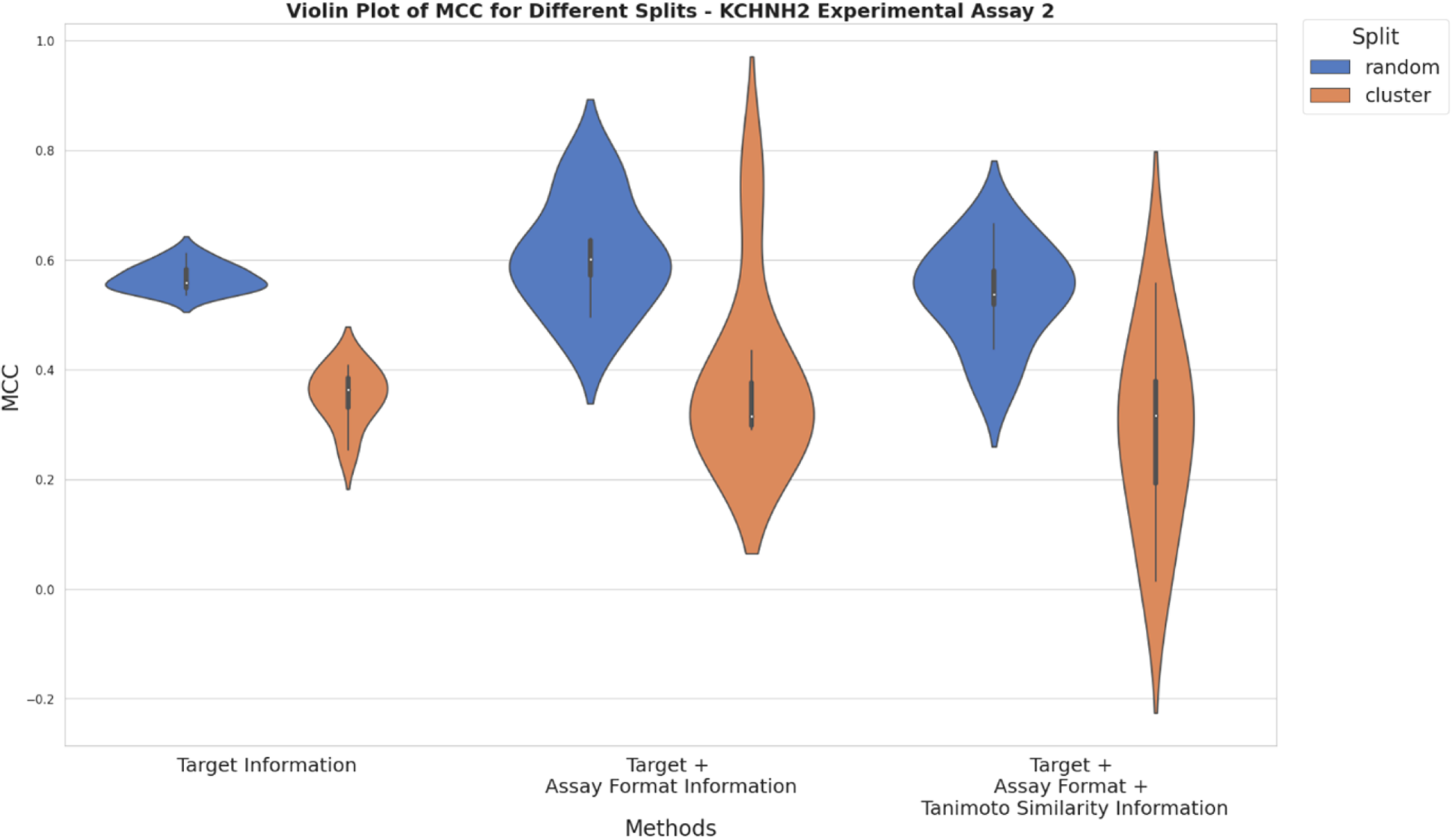
Violin plots illustrating results from random and cluster-based nested CV for KCNH2 experimental setup 2 from Bayer AG. The *x*-axis represents different approaches used for compiling training data based on adding ChEMBL data using solely target information (approach a), additionally assay format information (approach b) and additionally Tanimoto similarity ≥ 0.230 (approach c). *Y*-axis depicts the mean MCC values obtained from random and cluster-based nested CV.

**Figure 9:**
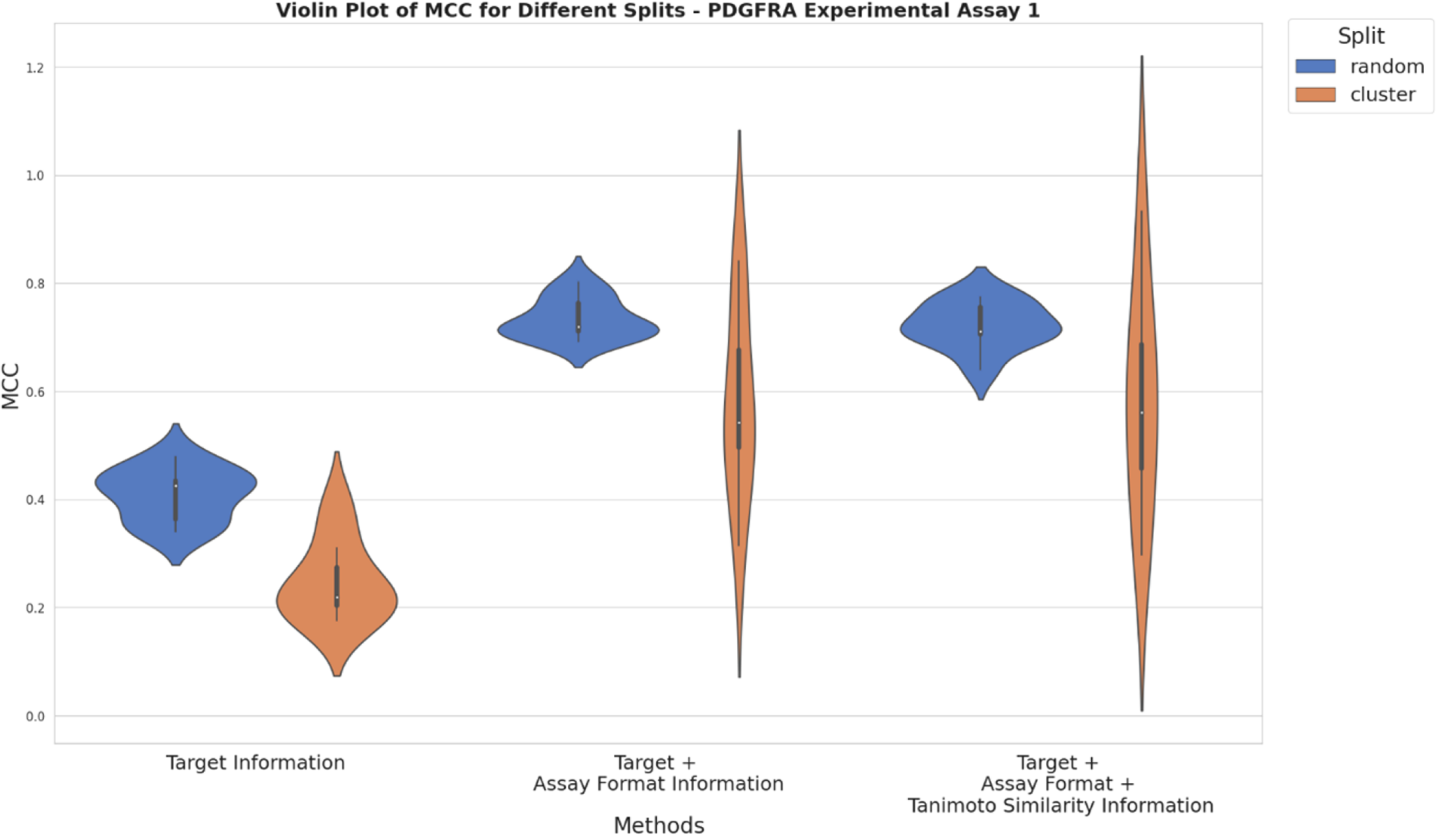
Violin plots illustrating results from random and cluster-based nested CV for PDGFRA experimental setup 1 from Bayer AG. The *x*-axis represents different approaches used for compiling training data based on adding ChEMBL data using solely target information (approach a), additionally assay format information (approach b) and additionally Tanimoto similarity ≥ 0.230 (approach c). *Y*-axis depicts the mean MCC values obtained from random and cluster-based nested CV.

**Figure 10:**
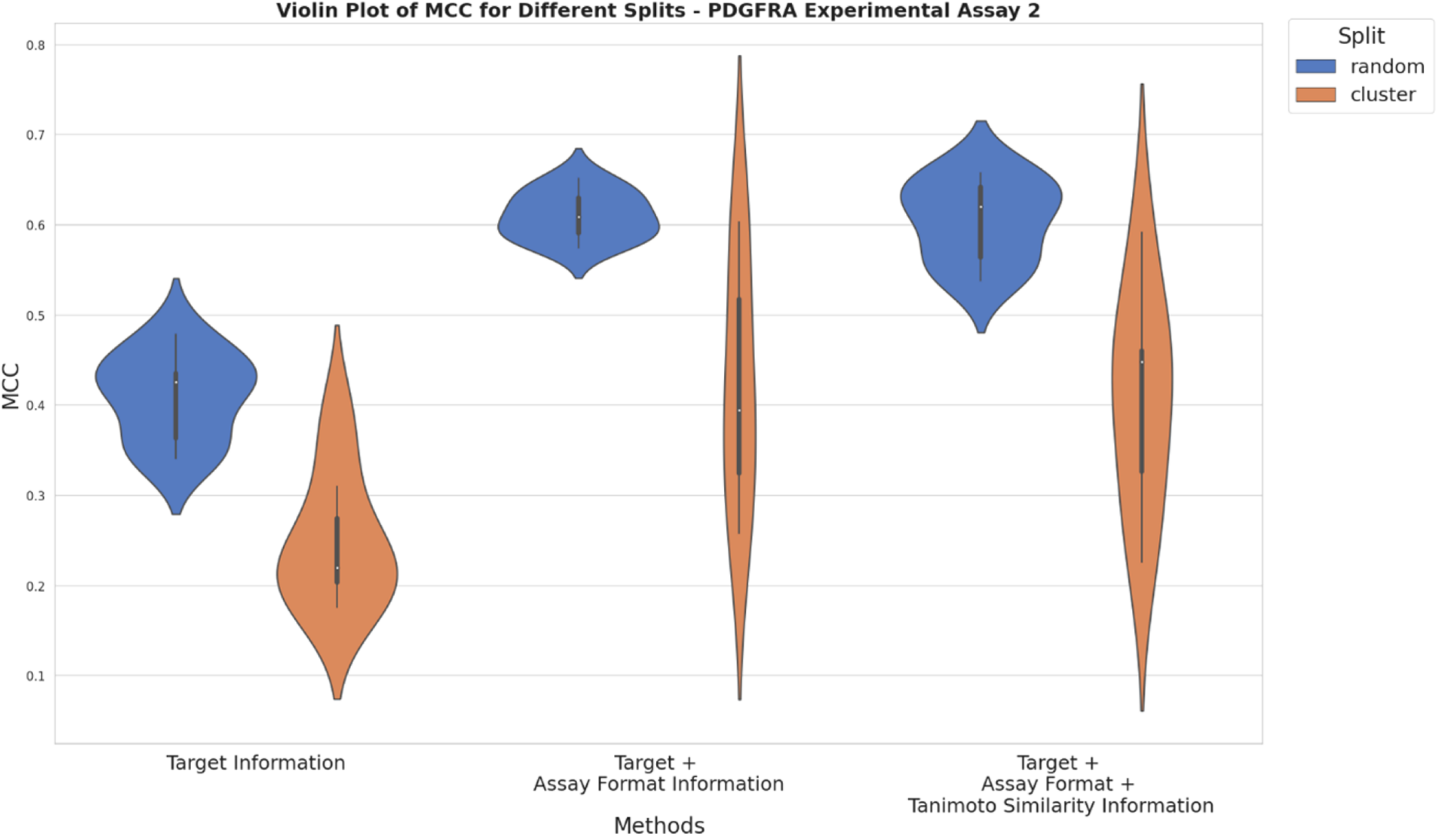
Violin plots illustrating results from random and cluster-based nested CV for PDGFRA experimental setup 2 from Bayer AG. The *x*-axis represents different approaches used for compiling training data based on adding ChEMBL data using solely target information (approach a), additionally assay format information (approach b) and additionally Tanimoto similarity ≥ 0.230 (approach c). *Y*-axis depicts the mean MCC values obtained from random and cluster-based nested CV.

**Figure 11:**
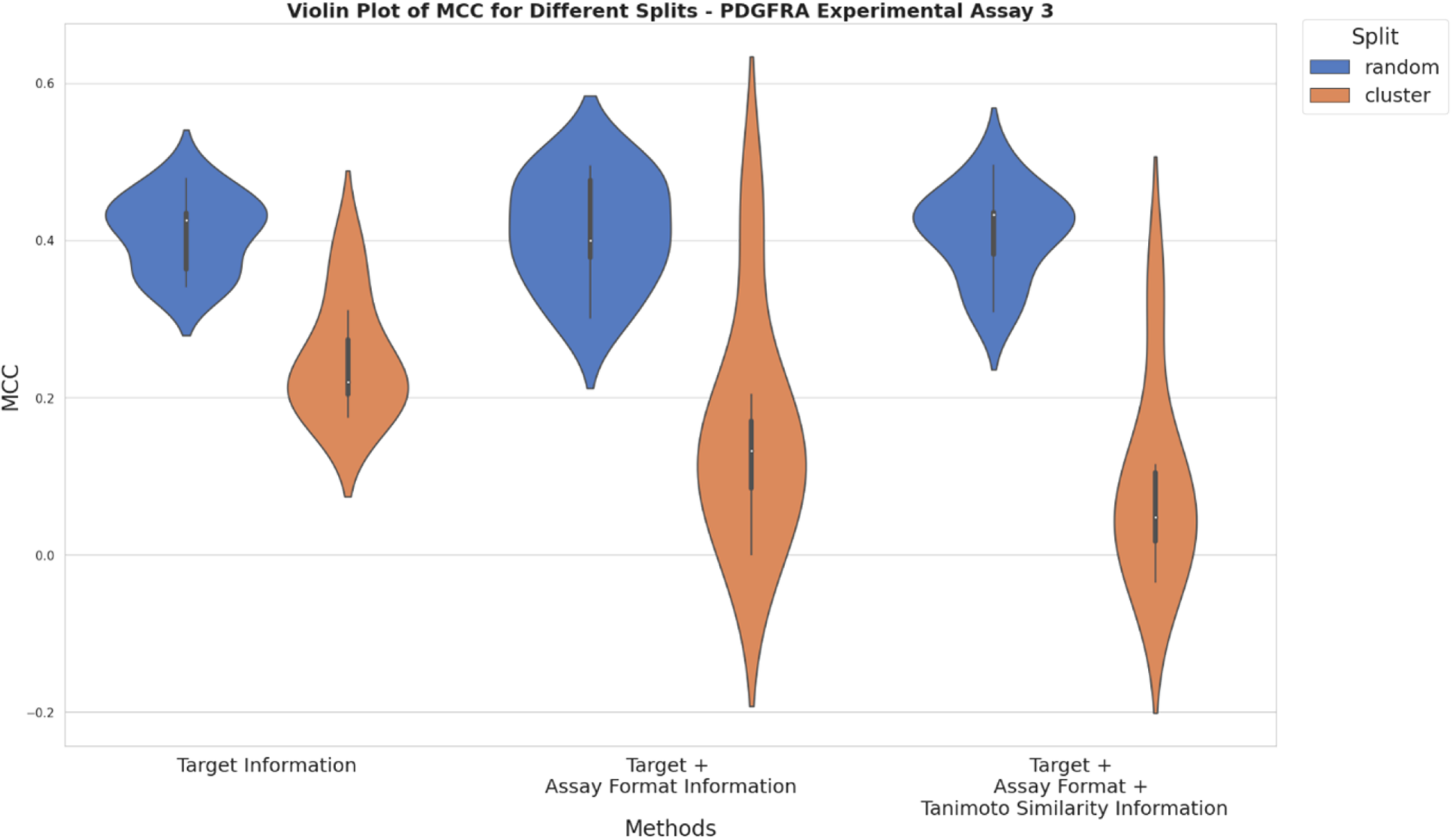
Violin plots illustrating results from random and cluster-based nested CV for PDGFRA experimental setup 3 from Bayer AG. The *x*-axis represents different approaches used for compiling training data based on adding ChEMBL data using solely target information (approach a), additionally assay format information (approach b) and additionally Tanimoto similarity ≥ 0.230 (approach c). *Y*-axis depicts the mean MCC values obtained from random and cluster-based nested CV.

Figures 7–11 show the model performance for the three different approaches (a to c) used for the compilation of mixed training data sets as described in the methods section above.

Figures 7 and 8 visualize the performance of models trained with the three differently compiled training data sets, assessed by random and cluster-based nested CV for KCNH2. Interestingly, performance increased slightly for random and cluster-based splits when taking assay format information into account when compiling mixed data sets for model training, following approach b) (Figure 7). However, when in addition chemical similarity was taken into account (approach c), no further improvement in model performance can be observed. The slightly worse performance and higher variability of MCC values obtained from cluster-based splits in comparison to random-based splits can be explained by cluster-based test splits being relatively dissimilar to the respective training splits while in random-based splitting, similarity is homogeneously distributed across splits, resulting in better transferability of predictions from training to test splits. Additionally, training and test set size as well as split size influence predictive performance and variability.

When analyzing the cluster-based nested CV results, a high frequency of MCC values around 0.38 can be observed when only individual target information is used. A wide range of MCC values is evident when additionally, assay format information is included, with an even broader range observed when Tanimoto similarities are added. This shows the impact of split size and split heterogeneity on model performance is also depending on total data set size, since the more restrictive approaches of data set compilation generally lead to a reduction in data set size compared to the less restrictive approaches.

For two of the PDGFRA experimental setups from Bayer AG (Figures 9 and 10), compiling the training data according to approach b) showed improved performance, with MCC values ranging between 0.6 and 0.75 for random and 0.4 and 0.58 for cluster-based nested CV compared to approach a). In contrast, when utilizing the third experimental setup from Bayer AG no difference in performance was observed for the three different approaches of training data compilation (Figure 11). This resulted in MCC values of approximately 0.45 for random-based nested CV and 0.2 for cluster-based approaches.

## Discussion

The first part of the study was to confirm on a larger scale the results obtained from Smajić et al. that models trained on public sources are not applicable for in-house predictions, and vice versa. If so, the second focus was the investigation of different strategies for enhancing in-house data sets with selected public data, considering both chemical space and experimental setup information. Figures S1 and S2 show that the two data sources, ChEMBL and Bayer AG, exhibit inherent differences in their core characteristics, as one data source is skewed toward actives while the other shows imbalance toward inactives. Furthermore, from the confusion matrices which can be found on Zenodo (https://zenodo.org/doi/10.5281/zenodo.14355717) it becomes evident that for the majority of targets, the models trained on ChEMBL data overpredict positives and models trained on Bayer AG data overpredict negatives. This corroborates the earlier study by Smajić et al.

The assessment of the applicability of models trained on one data source to predict data from the other data source was investigated using the dimensionality reduction method UMAP, calculating key physicochemical properties and assessing structural similarity through mean Tanimoto similarity. In addition to addressing the main research questions within this manuscript, these methods provided a comprehensive view of the chemical space covered by the Bayer AG and ChEMBL data sets. Low Mean Tanimoto Similarity values indicate reduced structural similarity between the two data set domains. However, our study shows that these approaches do not provide insights into whether ML models will fail when tested across different data sources. For certain targets, an overlap in chemical space was observed, but this was not consistently reflected in model performance, and vice versa. The two different data sources share very few identical compounds with the highest percentage of identical compounds for one target being 10% for the target ATR.

Besides focusing on the utility of the applicability domain estimation we also were interested in the performances of different combinations of descriptors and ML algorithms. Across the majority of target end points, RF, XGB and SVM algorithms applied in the nested CV showed better performances when using CDDDs compared to Estate descriptors, irrespective of the data source. Interestingly, combining SVM with CDDDs enhanced performance compared to using RF and XGB with CDDDs, albeit this effect is not statistically significant. This can be observed in Figures S3 and S4. For a better depiction of the overall performances between the three ML algorithms and descriptors for all 40 targets, violin bar plots have been created. The violin plots can be found in the Supporting Information. Figures S7–S10 show the overall mean MCC (aggregating the repeated outer CV results) for all 40 targets when RF, XGB, and SVM models are used in combination with CDDDs and Estate descriptors. A high frequency of the overall MCC values for SVM models in combination with CDDDs can be seen around values higher than 0.6. In addition, the median sits slightly higher than the one of RF and XGB models with a MCC value above 0.6 indicating overall good performance in terms of classification accuracy. A drop in performance, from 0.6 to 0.5 MCC, can be observed when applying Estate descriptors to build SVM models.

In order to investigate the predictivity of mixed ML models trained on data sets compiled from both data sources, three different approaches were utilized: Starting with data from one experimental setup from Bayer AG, ChEMBL data were added to the training set based either on only target information (approach a), or on target information and assay format information (approach b) or on target information and assay format information and Tanimoto similarity (approach c). For the analyzed targets KCNH2 and PDGFRA, no significant advantage was evident for approach c) versus approach b). Nevertheless, better performances for certain target + assay format combinations (approach b) were retrieved compared to combining data sets according to only target information (approach a), suggesting its potential utility in the training data compilation process. However, given the lack of detailed annotations in the ChEMBL database, caution is warranted regarding potential biases and uncertainties inherent in combining data sets, which could introduce variability in the analysis outcomes. Also, applying more restrictive criteria for data set compilation leads to smaller training data set sizes. Here, finding the balance between scientifically sound combination approaches and adequate data set size remains a challenge. Therefore, additional analysis, and experiments are necessary to explore this further, particularly utilizing qualitatively annotated data for a more in-depth understanding. Furthermore, as demonstrated in the recent Tox24 challenge, representation learning methods^28^ as well as multitask learning approaches have the potential to further enhance model performance, which could be explored further in future studies.

## Conclusions

In this study, we present significant differences between the different data sources of publicly available vs proprietary data and their impact on the ML model performances when applied to data sets out of the training set source. An evaluation of UMAP visualizations and Tanimoto similarity calculations for preassessing model applicability on data sets other than its training data set sources reveal poor guidance. Additionally, the exploration of various approaches to combine data sets from different sources based on the inclusion of experimental assay information is presented. Here we may see that considering assay format information improves predictive quality in some cases while taking chemical similarity into account seems to play a minor role. In addition, we visualize chemical spaces for all 40 investigated targets and highlight their different distribution/location in chemical space and differences in chemical similarity using mean Tanimoto similarity calculations of the nearest neighbor. We demonstrate that models trained on data from one source are not suitable for predicting compounds from other data sources, which is most probably due to lack of overlap in their chemical spaces. If it is not possible to obtain decent model performance on in-house data only, results obtained with KCNH2 and PDGFRA indicate that enhancing the training data with public compounds—considering experimental setup information such as assay format in addition to target information—can enhance model performance. However, leveraging public data is not straightforward due to limited annotations, making it difficult to obtain high-quality data, even though it holds the potential for improved models. This strengthens the importance of defined meta data annotations in both public and proprietary data sets which are unfortunately currently often lacking. Moreover, this study contributes to various domains within ML approaches. By highlighting the differences in chemical space across domains, it seeks to raise awareness within the community about the potential challenges associated with using chemical and biological data from diverse sources for model training.

## Supporting Information Available

Data availability for both data set domains, distribution of actives and inactives, bar charts illustrating the results of nested CV (CDDDs + Estates), overall MCC distribution from random-based nested CV using CDDD and estate descriptors, and visualization of chemical space for 40 targets (PDF)

## Abbreviations

ML: machine learning
AI: artificial intelligence
QSAR: quantitative structure–activity relationship
CDDD: continuous data driven descriptor
Estate: electrotopological state
RF: random forest
XGB: xgboost
SVM: support vector machine
UMAP: uniform manifold approximation and projection for dimension reduction
AC: accession number
InChIs: IUPAC international chemical identifiers
SMILES: simplified molecular input entry specification
B: binding
A: ADME
T: toxicity
KCNH2: potassium voltage-gated channel subfamily H member 2
PDGFRA: platelet-derived growth factor receptor A
BA: balanced accuracy
ACC: accuracy
PRC: precision
Sen: sensitivity
Spec: specificity
AUC: area under the curve
MCC: Matthews correlation coefficient
TP: true positive
FP: false positive
FN: false negative
CV: cross-validation
ATR: serine/threonine-protein kinase ATR
CDC25A: M-phase inducer phosphatase 1
CYP2C19: cytochrome P450 2C19
CYP2C8: cytochrome P450 2C8
CYP2C9: cytochrome P450 family 2 subfamily C member 9
MALT1: mucosa-associated lymphoid tissue lymphoma translocation protein 1
MGLL: monoacylglycerol lipase
CACNA1C: calcium channel, voltage-dependent, L type, alpha 1C subunit
CYP2D6: cytochrome P450 2D6
ELANE: neutrophil elastase
ESR2: estrogen receptor beta
PDGFRA: platelet-derived growth factor receptor A
PTPN1: tyrosine-protein phosphatase nonreceptor type 1
PGFRB: platelet-derived growth factor receptor beta
MMP12: matrix metalloproteinase-12
CHEK2: checkpoint kinase 2
CXCRR: interleukin 8 receptor, alpha
HIF1A: hypoxia-inducible factor 1-alpha
MMP8: matrix metalloproteinase-8
PLG: plasminogen
ADAM 17: metalloprotease 17
F2: coagulation Factor II
CLK1: dual specificity protein kinase CLK1
IDO1: indoleamine-pyrrole 2,3-dioxygenase
LCK: lymphocyte-specific protein tyrosine kinase
MMP1: matrix metalloproteinase-1
MMP14: matrix metalloproteinase-14
PIK3R1: phosphatidylinositol 3-kinase
TNF: tumor necrosis factor
ACACA: acetyl-CoA carboxylase 1
MAPK10: mitogen-activated protein kinase 10
ATM: ATM serine/threonine kinase
CDC25B: M-phase inducer phosphatase 2
CYP3A4: cytochrome P450 3A4
MMP7: matrix metalloproteinase-7
NPY1R: neuropeptide Y receptor type 1
PRKD2: serine/threonine-protein kinase D2
SRC: proto-oncogene tyrosine-protein kinase Src
USP7: ubiquitin specific protease
