# Supplementary material for: Data Exploration for Target Predictions Using Proprietary and Publicly Available Data Sets
Source: Chem Res Toxicol. 2025 Apr 20;38(5):820–33. doi: 10.1021/acs.chemrestox.4c00347 (PMC12093362; doi:10.1021/acs.chemrestox.4c00347)
Supplement: Supplementary file 1 — tx4c00347_si_001.pdf [file tx4c00347_si_001.pdf]

Supporting Information for Publication: “Data Exploration for Target Predictions using Proprietary & Publicly Available Data Sets”

Aljoša Smajić[1], Thomas Steger-Hartmann[2], Gerhard. F. Ecker[1], Anke Hackl[2*],

[1]University of Vienna, Department of Pharmaceutical Sciences, 1090 Vienna, Austria

[2]Bayer AG, Pharmaceuticals Division, 13353 Berlin, Germany

Table of Contents

Table of Contents

Data Availability for both Data Set Domains 2

Distribution of Actives and Inactives 3

Bar Charts illustrating the Results of nested CV 4

Overall MCC Distribution from Random-based nested CV using CDDD and Estate Descriptors 7

Visualization of Chemical Space for 40 Targets 11

Data Availability for both Data Set Domains

		Entries from 40 Targets in ChEMBL				Entries from 40 Targets in Bayer AG			
HGNC Symbols	Uniprot Accession Numbers	Total	Active	Inactive	% Minority Class	Total	Active	Inactive	% Minority Class
ACACA	Q13085	449	337	112	24,94	871	391	480	44,89
ADAM17	P78536	1852	1303	549	29,64	301	53	248	17,61
ATM	Q13315	360	233	127	35,28	4037	2201	1836	45,48
ATR	Q13535	272	138	134	49,26	12969	2327	10642	17,94
CACNA1C	Q13936	281	93	188	33,1	437	207	230	47,37
CDC25A	P30304	272	131	141	48,16	464	182	282	39,22
CDC25B	P30305	528	283	245	46,4	2706	1445	1261	46,6
CHEK2	O96017	1167	959	208	17,82	567	277	290	48,85
CLK1	P49759	466	327	139	29,83	1815	772	1043	42,53
CXCR1	P25024	303	230	73	24,09	389	207	182	46,79
CYP2C19	P33261	2956	940	2016	31,8	296	157	139	46,96
CYP2C8	P10632	734	243	491	33,11	12310	6871	5439	44,18
CYP2C9	P11712	4653	1774	2879	38,13	12543	6477	6066	48,36
CYP2D6	P10635	5290	1731	3559	32,72	12558	2518	10040	20,05
CYP3A4	P08684	6866	2876	3990	41,89	12919	6945	5974	46,24
ELANE	P08246	2394	1963	431	18	535	261	274	48,79
ESR2	Q92731	1611	1178	433	26,88	786	215	571	27,35
F2	P00734	5857	3931	1926	32,88	386	68	318	17,62
HIF1A	Q16665	444	223	221	49,77	2953	2500	453	15,34
IDO1	P14902	1715	1129	586	34,17	13749	3917	9832	28,49
KCNH2	Q12809	10774	5370	5404	49,84	5235	1731	3504	33,07
LCK	P06239	2633	2085	548	20,81	2435	426	2009	17,49
MALT1	Q9UDY8	268	160	108	40,3	373	57	316	15,28
MAPK10	P53779	1418	927	491	34,63	600	310	290	48,33
MGLL	Q99685	862	605	257	29,81	255	130	125	49,02
MMP1	P03956	3198	2198	1000	31.27	700	145	555	20,71
MMP12	P39900	1390	1042	348	25,04	2195	1594	601	27,38
MMP14	P50281	976	635	341	34,94	484	221	263	45,66
MMP7	P09237	591	440	151	25,55	586	105	481	17,92
MMP8	P22894	1057	798	259	24,5	686	455	231	33,67
NPY1R	P25929	916	695	221	24,13	784	152	632	19,39
PDGFRA	P16234	924	776	148	16,02	4665	2658	2007	43,02
PDGFRB	P09619	1582	1149	433	27,37	56314	18918	37396	33,59
PIK3R1	P27986	261	138	123	47,13	1152	586	566	49,13
PLG	P00747	1307	643	664	49,2	425	115	310	27,06
PRKD2	Q9BZL6	776	392	384	49,48	3657	595	3062	16,27
PTPN1	P18031	3205	1594	1611	49,73	2483	425	2058	17,12
SRC	P12931	3734	2641	1093	29,27	5305	2269	3036	42,77
TNF	P01375	567	245	322	43,21	1890	724	1166	38,31
USP7	Q93009	256	177	79	30,86	393	176	217	44,78

Table S1: Data available for both domains: ChEMBL and Bayer AG, using a classification threshold of 10µM.

Distribution of Actives and Inactives

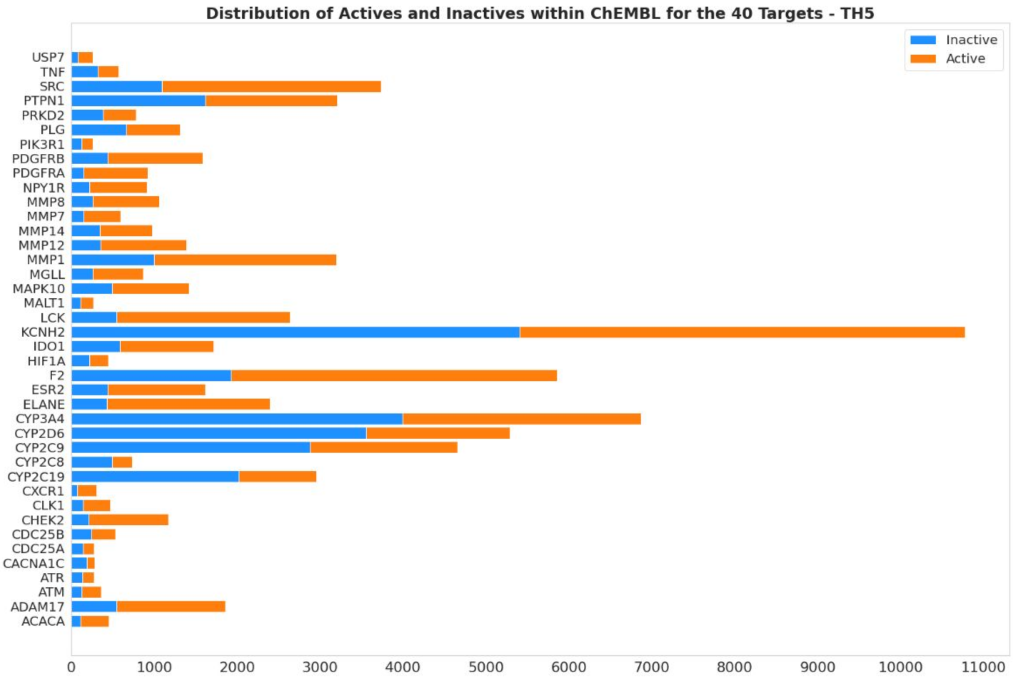

Figure S1: Overview of the distribution between actives and inactives (pChEMBL = 5), as well as the corresponding HGNC symbols. The x-axis shows the number of entries per target in the ChEMBL database. The y-axis lists the HGNC symbols of the 40 targets. Blue color indicates inactive entries. Orange color indicates active entries.

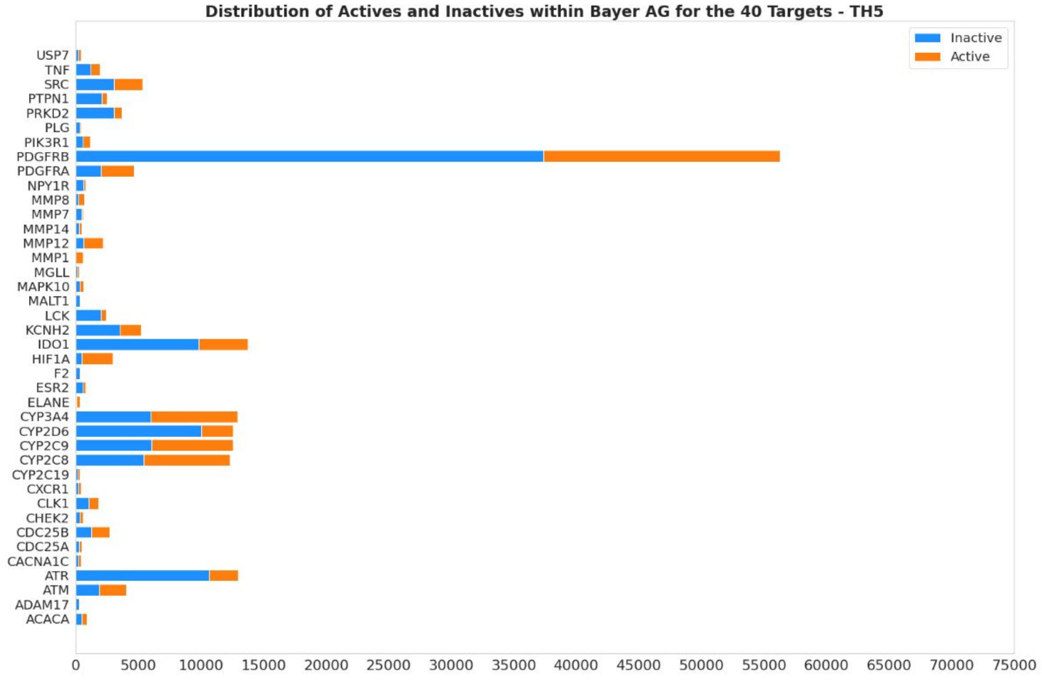

Figure S2: Overview of the distribution between actives and inactives (pChEMBL = 5), as well as the corresponding HGNC symbols. The x-axis shows the number of entries per target in the Bayer AG database. The y-axis lists the HGNC symbols of the 40 targets. Blue color indicates inactive entries. Orange color indicates active entries.

Bar Charts illustrating the Results of nested CV

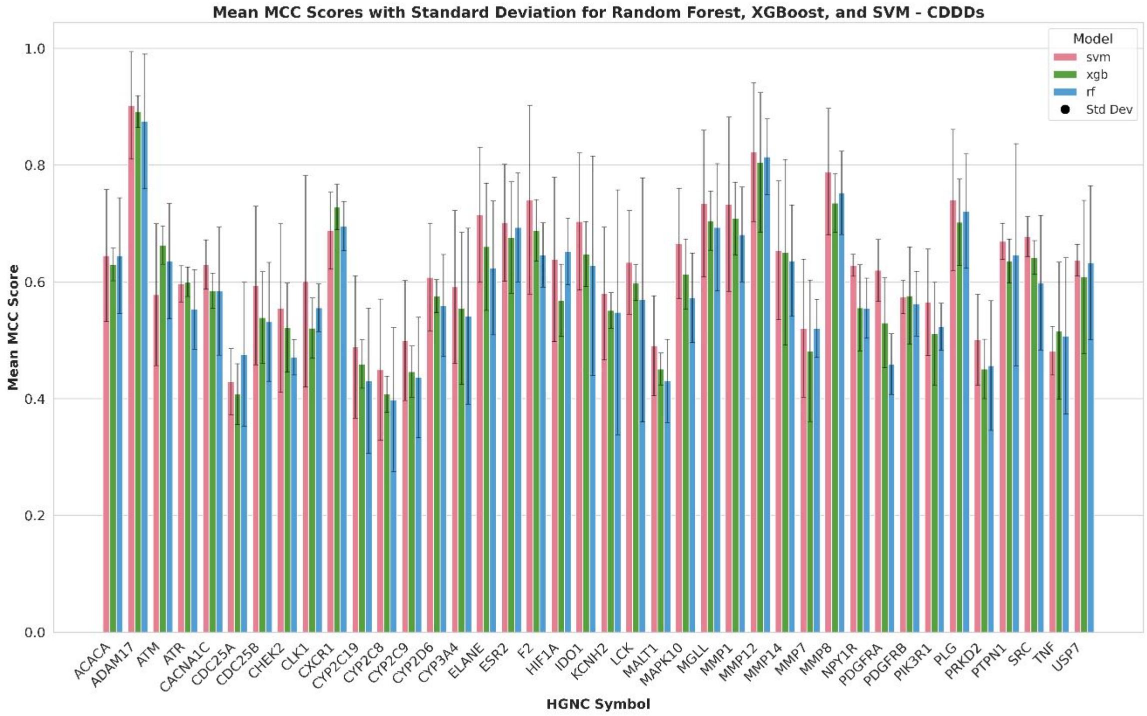

Figure S3: A bar chart illustrating the results of nested CV using ChEMBL data sets with CDDDs in combination with 3 different ML algorithms. The bar chart displays the mean MCC scores achieved by different ML models across the 40 Targets. The x-axis represents 40 targets, while the y-axis shows the mean MCC score. Each bar is color coded to represent a different ML algorithm: red for SVM, green for XGB, and blue for RF. Error bars, representing standard deviations, are displayed on each bar, indicating the variability of the MCC scores.

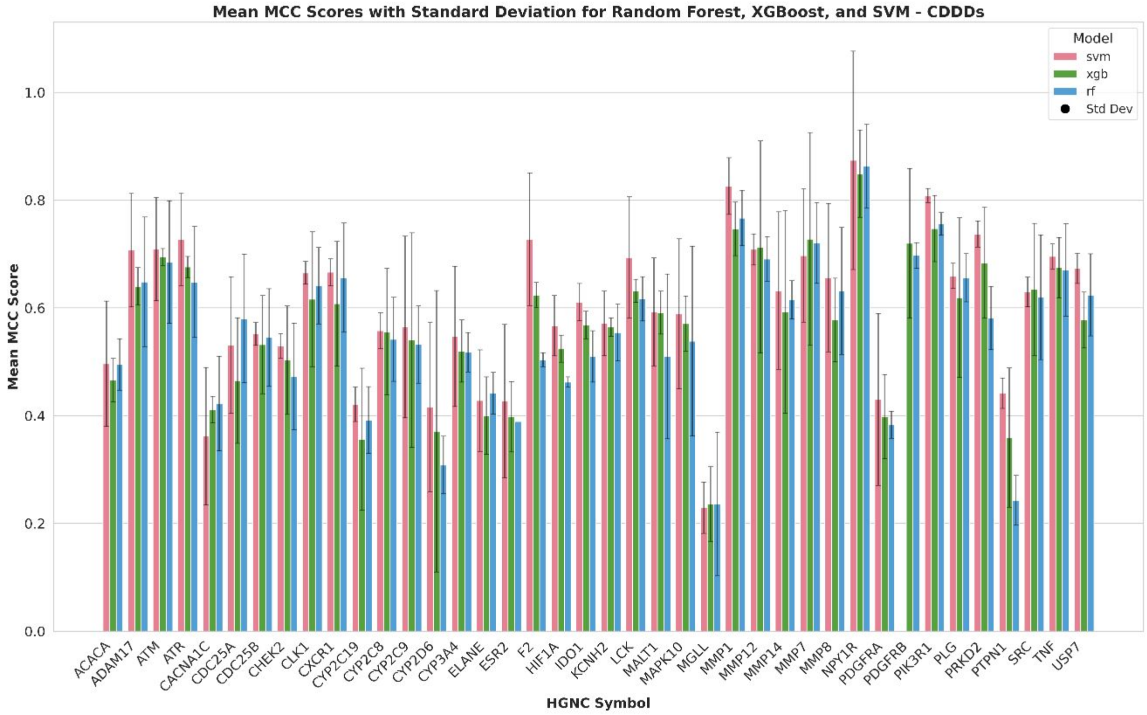

Figure S4: A bar chart illustrating the results of nested CV using Bayer AG data sets with CDDDs in combination with 3 different ML algorithms. The bar chart displays the mean MCC scores achieved by different ML models across the 40 Targets. The x-axis represents 40 targets, while the y-axis shows the mean MCC score. Each bar is color coded to represent a different ML algorithm: red for SVM, green for XGB, and blue for RF. Error bars, representing standard deviations, are displayed on each bar, indicating the variability of the MCC scores.

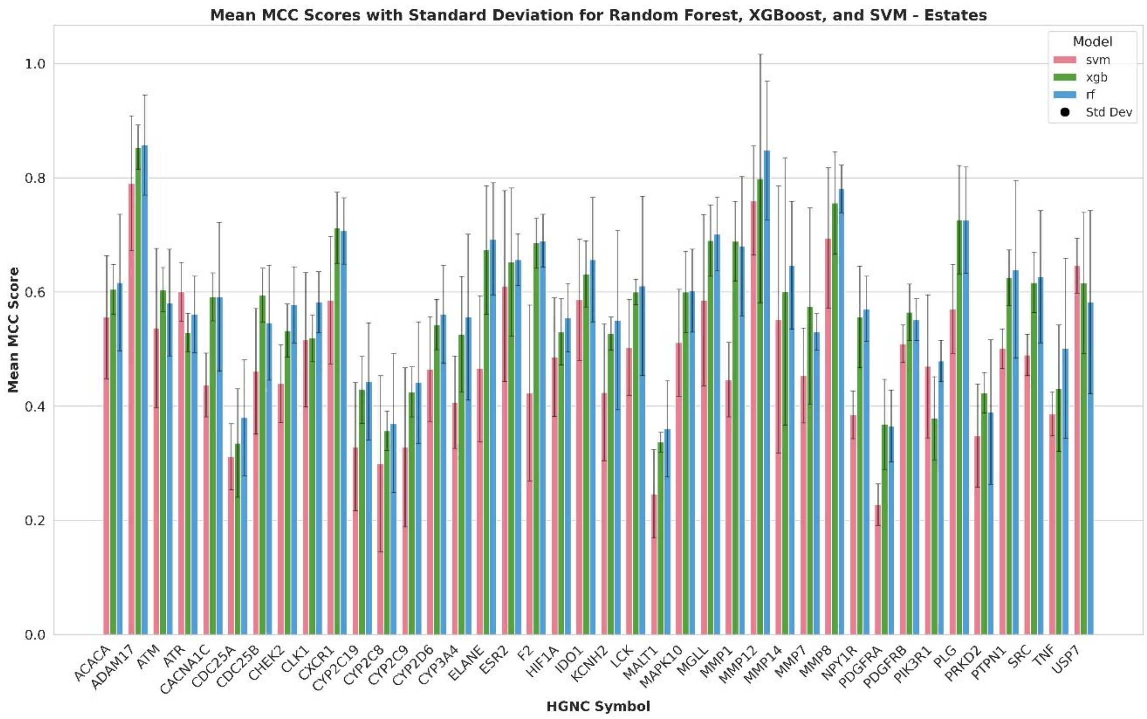

Figure: S5: A bar chart illustrating the results of nested CV using ChEMBL data sets with Estate descriptors in combination with 3 different ML algorithms. The bar chart displays the mean MCC scores achieved by different ML models across the 40 Targets. The x-axis represents 40 targets, while the y-axis shows the mean MCC score. Each bar is color coded to represent a different ML algorithm: red for SVM, green for XGB, and blue for RF. Error bars, representing standard deviations, are displayed on each bar, indicating the variability of the MCC scores.

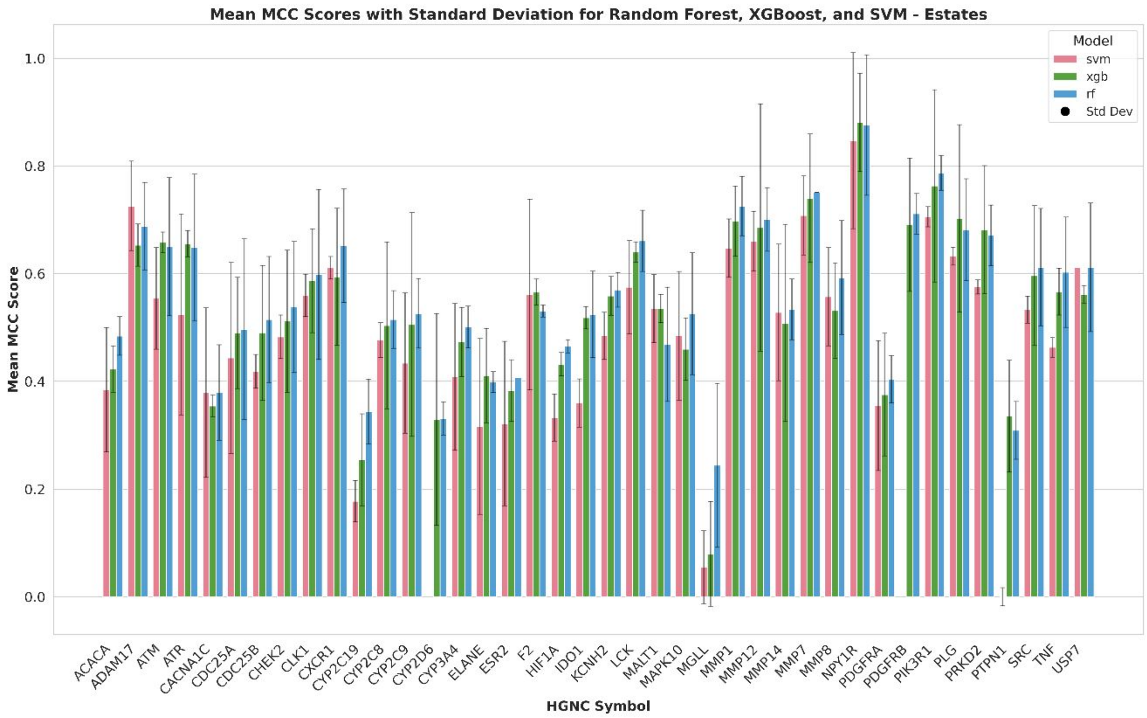

Figure S6: A bar chart illustrating the results of nested CV using Bayer AG data sets with Estate descriptors in combination with 3 different ML algorithms. The bar chart displays the mean MCC scores achieved by different ML models across the 40 Targets. The x-axis represents 40 targets, while the y-

axis shows the mean MCC score. Each bar is color coded to represent a different ML algorithm: red for SVM, green for XGB, and blue for RF. Error bars, representing standard deviations, are displayed on each bar, indicating the variability of the MCC scores.

Overall MCC Distribution from Random-based nested CV using CDDD and Estate Descriptors

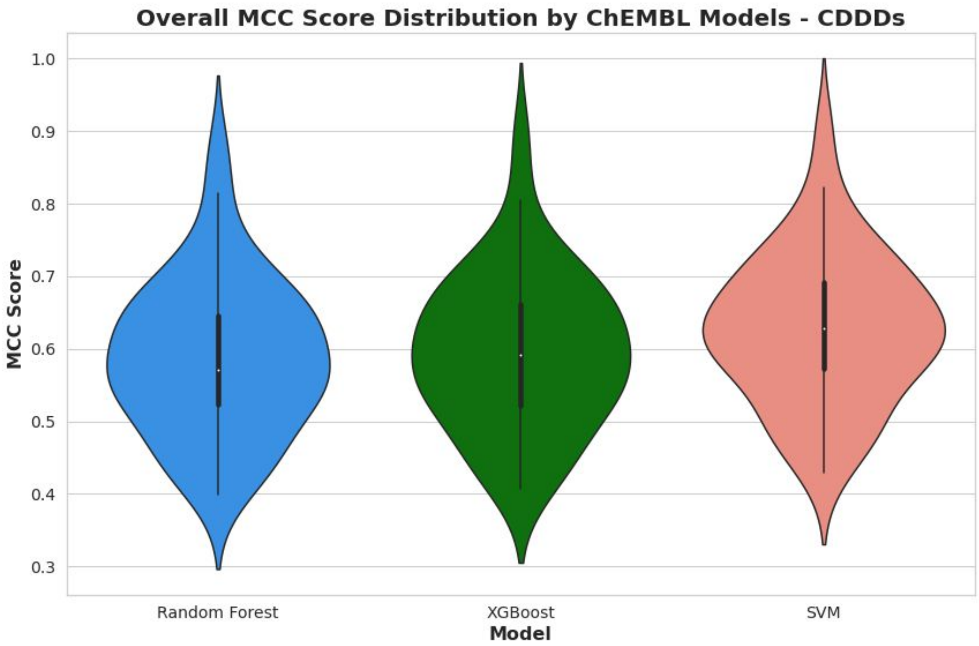

Figure S7: Illustration of the distribution of Matthews Correlation Coefficients (MCC) retrieved from the nested cross-validation for ChEMBL data sets across three machine learning techniques: Random Forest (blue), Xgboost (green), Support Vector Machine (salmon). Each technique's performance is depicted using a violin plot, which provides insights into the spread and density of MCC values when CDDDs are applied. The x-axis denotes the different ML techniques, while the y-axis represents the MCC values.

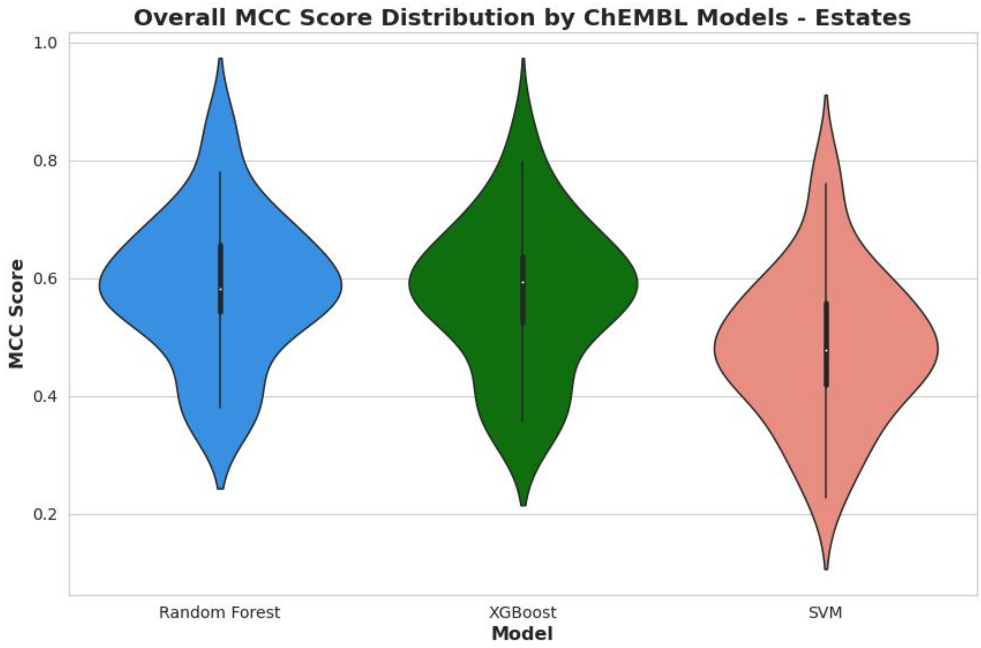

Figure S8: Illustration of the distribution of Matthews Correlation Coefficients (MCC) retrieved from the nested cross-validation for ChEMBL data sets across three machine learning techniques: Random Forest (blue), Xgboost (green), Support Vector Machine (salmon). Each technique's performance is depicted using a violin plot, which provides insights into the spread and density of MCC values when Estates are applied. The x-axis denotes the different ML techniques, while the y-axis represents the MCC values.

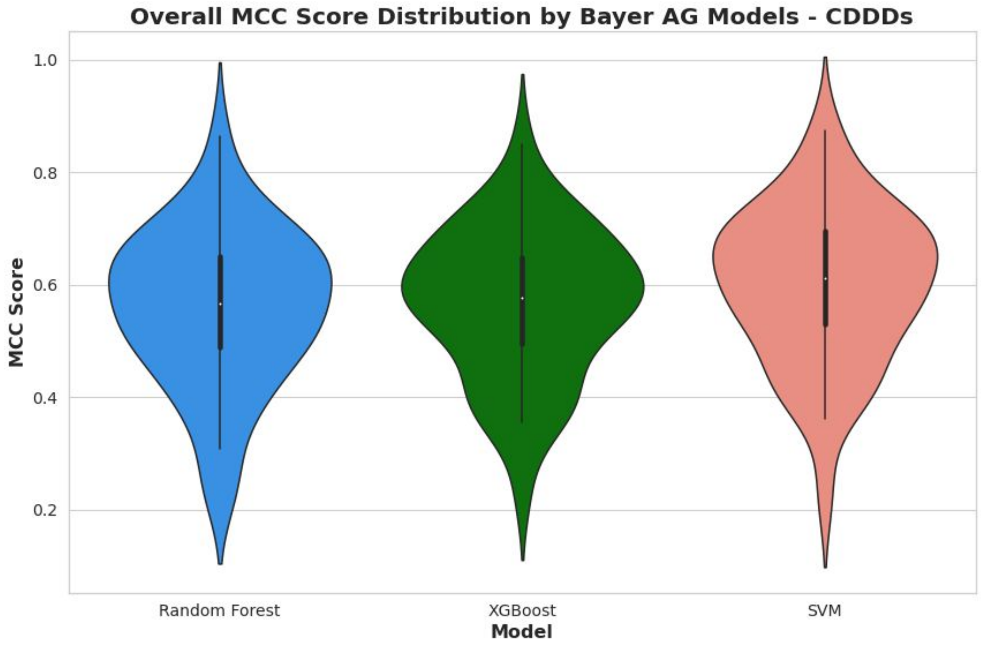

Figure S9: Illustration of the distribution of Matthews Correlation Coefficients (MCC) retrieved from the nested cross-validation for Bayer AG data sets across three machine learning techniques: Random Forest (blue), Xgboost (green), Support Vector Machine (salmon). Each technique's performance is depicted using a violin plot, which provides insights into the spread and density of

MCC values when CDDDs are applied. The x-axis denotes the different ML techniques, while the y-axis represents the MCC values.

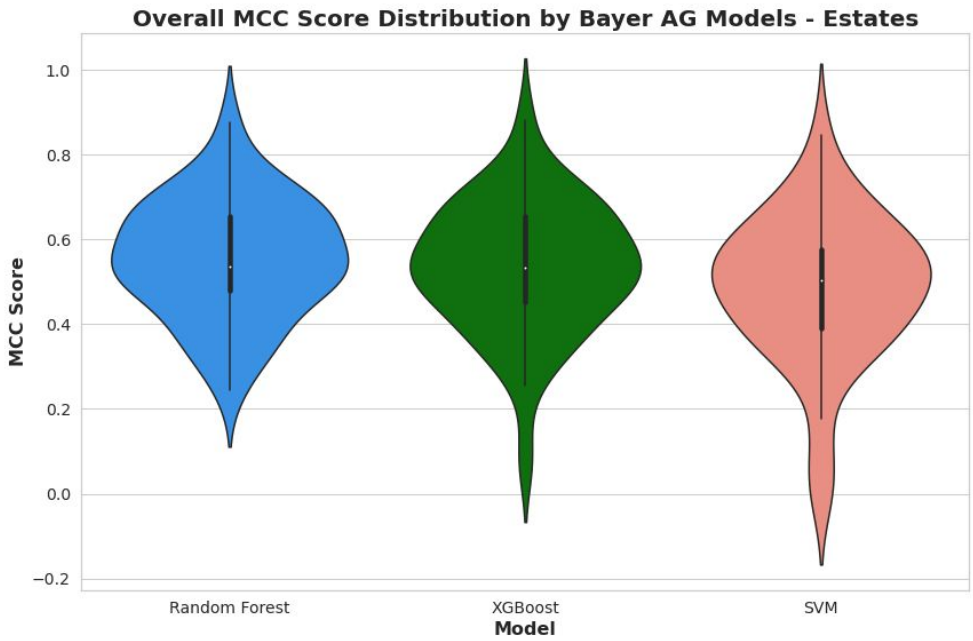

Figure S10: Illustration of the distribution of Matthews Correlation Coefficients (MCC) retrieved from the nested cross-validation for Bayer AG data sets across three machine learning techniques: Random Forest (blue), Xgboost (green), Support Vector Machine (salmon). Each technique's performance is depicted using a violin plot, which provides insights into the spread and density of MCC values when Estates are applied. The x-axis denotes the different ML techniques, while the y-axis represents the MCC values.

Bar chart illustrating the Performance of Models trained on Bayer AG data predicting ChEMBL test sets and vice versa via Estate Descriptors

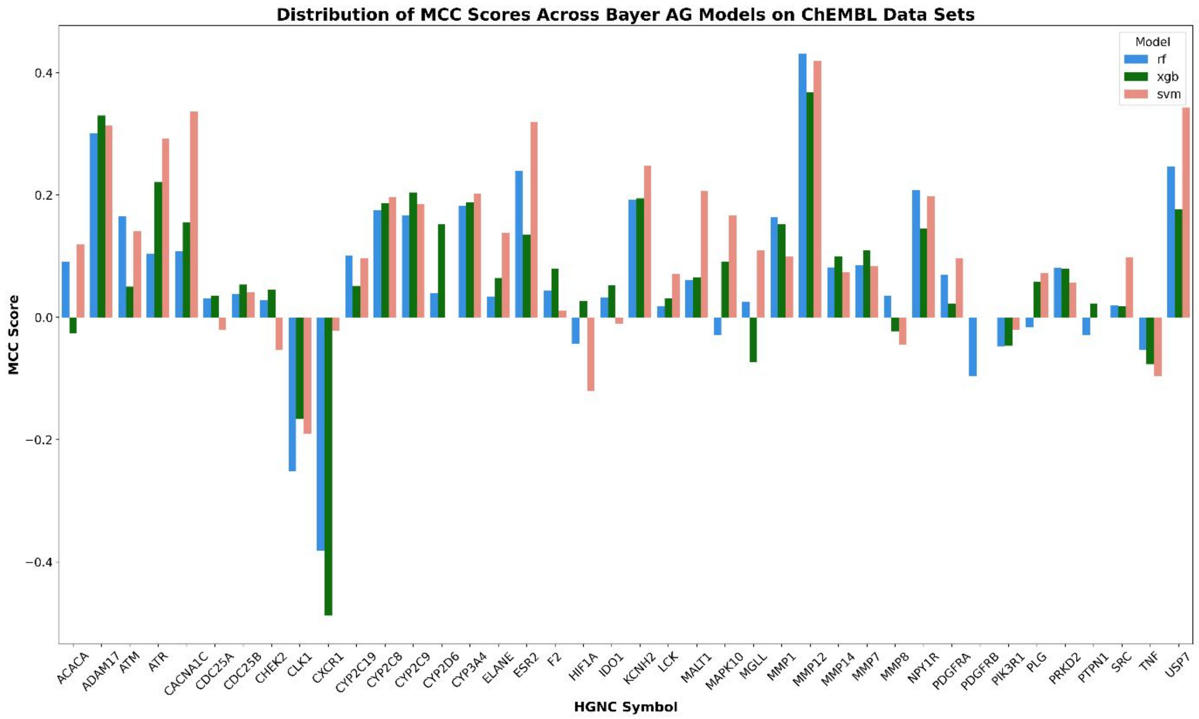

Figure S11: A bar chart illustrating the performance of models trained on Bayer AG data predicting ChEMBL test sets via Estate descriptors. The bar chart displays the MCC scores achieved by different ML algorithms across the 40 Targets. The x-axis represents 40 targets, while the y-axis shows the MCC score. Each bar is color coded to represent a different ML algorithm: red for SVM, green for XGB, and blue for RF.

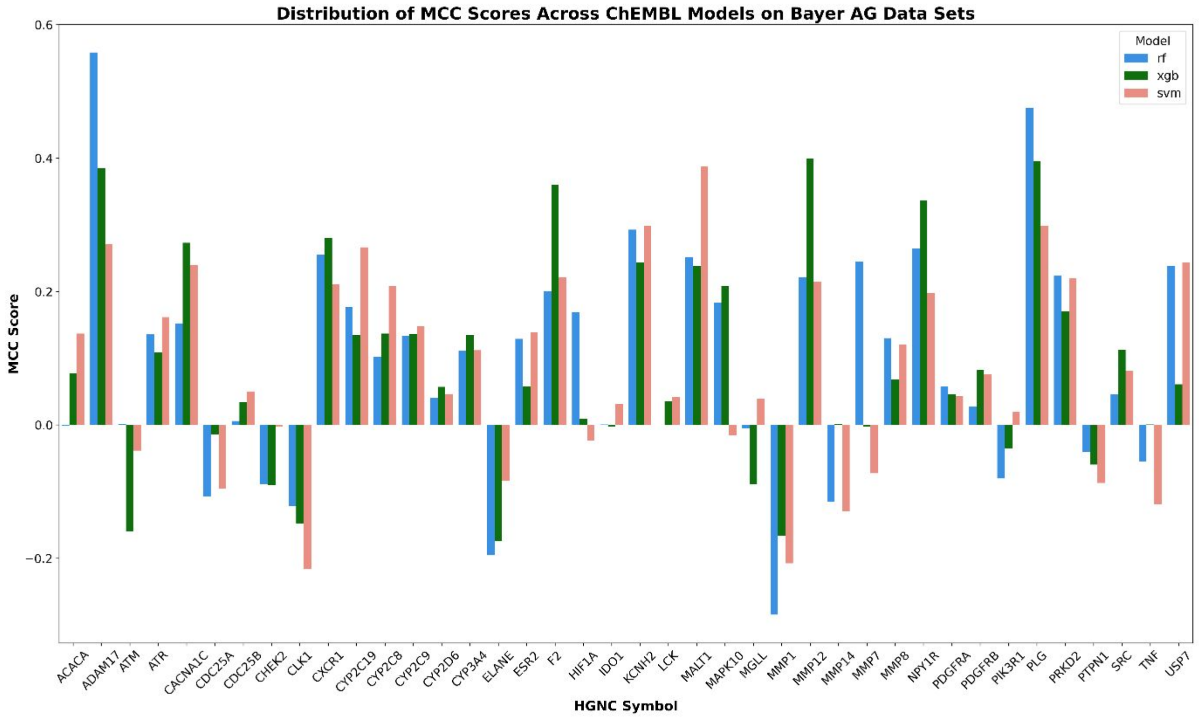

Figure S12: A bar chart illustrating the performance of models trained on ChEMBL data predicting Bayer AG test sets via Estate descriptors. The bar chart displays the MCC scores achieved by different ML algorithms across the 40 Targets. The x-axis represents 40 targets, while the y-axis shows the MCC

score. Each bar is color coded to represent a different ML model: red for SVM, green for XGB, and blue for RF.

Visualization of Chemical Space for 40 Targets

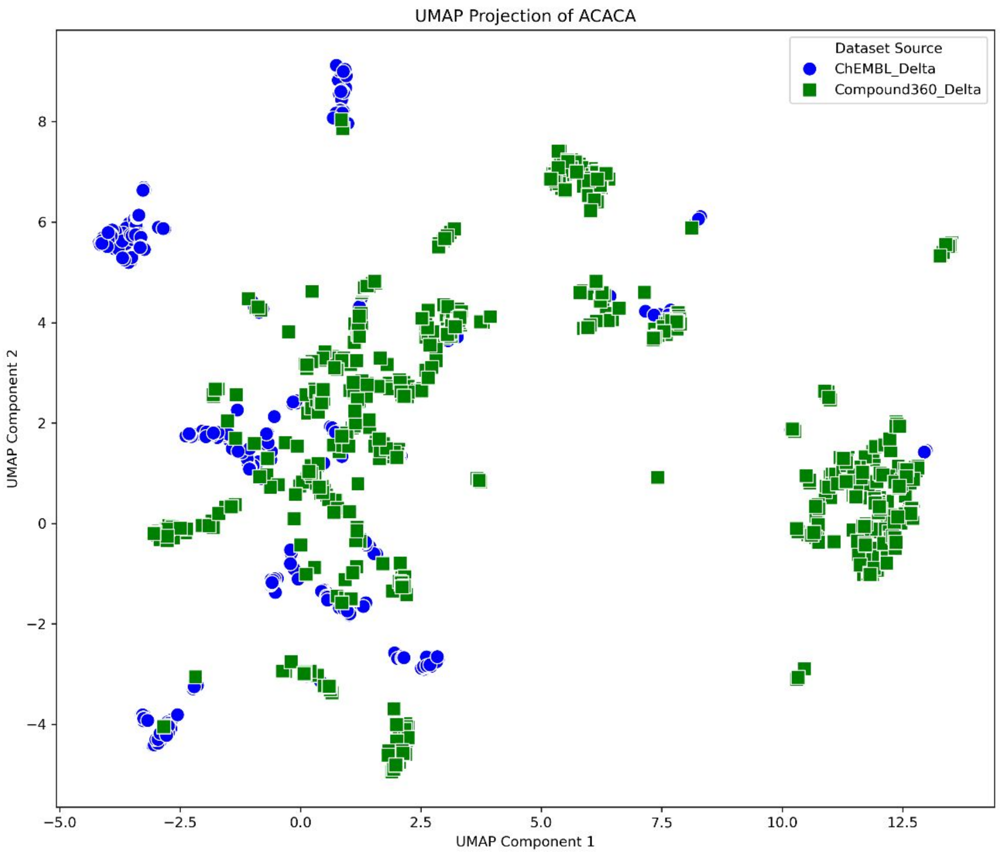

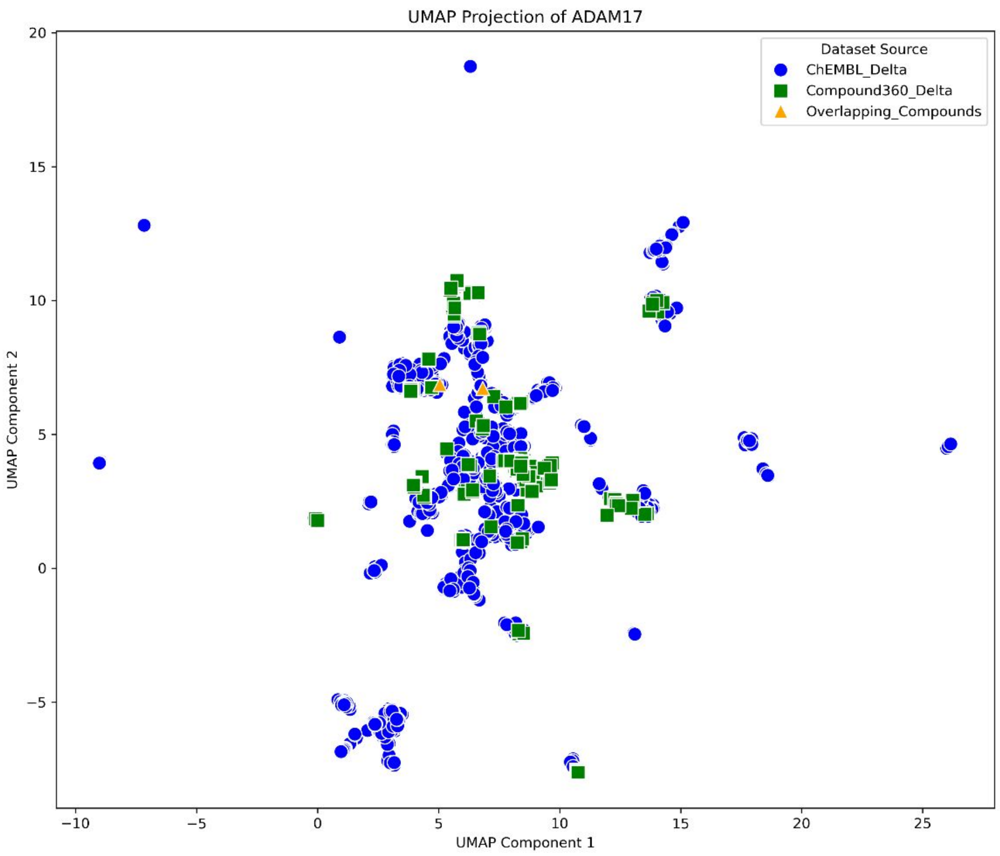

UMAP Projection of ADAM17
Dataset Source
ChEMBL_Delta
Compound360_Delta
Overlapping_Compounds
UMAP Component 1
UMAP Component 2
20
15
10
5
0
−5
−10
−5
0
5
10
15
20
25

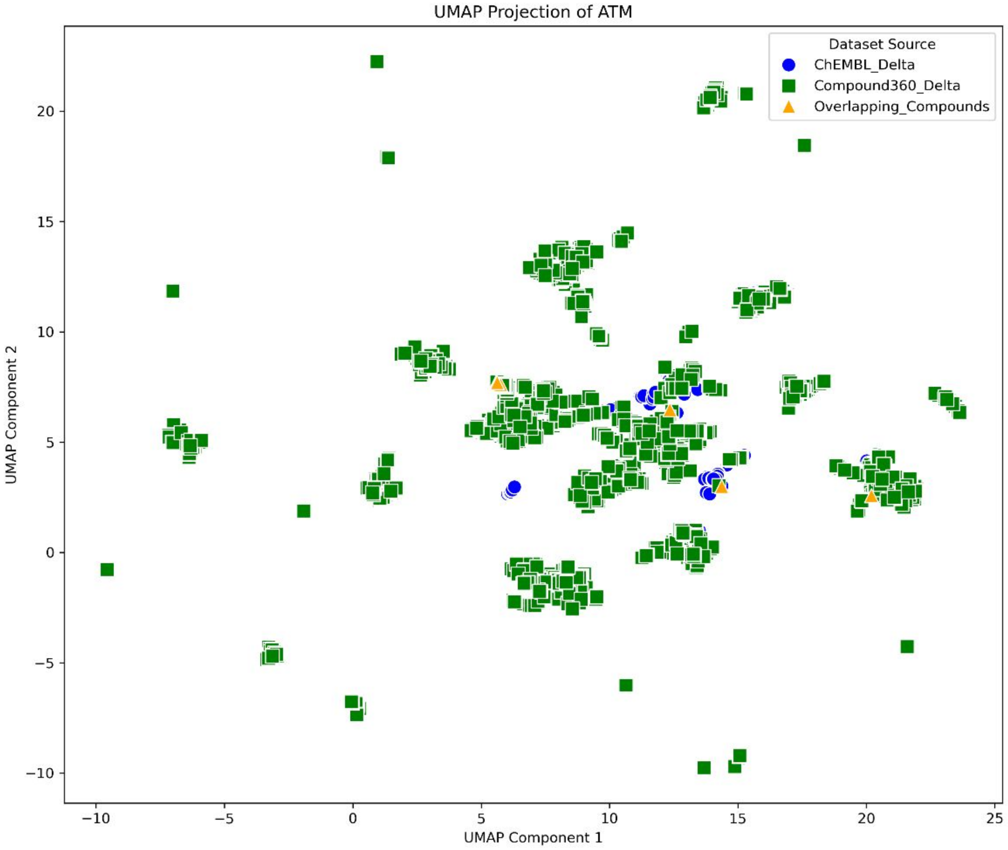
UMAP Projection of ATM
Dataset Source
ChEMBL_Delta
Compound360_Delta
Overlapping_Compounds
UMAP Component 1
UMAP Component 2

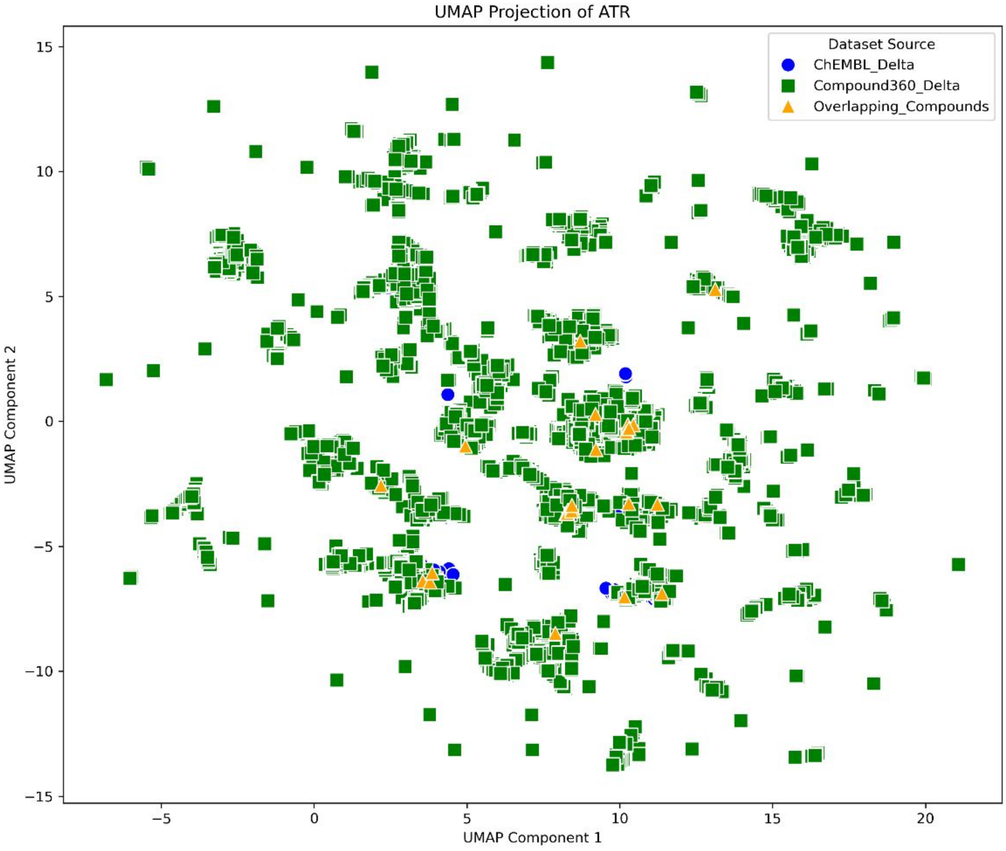

UMAP Projection of ATR
Dataset Source
ChEMBL_Delta
Compound360_Delta
Overlapping_Compounds
UMAP Component 1
UMAP Component 2
15
10
5
0
−5
−10
−15
−5
0
5
10
15
20

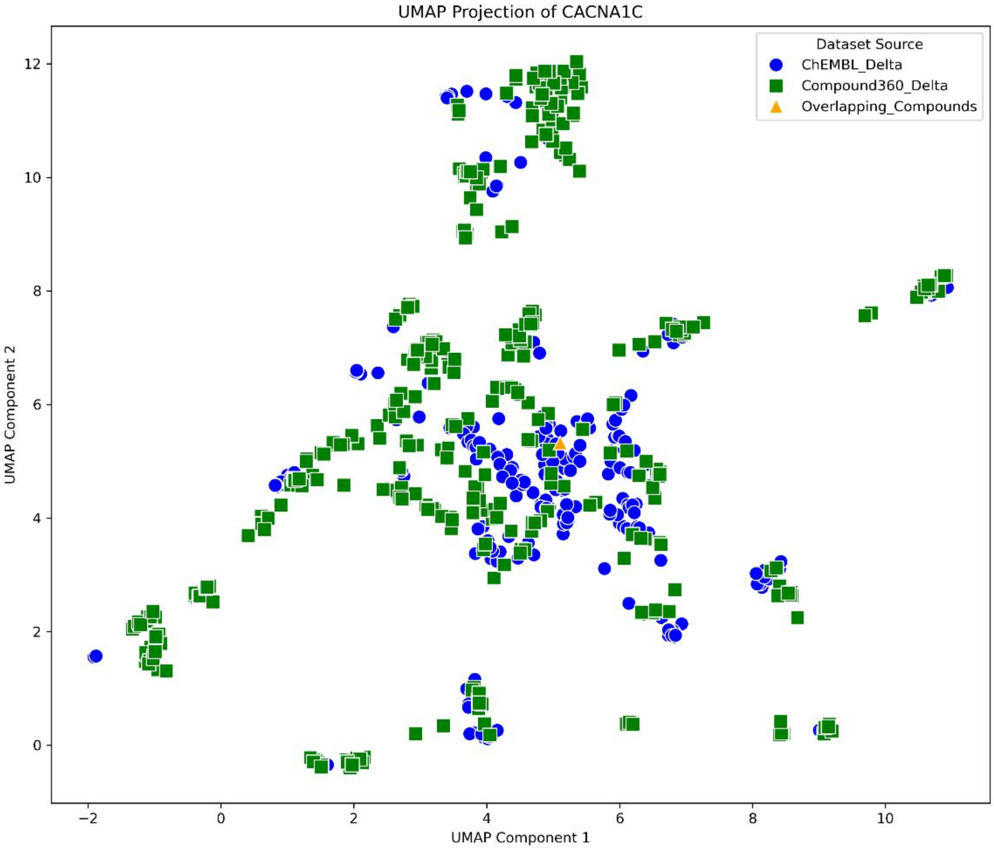
UMAP Projection of CACNA1C
Dataset Source
ChEMBL_Delta
Compound360_Delta
Overlapping_Compounds
UMAP Component 1
UMAP Component 2
−2
0
2
4
6
8
10
12

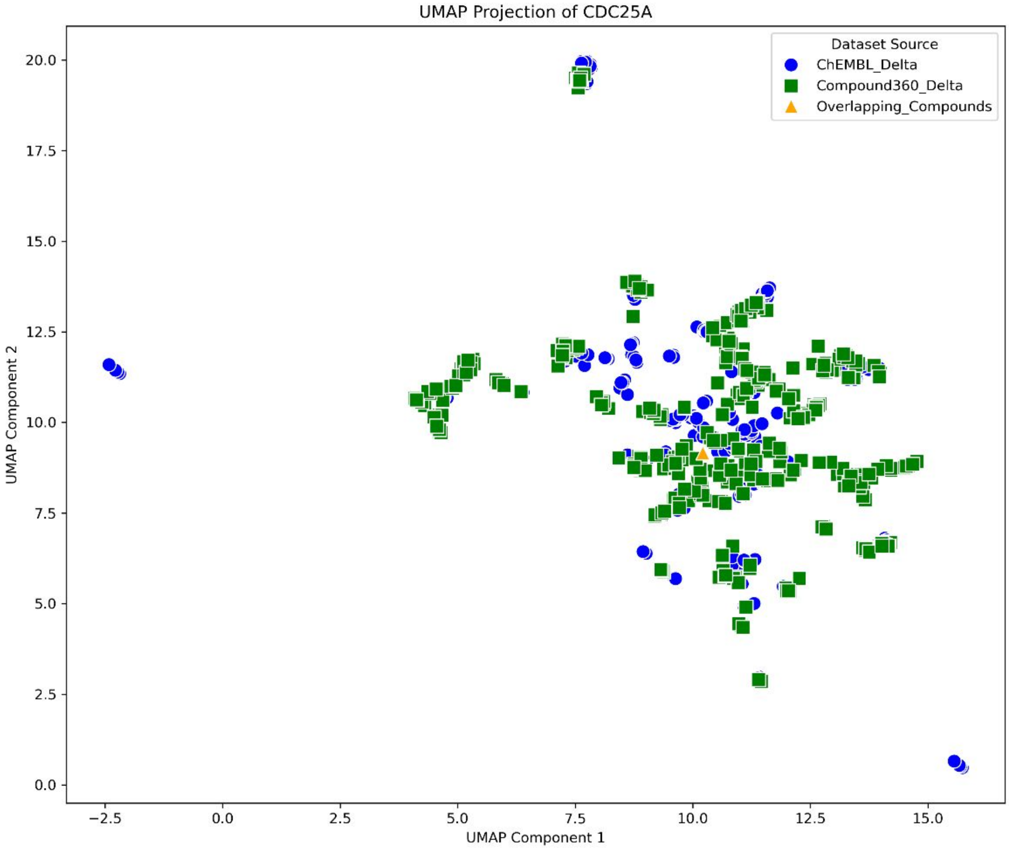

UMAP Projection of CDC25A
Dataset Source
ChEMBL_Delta
Compound360_Delta
Overlapping_Compounds
20.0
17.5
15.0
12.5
10.0
7.5
5.0
2.5
0.0
UMAP Component 2
−2.5
0.0
2.5
5.0
7.5
10.0
12.5
15.0
UMAP Component 1

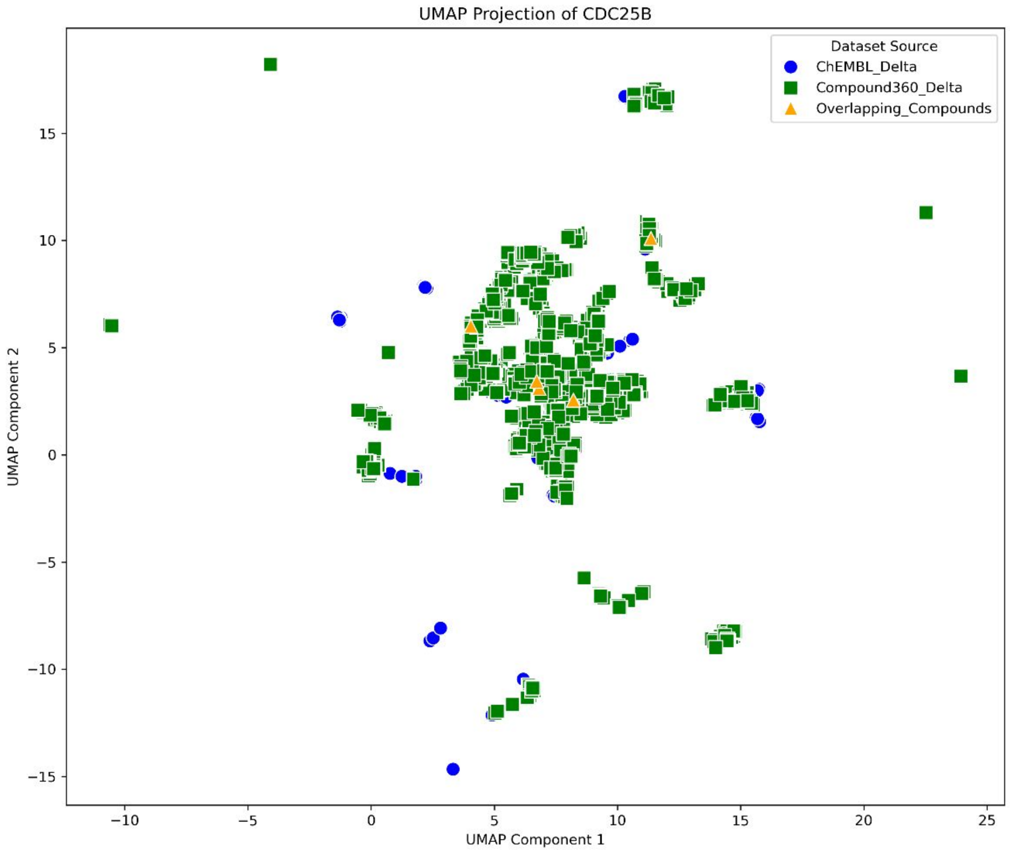

UMAP Projection of CDC25B
Dataset Source
ChEMBL_Delta
Compound360_Delta
Overlapping_Compounds
UMAP Component 1
UMAP Component 2
−10
−5
0
5
10
15
20
25
15
10
5
0
−5
−10
−15

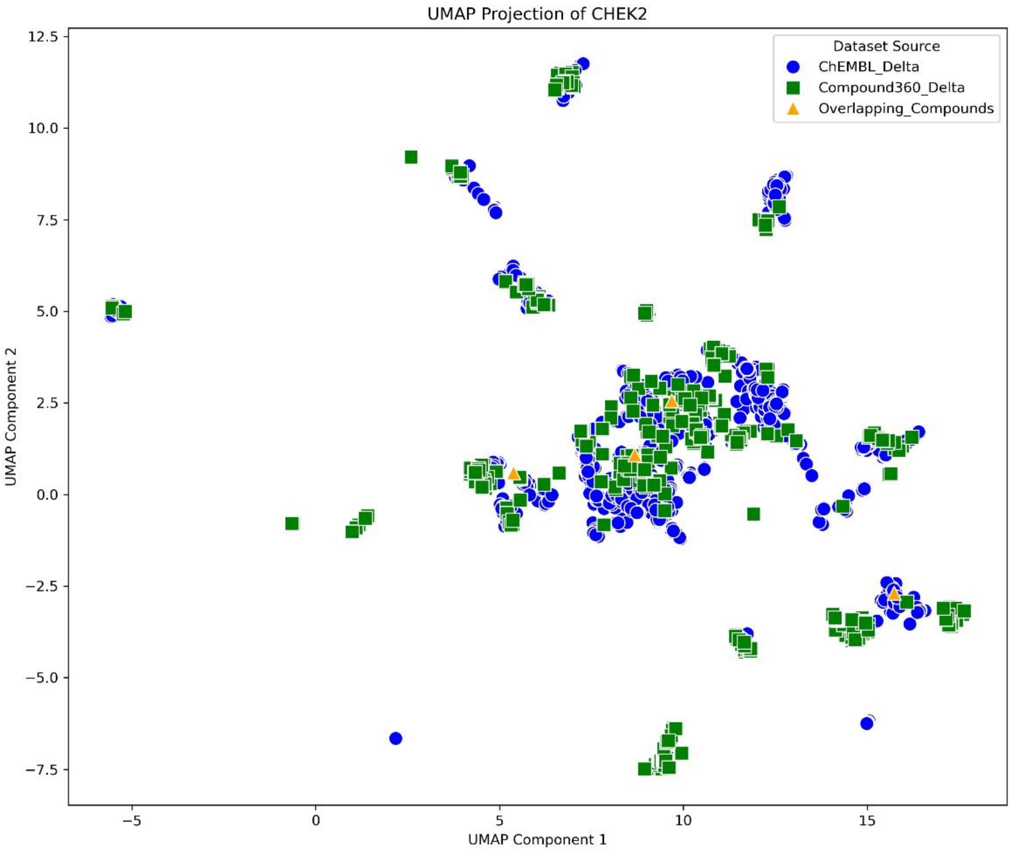

UMAP Projection of CHEK2
Dataset Source
ChEMBL_Delta
Compound360_Delta
Overlapping_Compounds
UMAP Component 2
UMAP Component 1
12.5
10.0
7.5
5.0
2.5
0.0
−2.5
−5.0
−7.5
−5
0
5
10
15

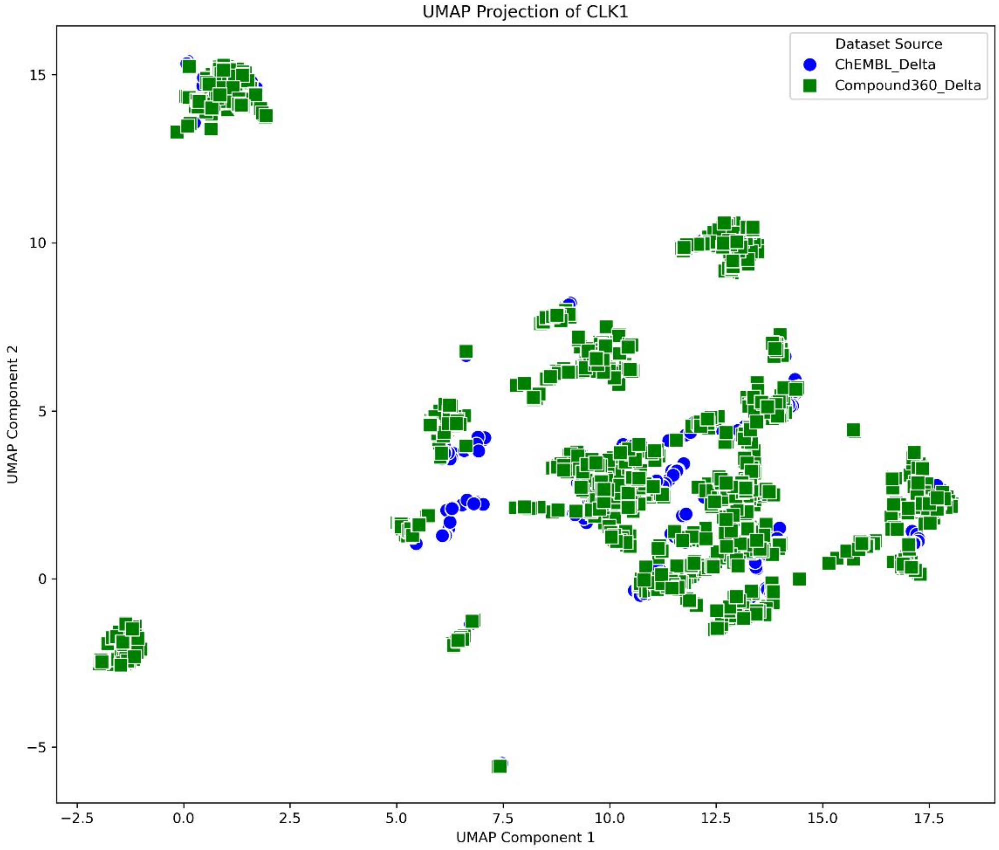
UMAP Projection of CLK1
Dataset Source
ChEMBL_Delta
Compound360_Delta
UMAP Component 2
UMAP Component 1
15
10
5
0
−5
−2.5
0.0
2.5
5.0
7.5
10.0
12.5
15.0
17.5

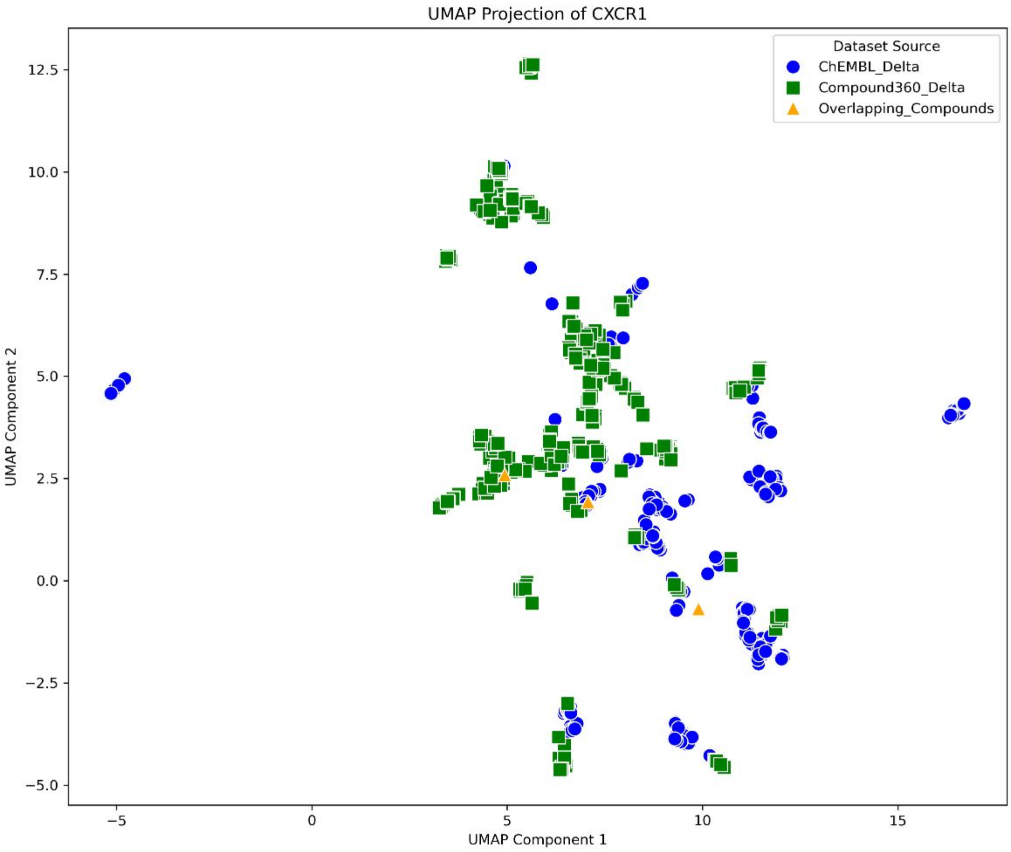

UMAP Projection of CXCR1
Dataset Source
ChEMBL_Delta
Compound360_Delta
Overlapping_Compounds
UMAP Component 2
UMAP Component 1
12.5
10.0
7.5
5.0
2.5
0.0
−2.5
−5.0
−5
0
5
10
15

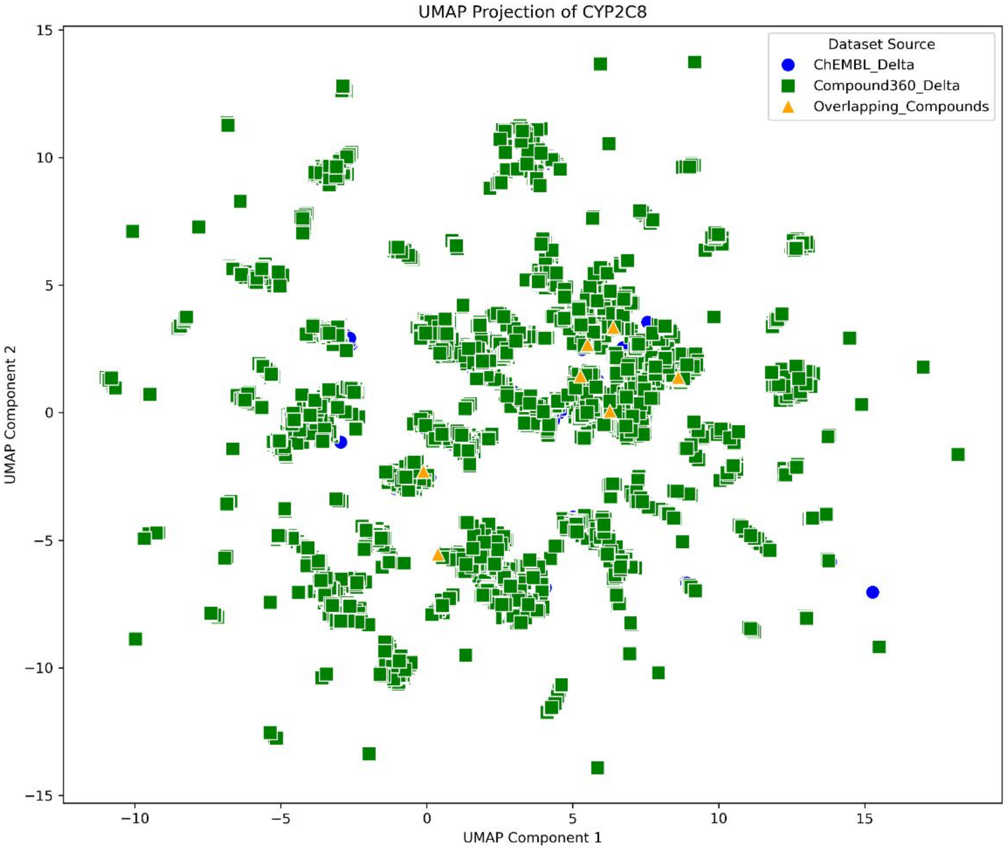
UMAP Projection of CYP2C8
Dataset Source
ChEMBL_Delta
Compound360_Delta
Overlapping_Compounds
UMAP Component 2
UMAP Component 1
15
10
5
0
−5
−10
−15
−10
−5
0
5
10
15

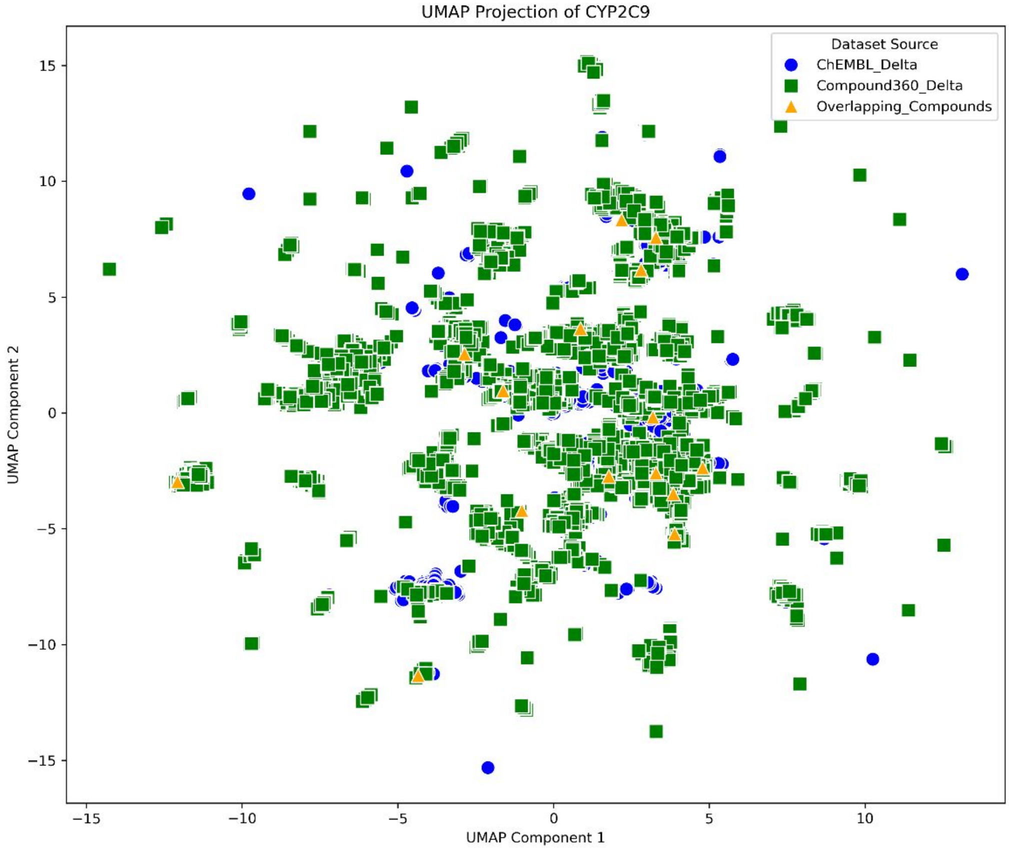
UMAP Projection of CYP2C9
Dataset Source
ChEMBL_Delta
Compound360_Delta
Overlapping_Compounds
UMAP Component 1
UMAP Component 2
−15
−10
−5
0
5
10
15

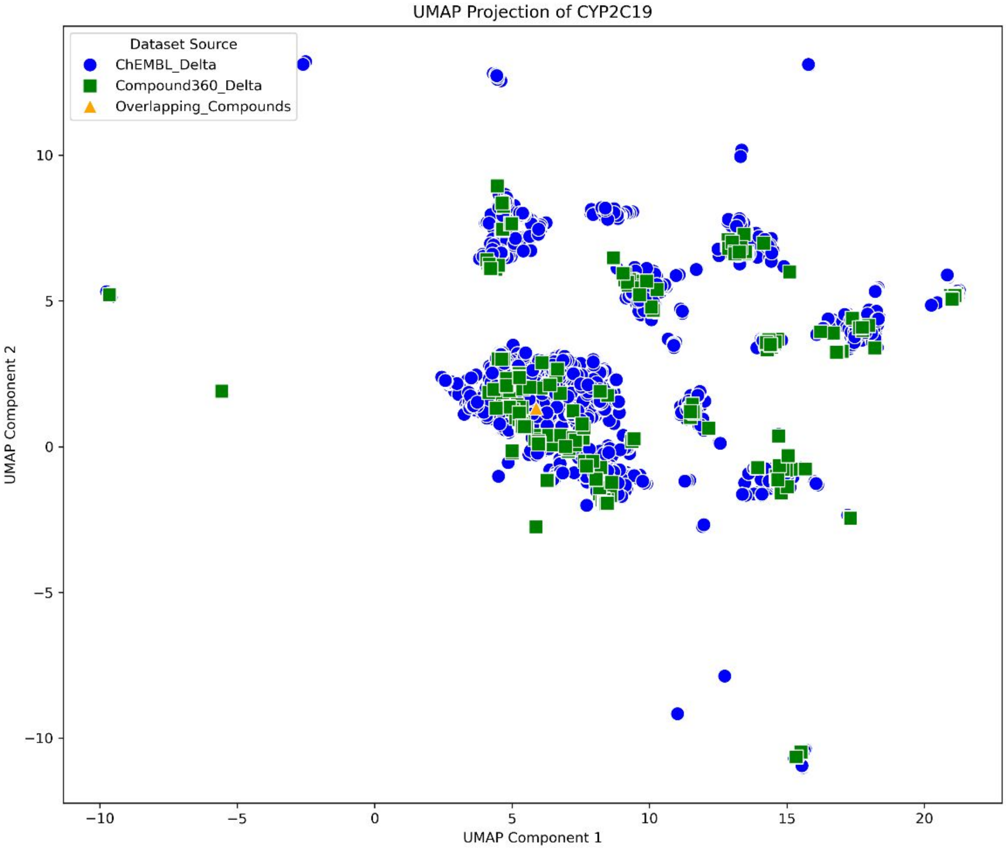

UMAP Projection of CYP2C19
Dataset Source
ChEMBL_Delta
Compound360_Delta
Overlapping_Compounds
10
5
0
−5
−10
UMAP Component 2
−10
−5
0
5
10
15
20
UMAP Component 1

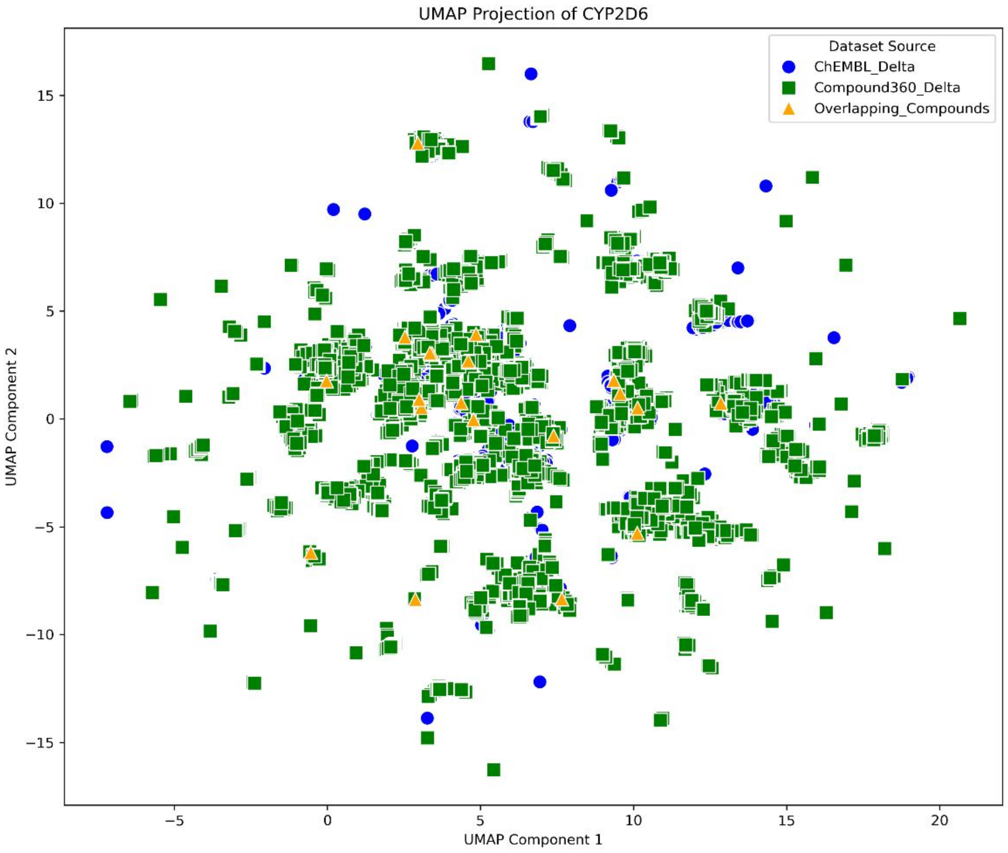
UMAP Projection of CYP2D6
Dataset Source
ChEMBL_Delta
Compound360_Delta
Overlapping_Compounds
UMAP Component 1
UMAP Component 2
−5
0
5
10
15
20
−15
−10
−5
0
5
10
15

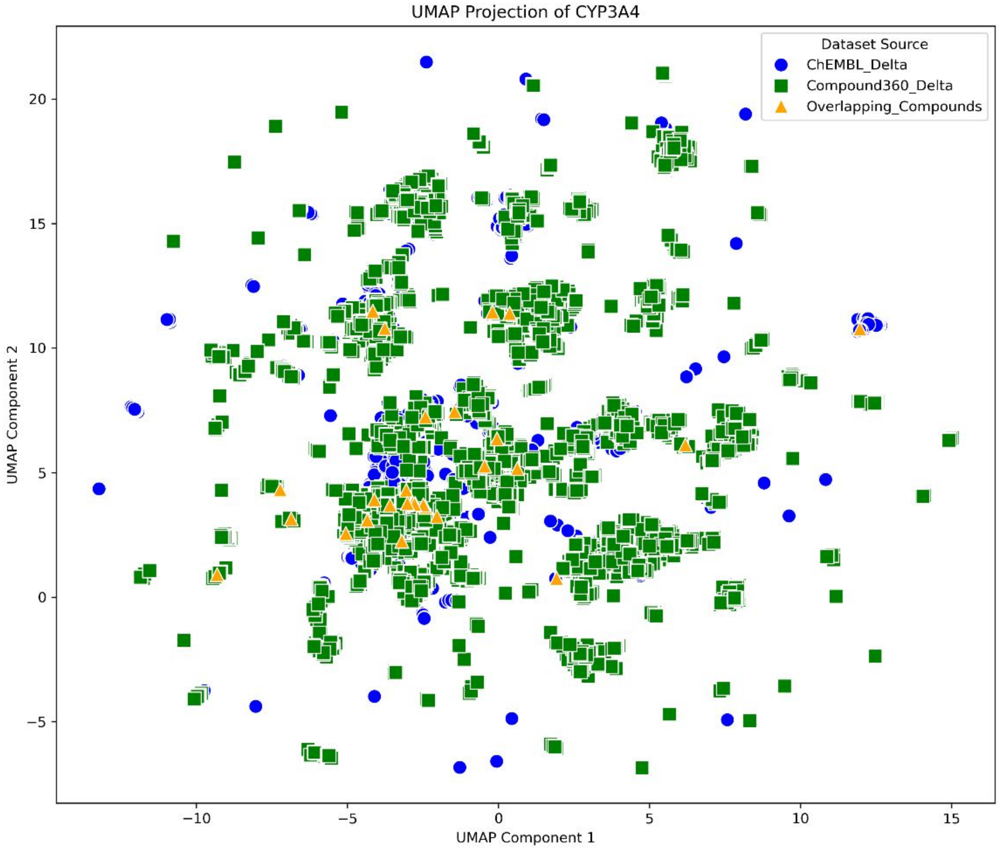
UMAP Projection of CYP3A4
Dataset Source
ChEMBL_Delta
Compound360_Delta
Overlapping_Compounds
UMAP Component 1
UMAP Component 2
−10
−5
0
5
10
15
20

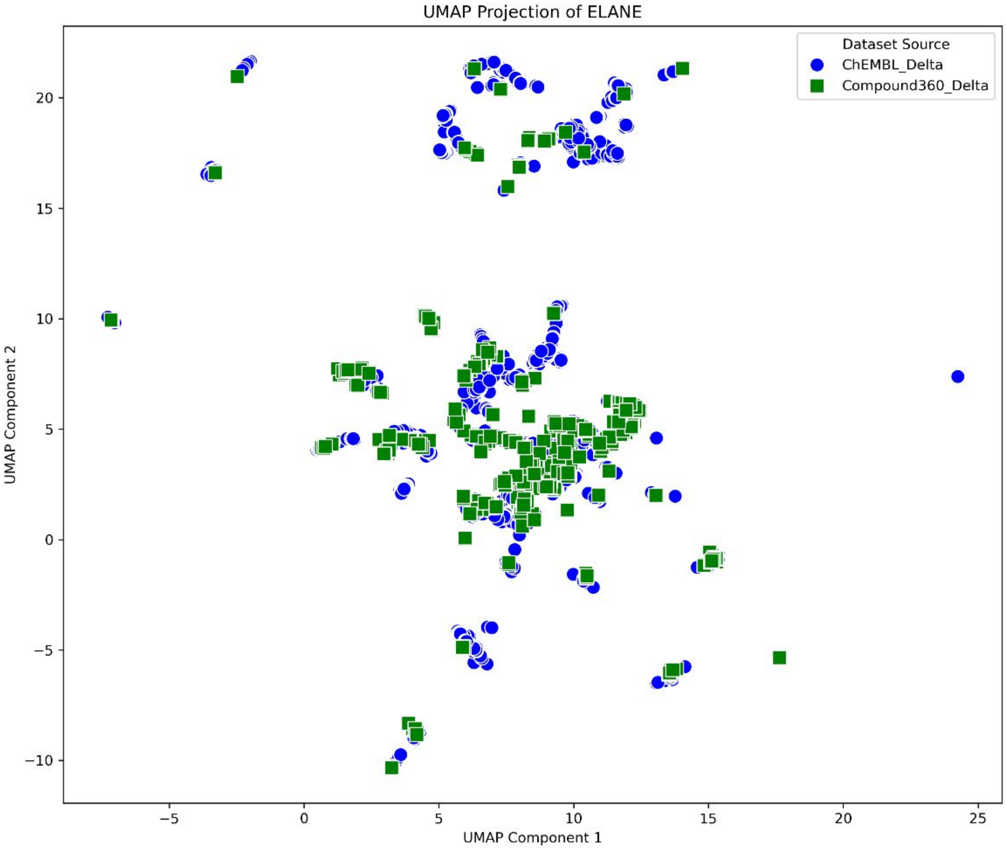
UMAP Projection of ELANE
Dataset Source
ChEMBL_Delta
Compound360_Delta
UMAP Component 1
UMAP Component 2
20
15
10
5
0
−5
−10
−5
0
5
10
15
20
25

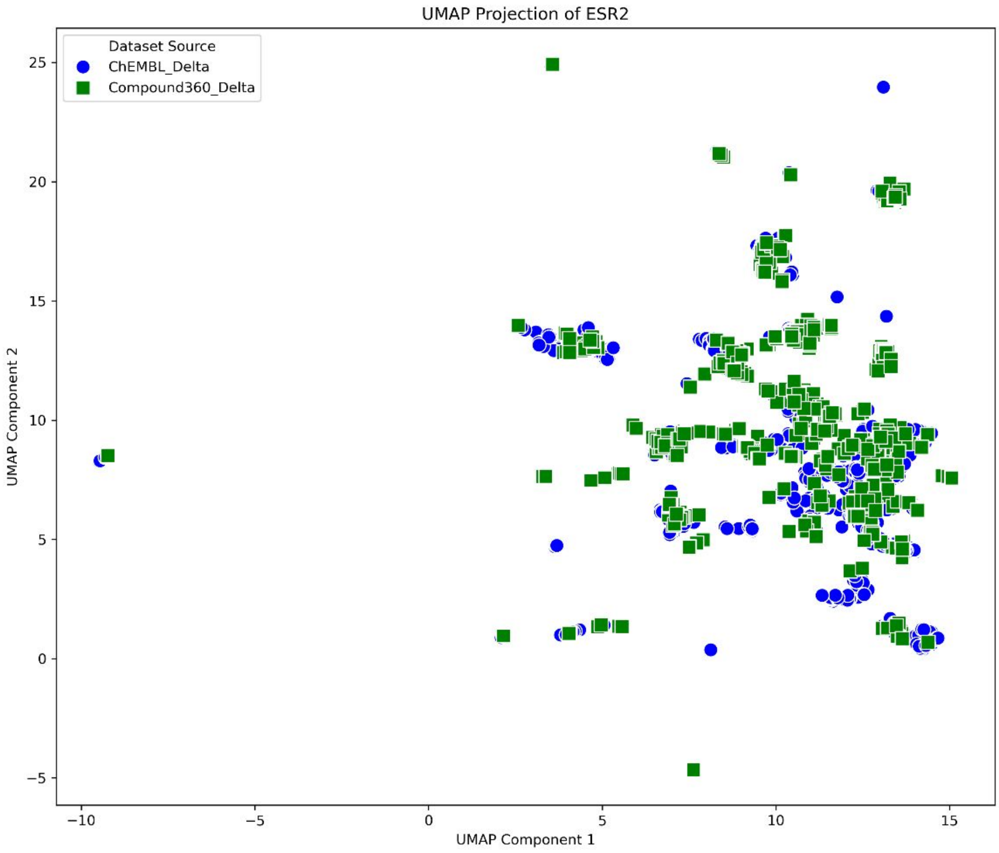
UMAP Projection of ESR2
Dataset Source
ChEMBL_Delta
Compound360_Delta
UMAP Component 1
UMAP Component 2
−10
−5
0
5
10
15
20
25

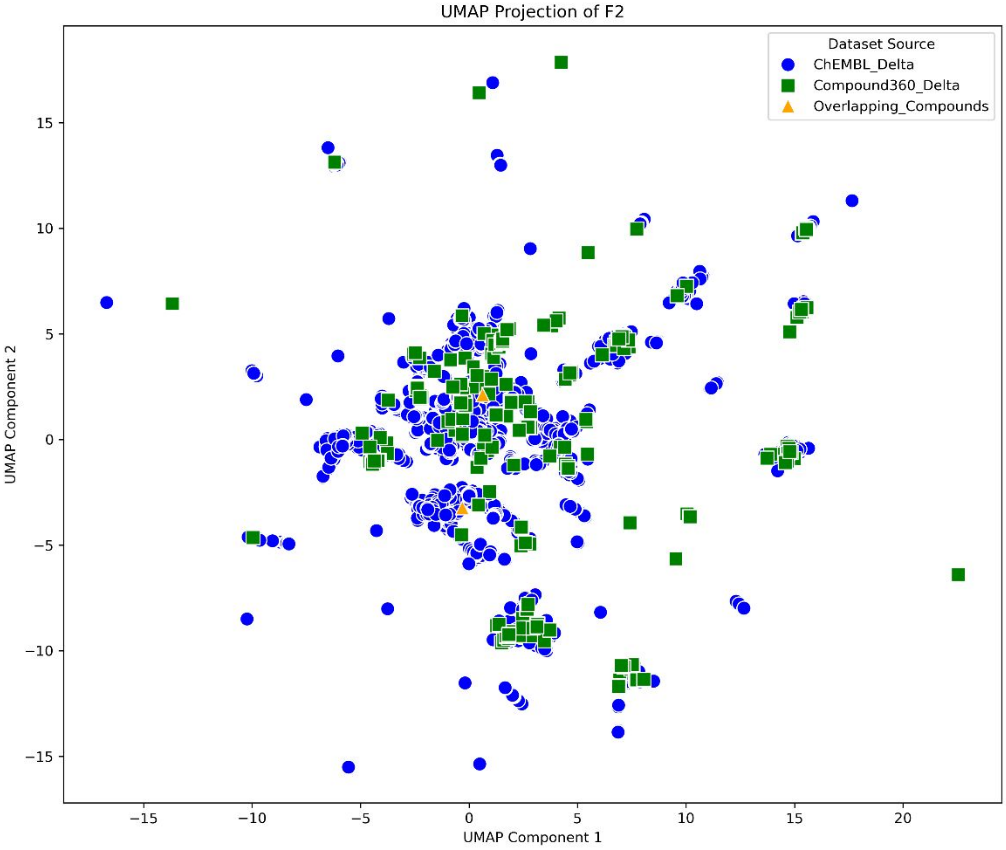

UMAP Projection of F2
Dataset Source
ChEMBL_Delta
Compound360_Delta
Overlapping_Compounds
UMAP Component 2
UMAP Component 1
15
10
5
0
−5
−10
−15
−15
−10
−5
0
5
10
15
20

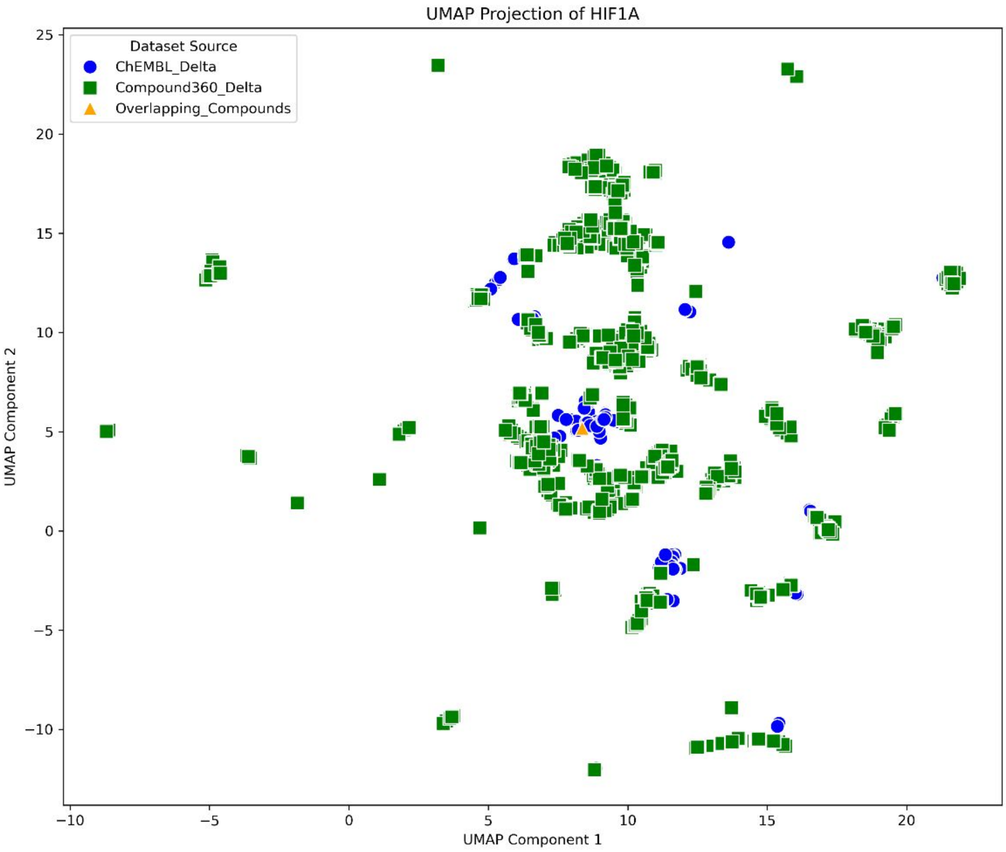
UMAP Projection of HIF1A
Dataset Source
ChEMBL_Delta
Compound360_Delta
Overlapping_Compounds
UMAP Component 2
UMAP Component 1
25
20
15
10
5
0
−5
−10
−10
−5
0
5
10
15
20

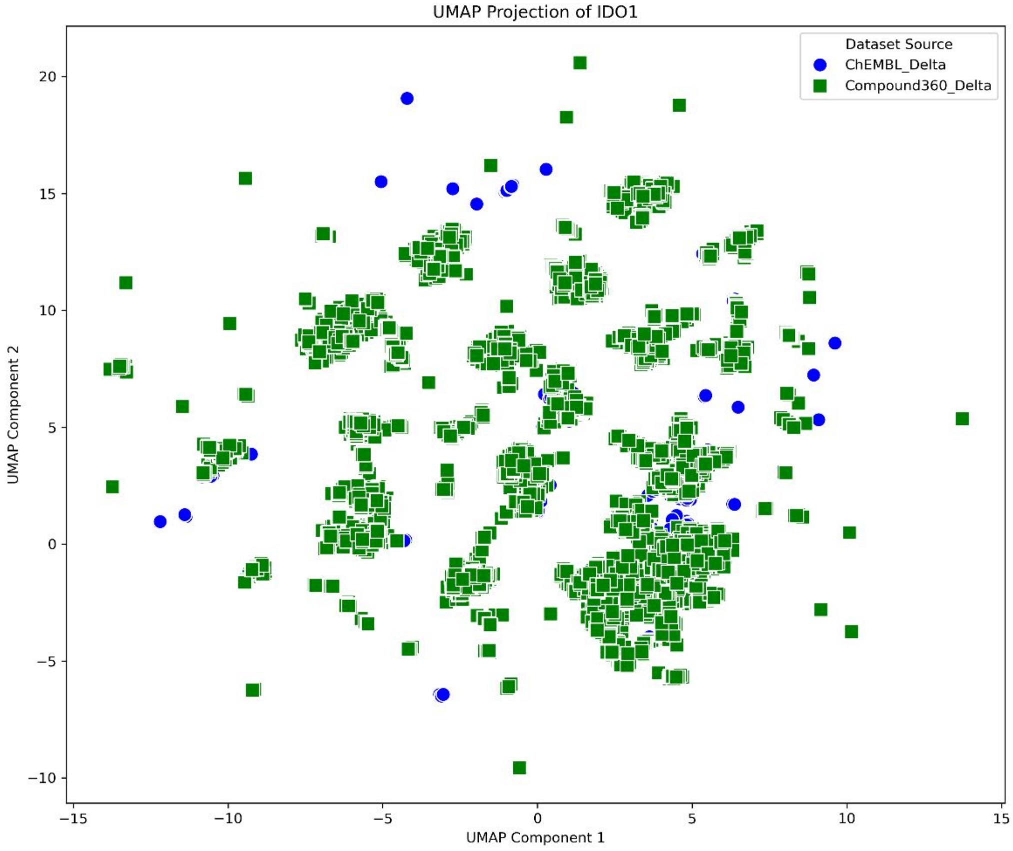

UMAP Projection of IDO1
Dataset Source
ChEMBL_Delta
Compound360_Delta
UMAP Component 2
UMAP Component 1
20
15
10
5
0
−5
−10
−15
−10
−5
0
5
10
15

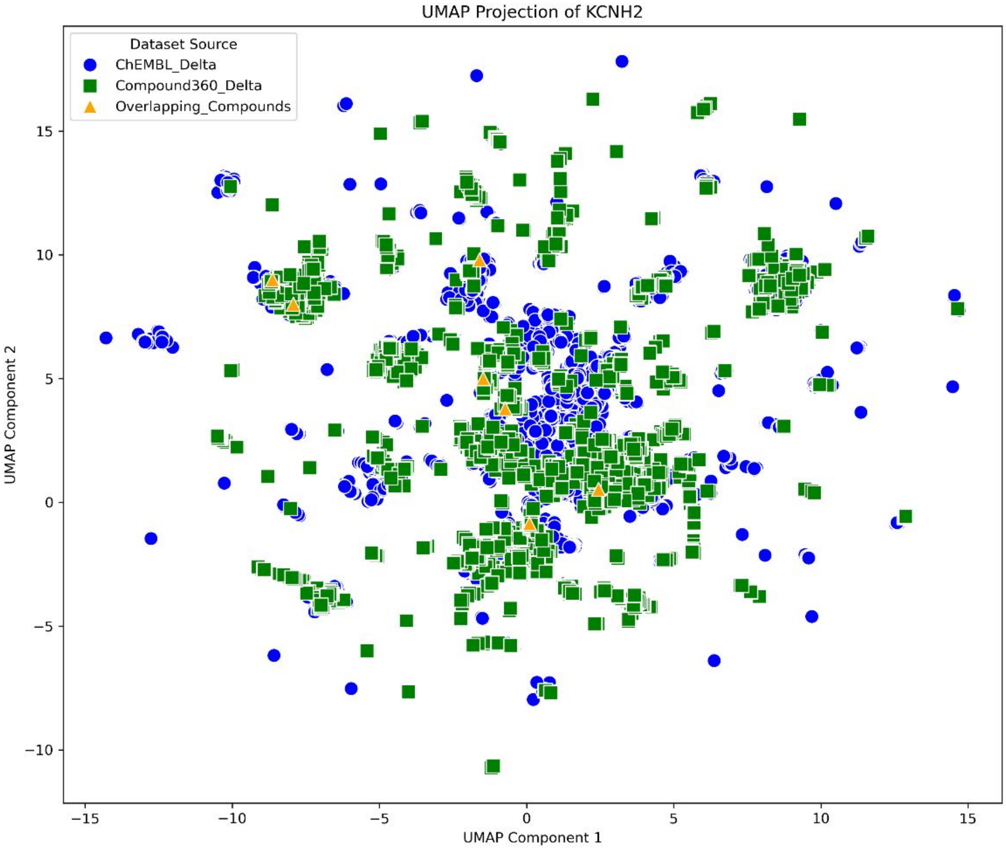
UMAP Projection of KCNH2
Dataset Source
ChEMBL_Delta
Compound360_Delta
Overlapping_Compounds
UMAP Component 1
UMAP Component 2
−15
−10
−5
0
5
10
15

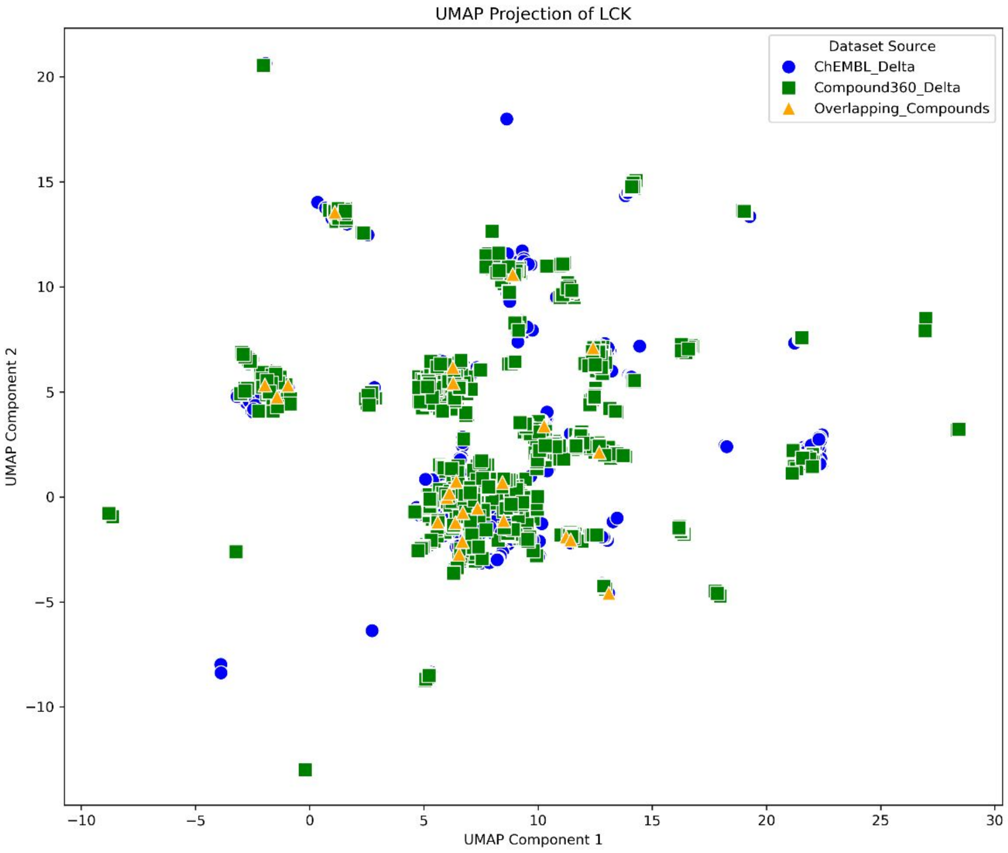

UMAP Projection of LCK
Dataset Source
ChEMBL_Delta
Compound360_Delta
Overlapping_Compounds
UMAP Component 2
UMAP Component 1
20
15
10
5
0
−5
−10
−10
−5
0
5
10
15
20
25
30

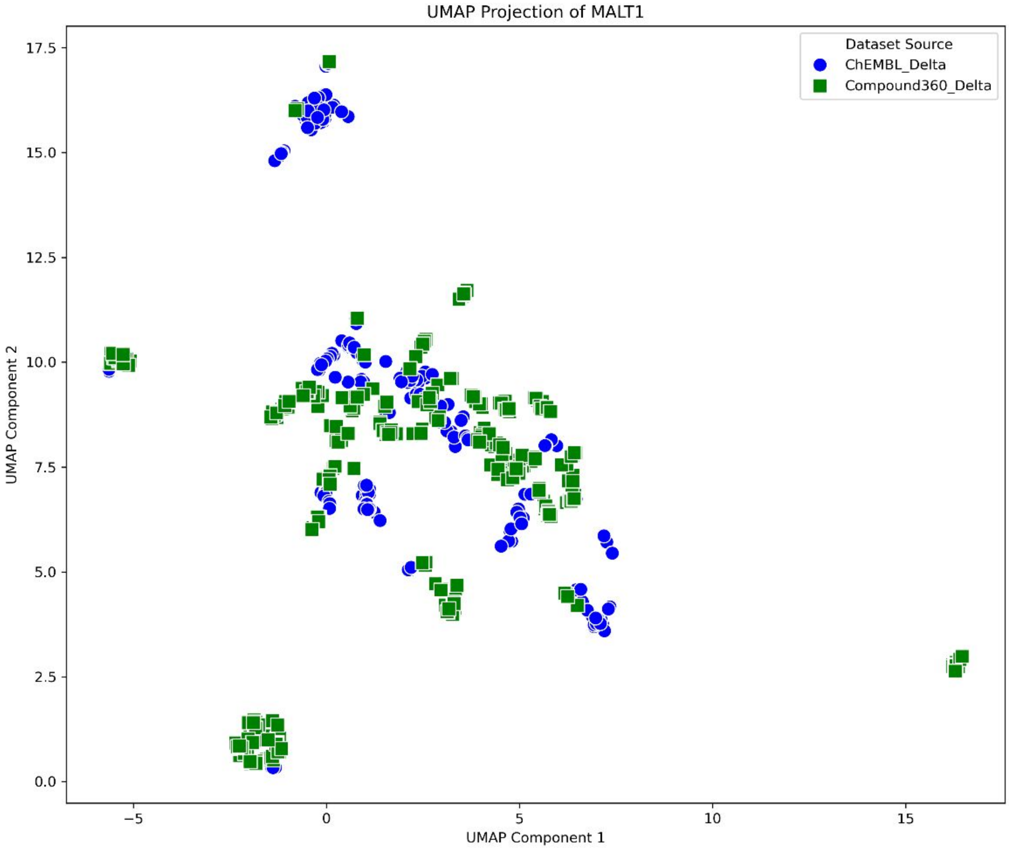
UMAP Projection of MALT1
Dataset Source
ChEMBL_Delta
Compound360_Delta
UMAP Component 2
UMAP Component 1
17.5
15.0
12.5
10.0
7.5
5.0
2.5
0.0
−5
0
5
10
15

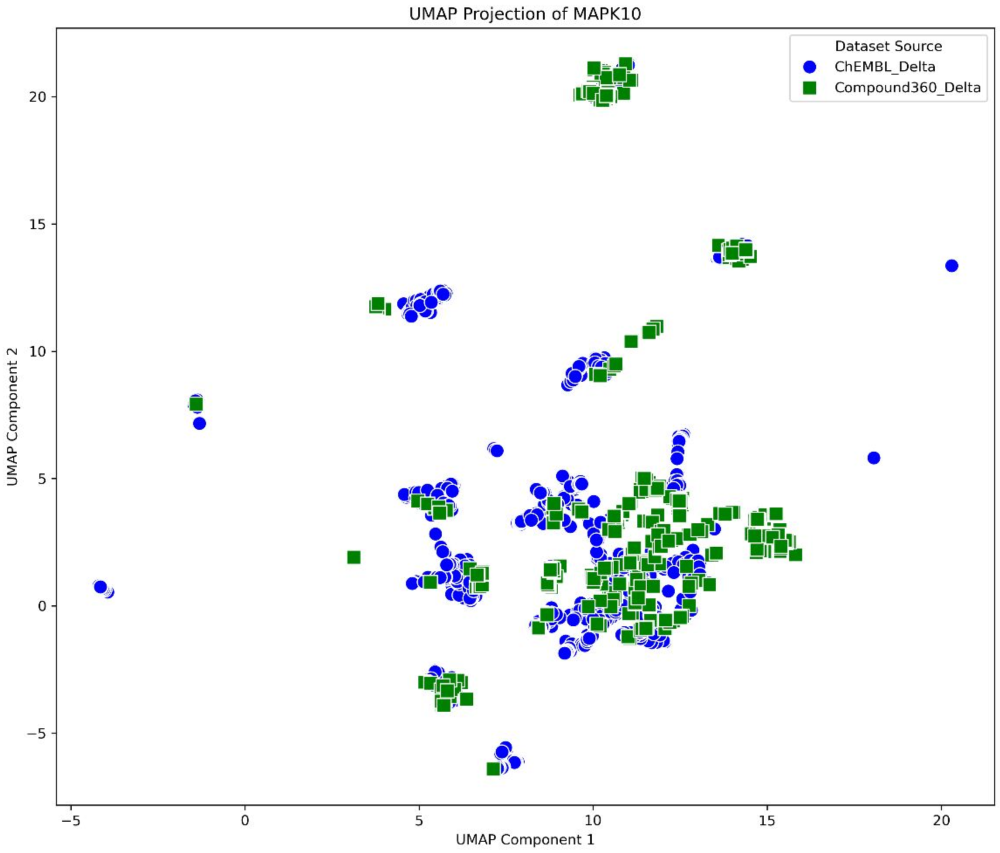
UMAP Projection of MAPK10
Dataset Source
ChEMBL_Delta
Compound360_Delta
UMAP Component 1
UMAP Component 2
−5
0
5
10
15
20
−5
0
5
10
15
20

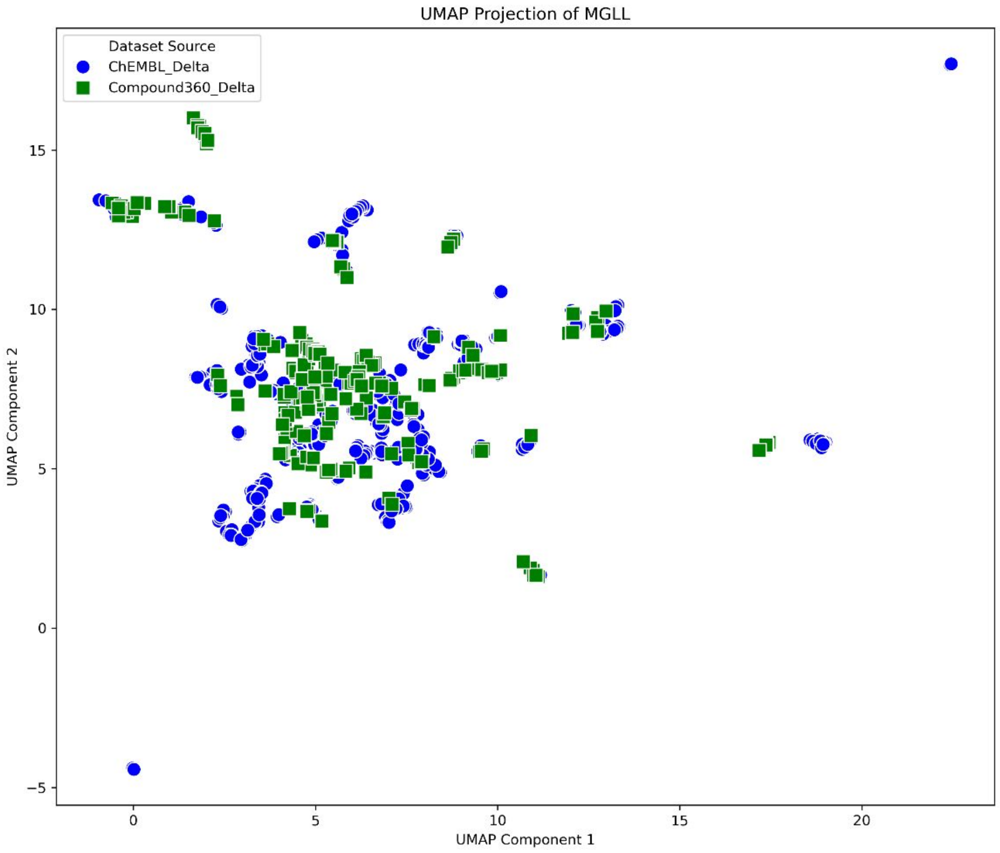
UMAP Projection of MGLL
Dataset Source
ChEMBL_Delta
Compound360_Delta
UMAP Component 1
UMAP Component 2
15
10
5
0
−5
0
5
10
15
20

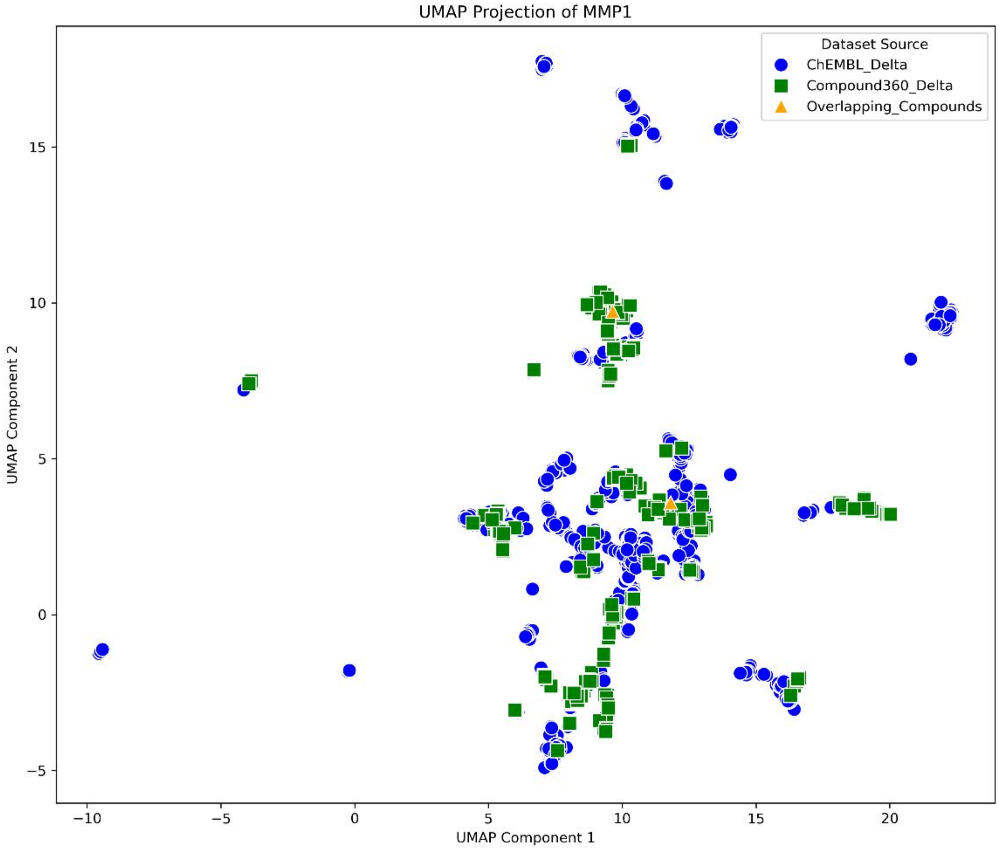
UMAP Projection of MMP1
Dataset Source
ChEMBL_Delta
Compound360_Delta
Overlapping_Compounds
UMAP Component 2
UMAP Component 1
15
10
5
0
−5
−10
−5
0
5
10
15
20

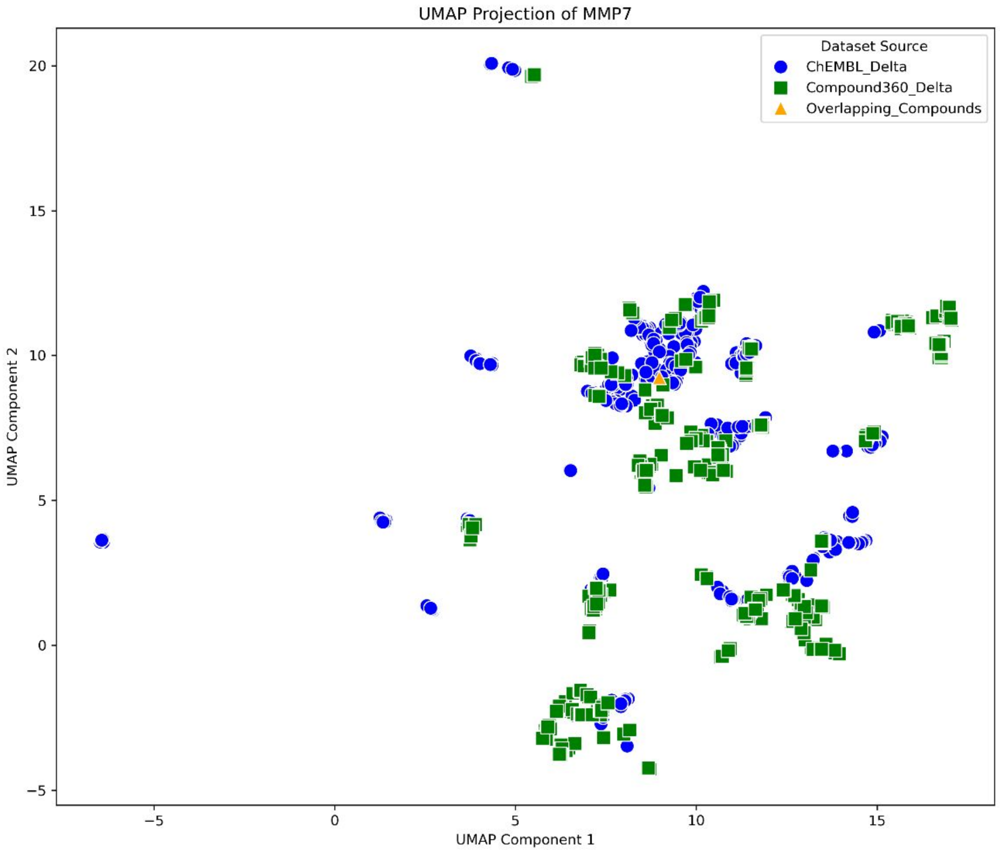
UMAP Projection of MMP7
Dataset Source
ChEMBL_Delta
Compound360_Delta
Overlapping_Compounds
UMAP Component 2
UMAP Component 1
20
15
10
5
0
−5
−5
0
5
10
15

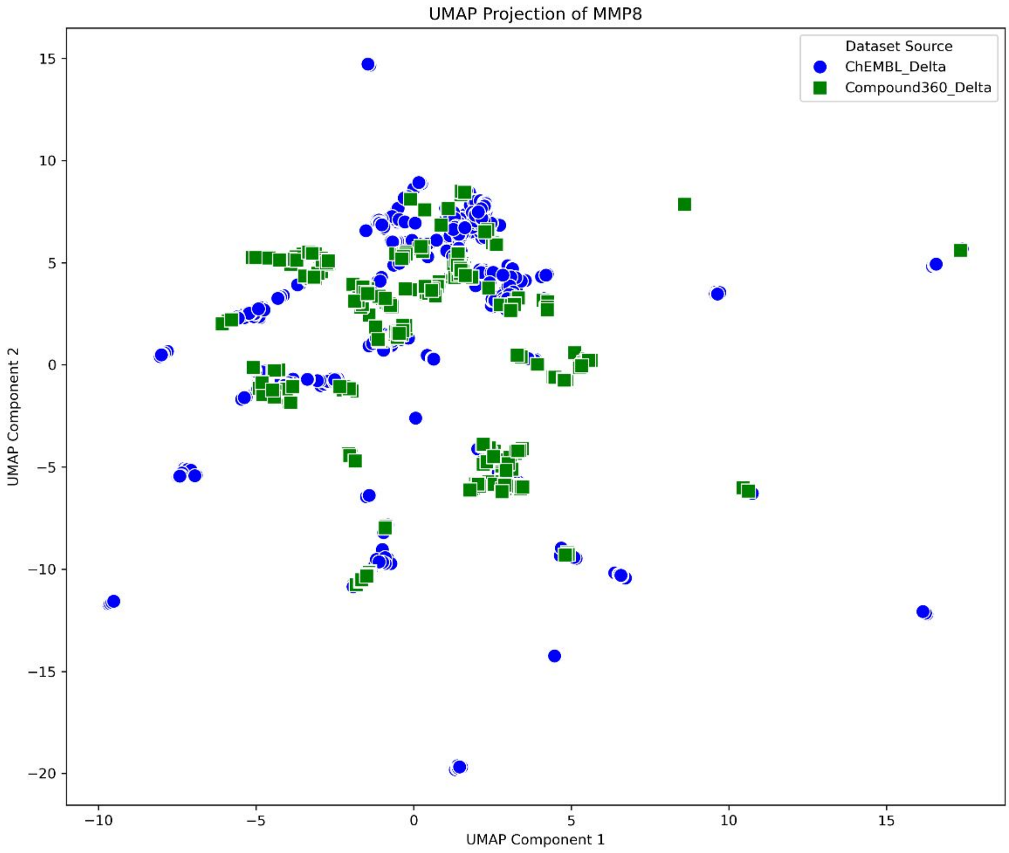

UMAP Projection of MMP8
Dataset Source
ChEMBL_Delta
Compound360_Delta
UMAP Component 2
UMAP Component 1
15
10
5
0
−5
−10
−15
−20
−10
−5
0
5
10
15

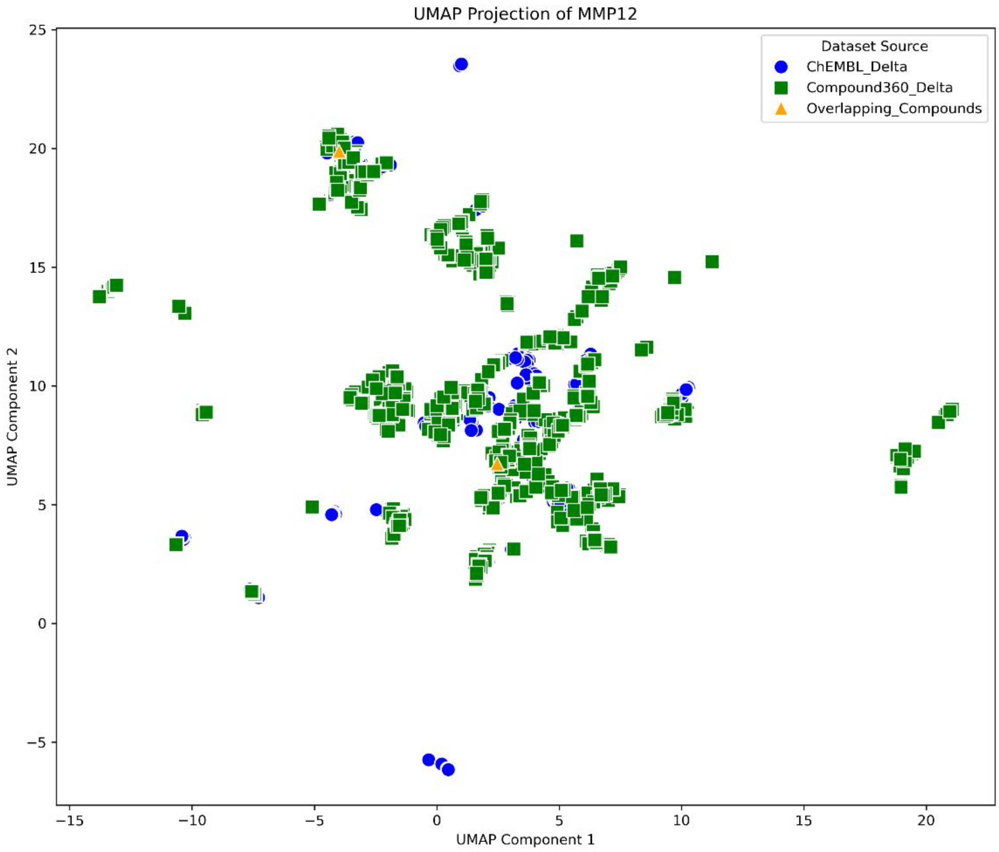

UMAP Projection of MMP12
Dataset Source
ChEMBL_Delta
Compound360_Delta
Overlapping_Compounds
UMAP Component 1
UMAP Component 2
−15
−10
−5
0
5
10
15
20
25

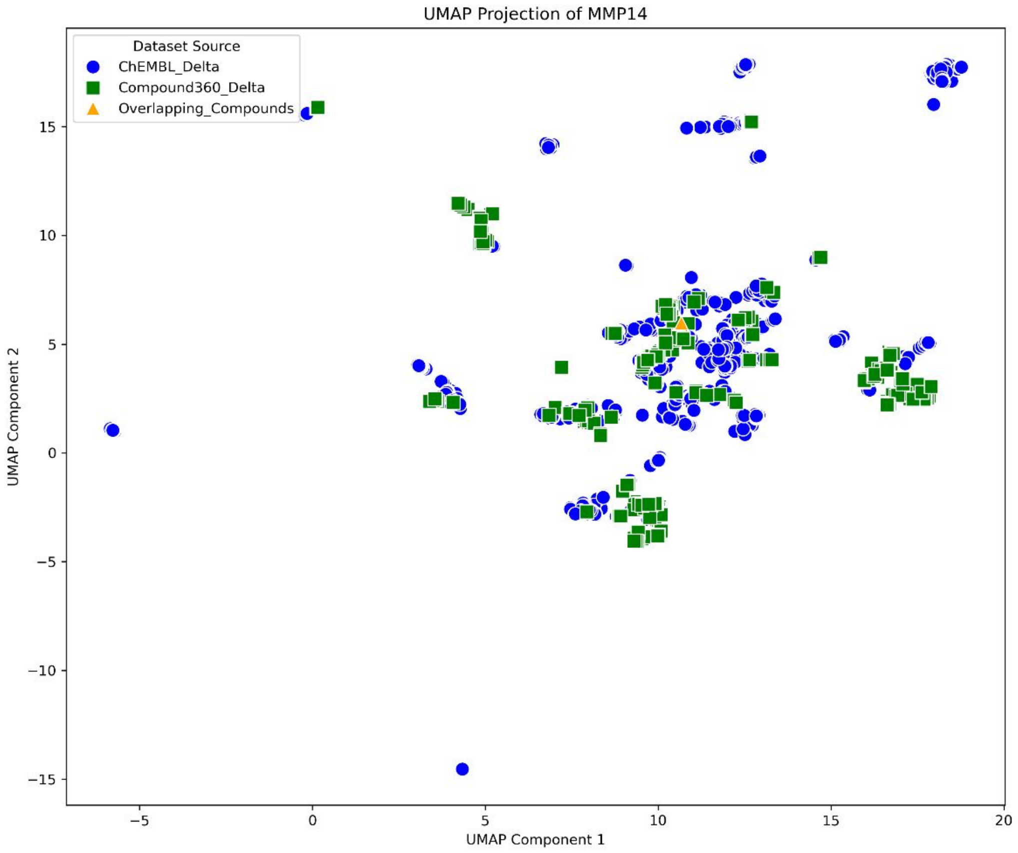
UMAP Projection of MMP14
Dataset Source
ChEMBL_Delta
Compound360_Delta
Overlapping_Compounds
UMAP Component 2
UMAP Component 1
15
10
5
0
−5
−10
−15
−5
0
5
10
15
20

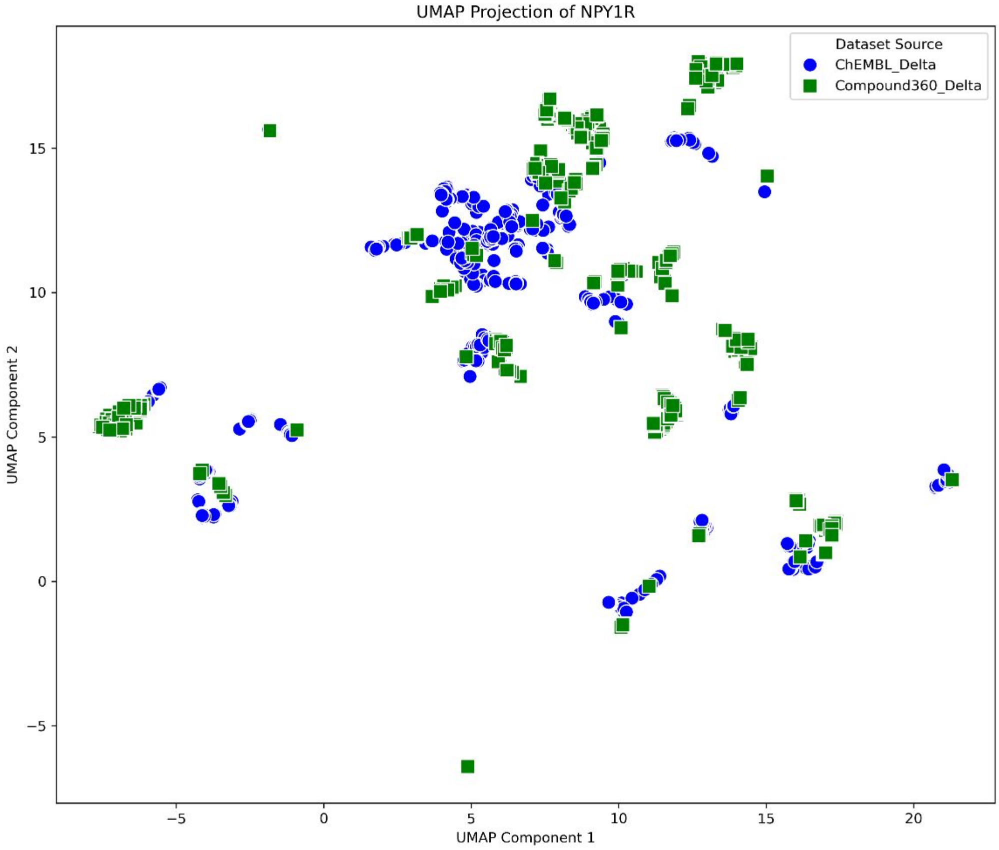
UMAP Projection of NPY1R
Dataset Source
ChEMBL_Delta
Compound360_Delta
UMAP Component 1
UMAP Component 2
−5
0
5
10
15
20
−5
0
5
10
15

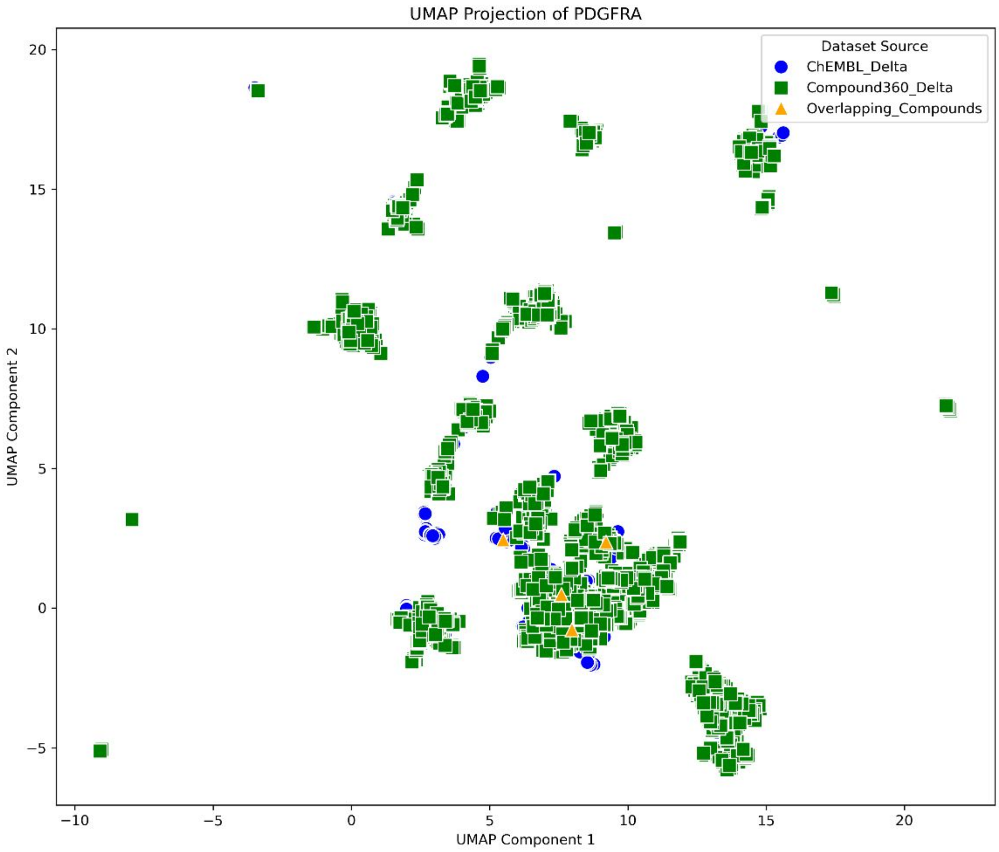

UMAP Projection of PDGFRA
Dataset Source
ChEMBL_Delta
Compound360_Delta
Overlapping_Compounds
UMAP Component 1
UMAP Component 2
20
15
10
5
0
−5
−10
−5
0
5
10
15
20

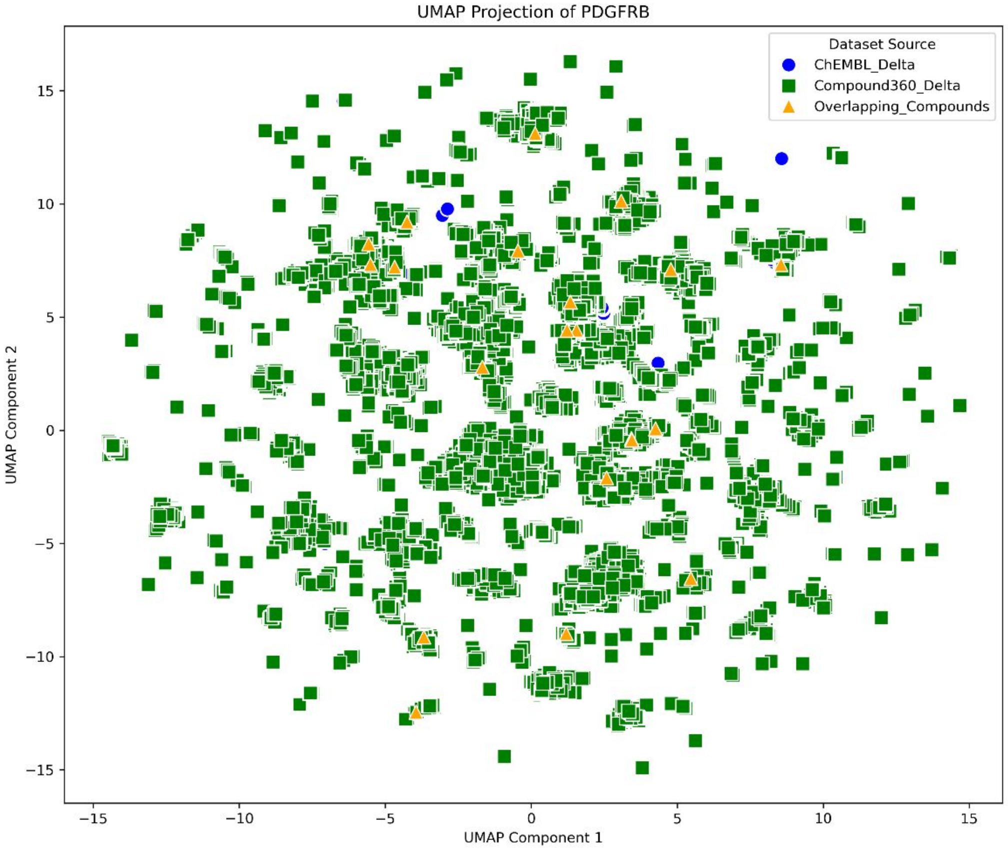
UMAP Projection of PDGFRB
Dataset Source
ChEMBL_Delta
Compound360_Delta
Overlapping_Compounds
UMAP Component 2
UMAP Component 1
15
10
5
0
−5
−10
−15
−15
−10
−5
0
5
10
15

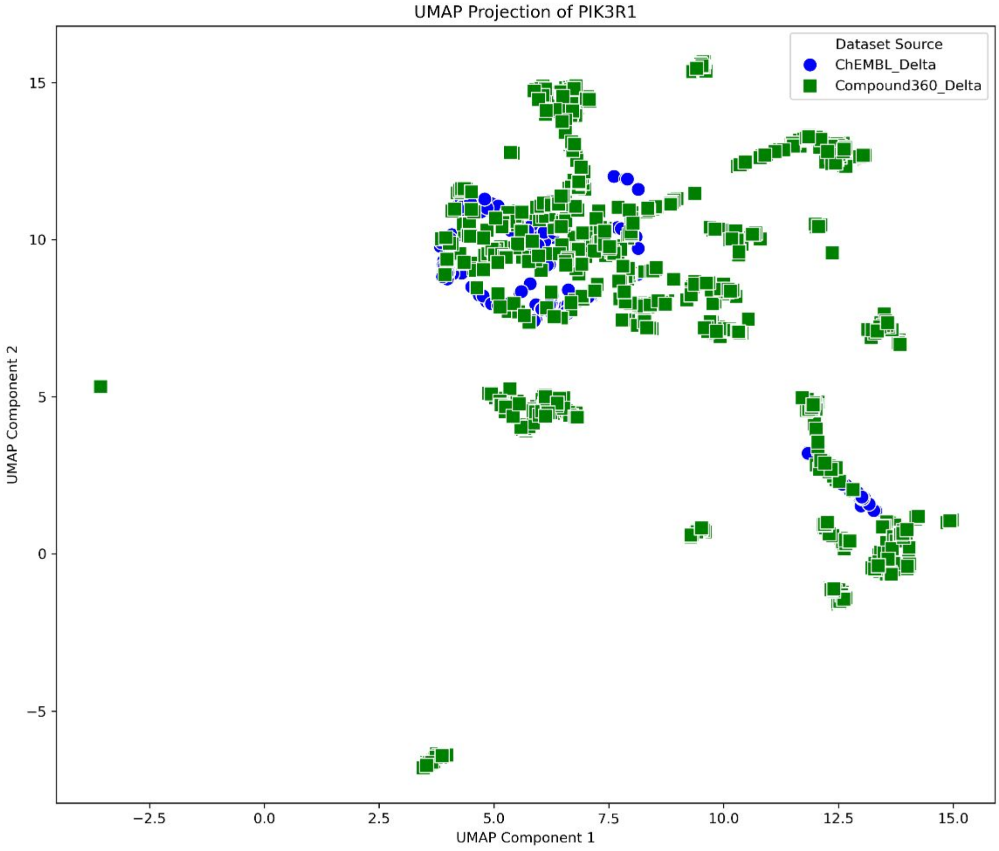

UMAP Projection of PIK3R1
Dataset Source
ChEMBL_Delta
Compound360_Delta
UMAP Component 1
UMAP Component 2
−2.5
0.0
2.5
5.0
7.5
10.0
12.5
15.0
−5
0
5
10
15

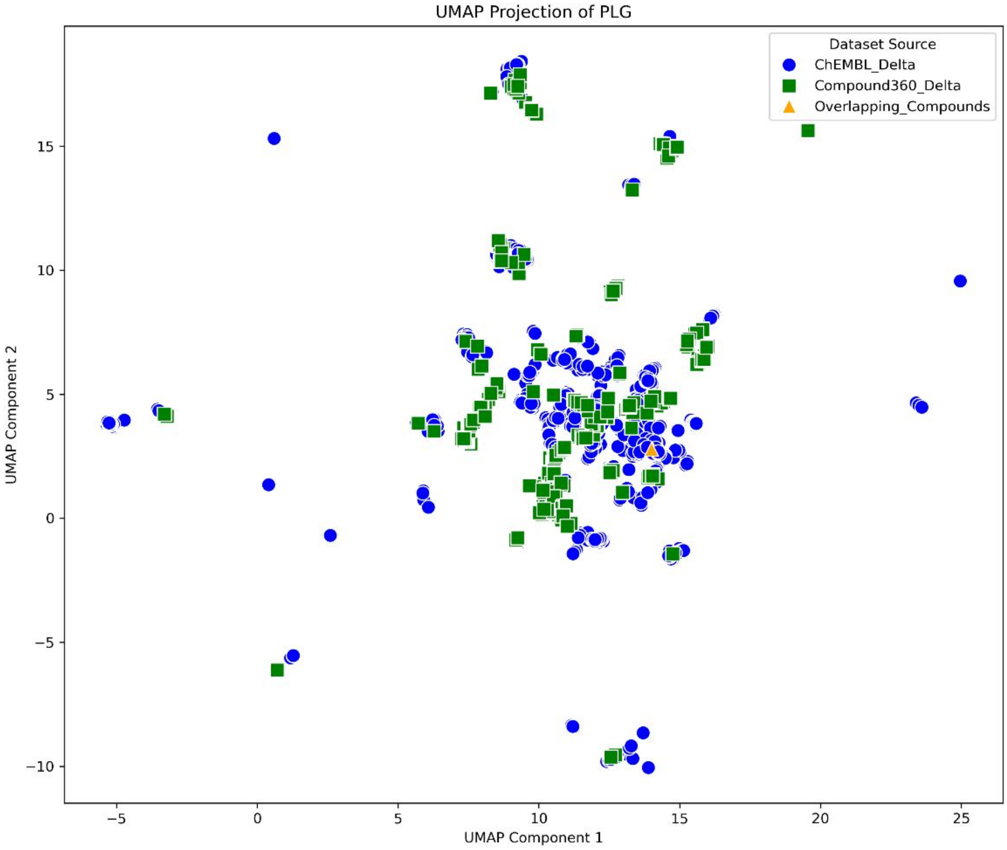
UMAP Projection of PLG
Dataset Source
ChEMBL_Delta
Compound360_Delta
Overlapping_Compounds
UMAP Component 1
UMAP Component 2
15
10
5
0
−5
−10
−5
0
5
10
15
20
25

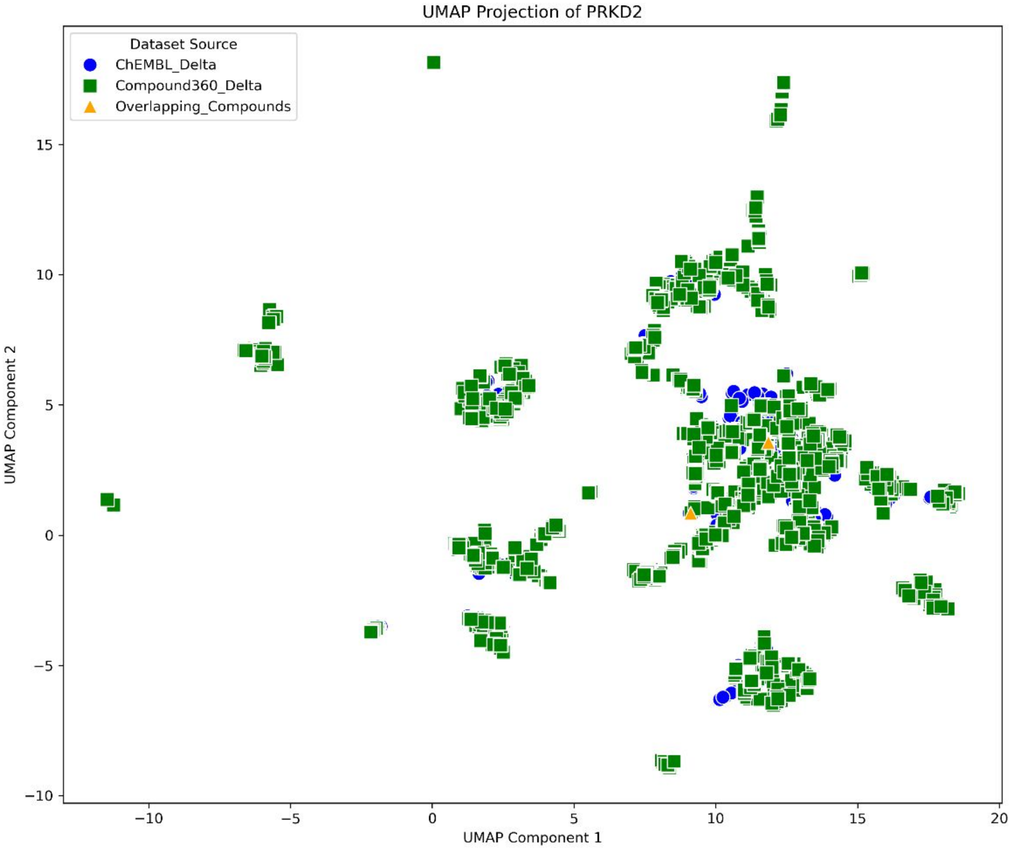
UMAP Projection of PRKD2
Dataset Source
ChEMBL_Delta
Compound360_Delta
Overlapping_Compounds
UMAP Component 2
UMAP Component 1
15
10
5
0
−5
−10
−10
−5
0
5
10
15
20

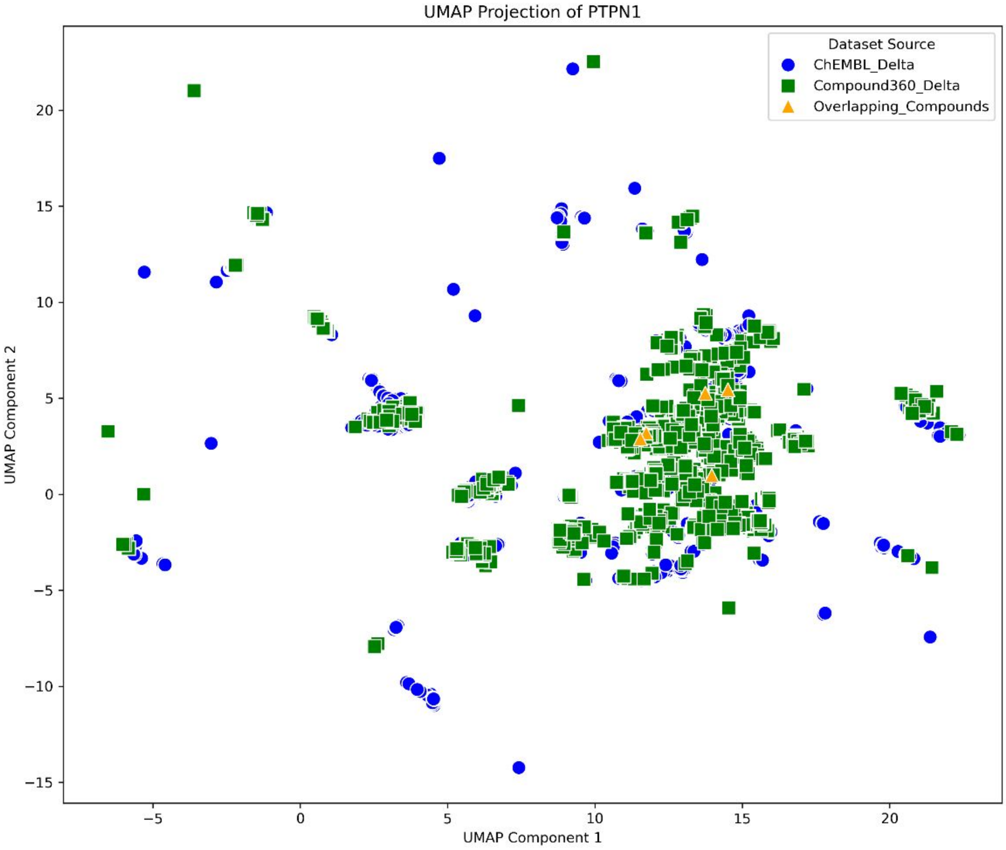
UMAP Projection of PTPN1
Dataset Source
ChEMBL_Delta
Compound360_Delta
Overlapping_Compounds
UMAP Component 2
UMAP Component 1
20
15
10
5
0
−5
−10
−15
−5
0
5
10
15
20

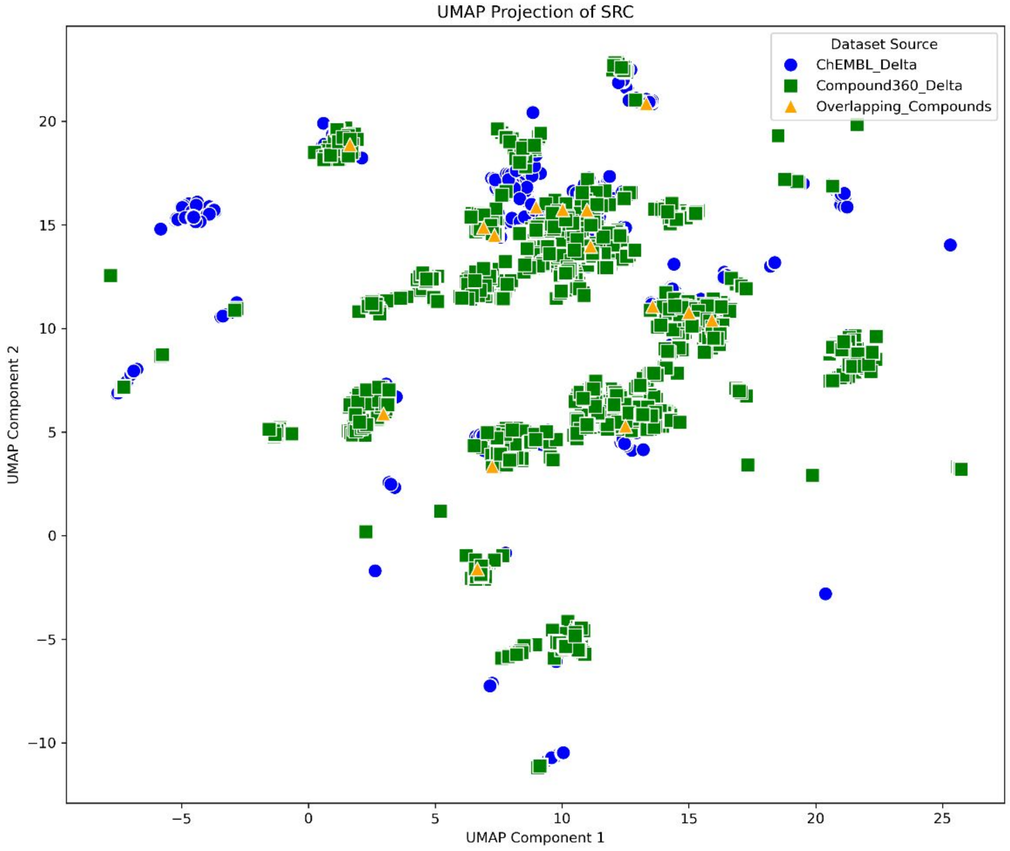

UMAP Projection of SRC
Dataset Source
ChEMBL_Delta
Compound360_Delta
Overlapping_Compounds
UMAP Component 1
UMAP Component 2
−10
−5
0
5
10
15
20
25

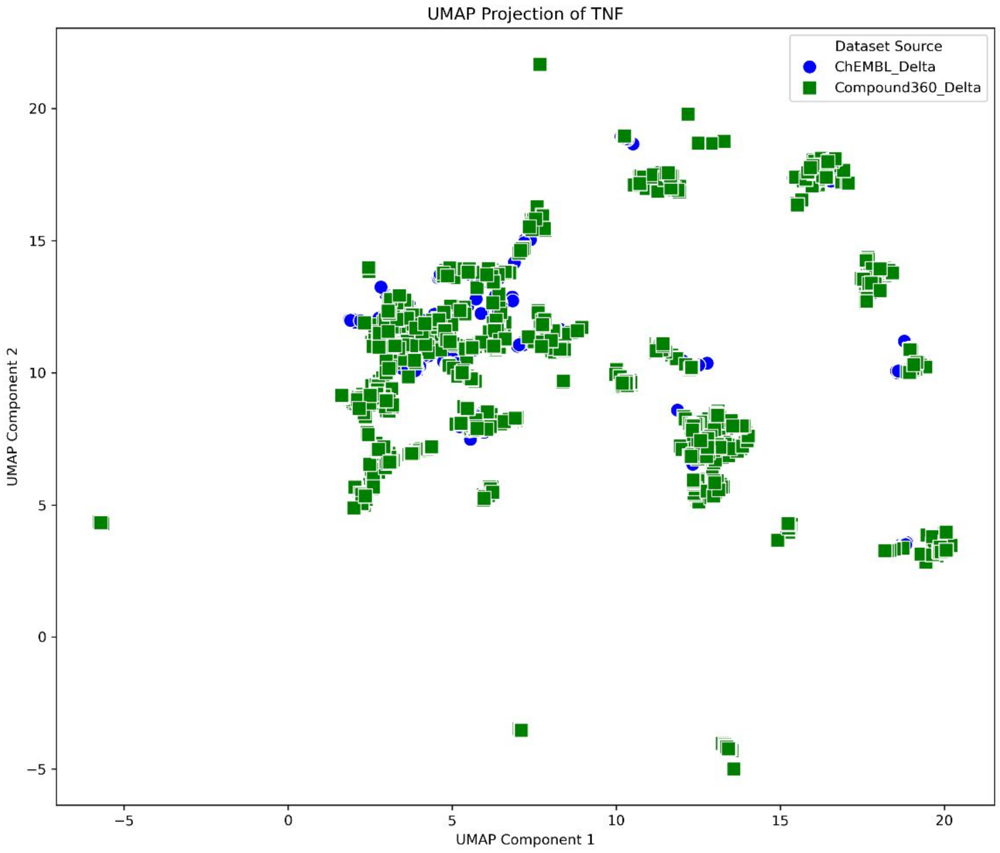
UMAP Projection of TNF
Dataset Source
ChEMBL_Delta
Compound360_Delta
UMAP Component 1
UMAP Component 2

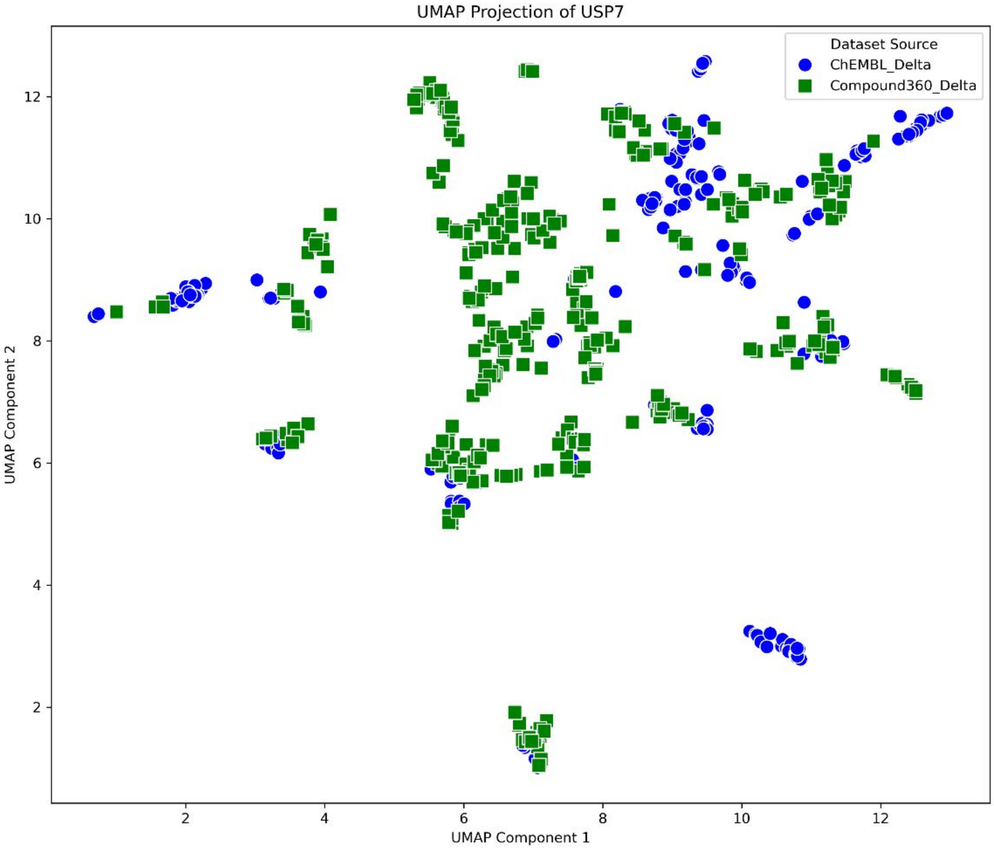

Figure S13: UMAP visualization of compounds from different data set sources for the 40 targets using Estate descriptors. Overlapping compounds are indicated as yellow triangles. Blue dots represent the ChEMBL chemical space whereas green squares represent Bayer AG internal chemical space.
